# Synergistic catalysis on Fe–N_*x*_ sites and Fe nanoparticles for efficient synthesis of quinolines and quinazolinones *via* oxidative coupling of amines and aldehydes†
†Electronic supplementary information (ESI) available: Experimental section, supplementary figures and tables, and ^1^H/^13^C NMR spectroscopy and HR-MS data for all compounds. See DOI: 10.1039/c9sc04060a


**DOI:** 10.1039/c9sc04060a

**Published:** 2019-09-23

**Authors:** Zhiming Ma, Tao Song, Youzhu Yuan, Yong Yang

**Affiliations:** a Qingdao Institute of Bioenergy and Bioprocess Technology , Chinese Academy of Sciences , Qingdao 266101 , P. R. China; b University of Chinese Academy of Sciences , Beijing , 100049 , P. R. China; c State Key Laboratory of Physical Chemistry of Solid Surface , National Engineering Laboratory for Green Chemical Productions of Alcohols-Ethers-Esters , College of Chemistry and Chemical Engineering , Xiamen University , Xiamen 361005 , P. R. China

## Abstract

In this paper, we developed a reusable heterogeneous non-precious iron nanocomposite comprising metallic Fe–Fe_3_C nanoparticles and Fe–N_*x*_ sites on N-doped porous carbon, which allows for highly efficient synthesis of quinolines and quinazolinones *via* oxidative coupling of amines and aldehydes using H_2_O_2_ as the oxidant in aqueous solution under mild conditions. A set of quinazolines and quinazolinones were synthesized in high yields with a broad substrate scope and good tolerance of functional groups. Characterization and control experiments disclose that a synergistic effect between the metallic Fe nanoparticles and built-in Fe–N_*x*_ sites is primarily responsible for the outstanding catalytic performance. Furthermore, the iron nanocomposite could be readily recovered for successive use without appreciable loss in catalytic activity and selectivity. This work provides an expedient and sustainable method to access pharmaceutically relevant N-heterocycles.

## Abstract 2

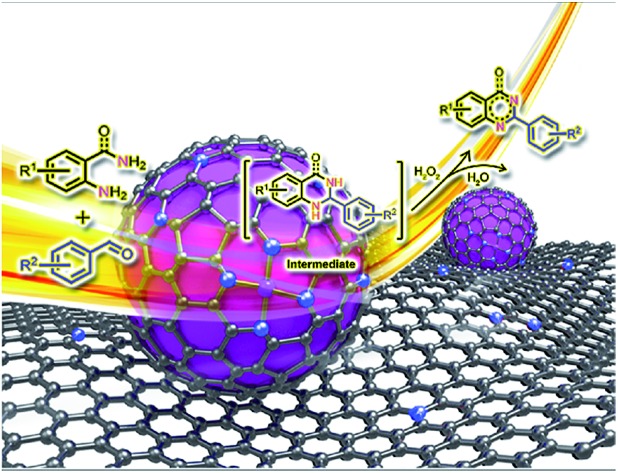
A heterogeneous nanocomposite of Fe–Fe_3_C nanoparticles and Fe–N_*x*_ sites on N-doped porous carbon allows for efficient synthesis of quinolines and quinazolinones *via* oxidative coupling of amines and aldehydes in aq. solution using H_2_O_2_ as the oxidant.

## Introduction

The development of reusable earth-abundant and inexpensive non-precious metal catalysts for innovative organic synthesis is a key technology for a more sustainable production of fine chemicals, pharmaceuticals and agrochemicals. Conventional nanostructured non-precious metal catalysts prepared by impregnation or immobilization are generally only applicable for organic transformations of structurally simple molecules. To further explore their broad application for more challenging and complex synthetic reactions, great efforts have been devoted to rational design and fabrication of nanostructured non-precious metal catalysts with higher potential over the past few decades. Consequently, a number of effective nanostructured non-precious metal (*e.g.*, Fe,^1^ Co,^2^ Ni,^3^ and Mn^4^) catalysts with unique structures or compositions have been developed *via* pyrolysis of either a mixture of metal complexes and/or carbon support1c–e,2c,2e,f,3b,e (or Al_2_O_3_ (ref. 1*f* and 2*b*) or SiO_2_ (ref. 2*c* and 3*f*)), or a mixture of metal salts and renewable biomass,^14^ or metal–organic frameworks (MOFs).2g–i The resulting nanostructured catalysts have demonstrated excellent catalytic performance for a set of organic reactions, such as reductive amination,3a,d,e hydrogenation of nitroarenes,1a,e,2d,e,3c,f the synthesis of nitriles,1g,2j and oxidation of alcohols2k,l,4a,c–e and N-heterocycles.1d,2m

Among them, nanostructured Fe catalysts are much more attractive due to the earth-abundant, non-toxic, biocompatible, and environmentally benign characteristics of Fe. Specifically, Fe–nitrogen-coordinated carbon catalysts, named Fe–N–C, have recently emerged as a fascinating catalyst for electrocatalysis, in which Fe–N_*x*_ sites are arguably considered as catalytically active sites.^5^ Yet, the exploration of Fe–N–C for organic synthesis is still scarce to date.1*c* Furthermore, recent studies disclosed the presence of Fe–N_*x*_ sites in hybrid nanostructured Fe catalysts which were proposed to be responsible for high catalytic reactivity,1*e* while no clear and solid evidence was observed or intense investigation was done to support such a hypothesis so far. As such, elucidation of the role of Fe–N_*x*_ sites in catalysis is urgently desirable, not only for better understanding the reactions but also for the rational design and preparation of highly active and stable nanostructured Fe–N–C catalysts.

**Scheme 1:**
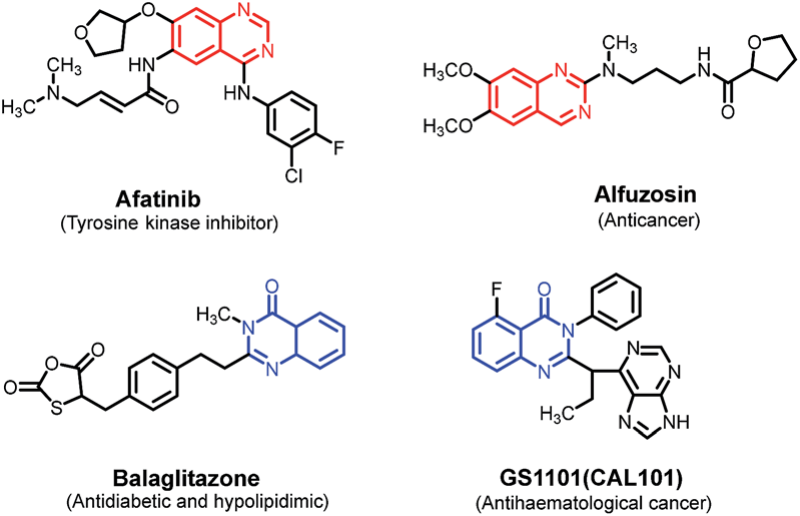
Selected examples of market available drugs with quinolone and quinazolinone skeletons.

N-heterocycles are ubiquitous in nature and constitute the backbone of numerous natural products, pharmaceutically important molecules, and organic functional materials.^6^ Among various N-heterocycles known, quinazolines and quinazolinones are two classes of fused structural motifs with a wide range of pharmacological and biological activities, such as antibacterial, anti-inflammatory, anticonvulsant, antimalarial, antiasthmatic, anti-Alzheimer, and anticancer,^7^ and are found in many drugs available on the market (Scheme 1).

Given the importance of N-heterocycles, a number of synthetic methods have been developed over the past few decades.^8^ Despite these significant advances, the most classical and general approaches for the synthesis of quinazolines and quinazolinones still strongly rely on the condensation between *o*-aminobenzylamines and aldehydes followed by the oxidation of the resulting aminal intermediates in the laboratory and industry. However, this protocol generally requires the use of a large excess of toxic oxidants, such as DDQ,^9^ MnO_2_,^10^ PhI(OAc)_2_,^11^ and NaClO,^12^ or homogeneous transition metal complexes ligated with well-defined ligands,8d,^13^ which significantly limit its practical application, especially for pharmaceutical synthesis. Therefore, the development of efficient, stable and cost-effective heterogeneous non-precious metal catalysts for accessing quinazolines and quinazolinones is highly desirable.

In this work, we develop a novel nanostructured iron catalyst derived from pyrolysis of a mixture of iron salt and readily available and renewable N-containing biomass, bamboo shoots, in a facile preparation method. The resultant catalysts comprise mixed phases, including metallic Fe and Fe_3_C nanoparticles (NPs) and Fe–N_*x*_ sites, which exhibited excellent catalytic activity for the oxidative coupling of amines and aldehydes to access N-heterocycles using H_2_O_2_ as a green and sustainable oxidant in water under mild conditions. A set of pharmaceutically relevant quinazolines and quinazolinones were synthesized in high yields with a broad substrate scope and good tolerance of functional groups. Further studies reveal that synergistic catalysis on Fe/Fe_3_C NPs and Fe–N_*x*_ sites is primarily responsible for the high efficiency of the reactions. Moreover, the catalyst could be easily recycled several times without significant loss in catalytic activity.

## Results and discussion

Nanostructured iron catalysts were prepared in a facile sequential hydrothermal-pyrolysis process according to a similar procedure as that reported by us.^14^ The biochar obtained from hydrothermal treatment of bamboo shoots was homogeneously mixed with Fe(NO_3_)_3_ in aqueous solution at 60 °C for 2 h. After evaporation of water, the solid powder was pyrolyzed under a constant nitrogen flow at 800 °C for 2 h (see details in the ESI†). The as-prepared catalyst was denoted as Fe–Fe_3_C@NC-800. For comparison, Fe–Fe_3_C@NPC-700 and 900 pyrolyzed at 700 and 900 °C were also prepared with the same preparation procedure. The Fe content in the catalysts was determined to be 4.29–4.51 wt% by inductively coupled plasma optical emission spectrometry (ICP-OES) (Table S1†).

We initiated our studies of the synthesis of quinazolines *via* the oxidative coupling of 2-aminobenzylamine (**1a**) with benzaldehyde (**2a**) as a model reaction using Fe–Fe_3_C@NC-800 as the catalyst to optimize the reaction conditions (Table 1). A set of parameters including the addition amount of benzaldehyde or the oxidant, solvents and reaction temperatures were intensively screened. The reaction was first performed using 1.2 equivalents of benzaldehyde (with respect to **1a**) in the absence of an oxidant in H_2_O at 100 °C. 95% conversion of **1a** was observed, affording 1,2,3,4-tetrahydro-2-phenylquinazoline intermediate (**I**) as the major product with only 7% GC yield of the desired 2-phenylquinazoline **3a** (entry 1). To our delight, 78% GC yield of **3a** was obtained when 2.0 equivalents of H_2_O_2_ as the oxidant were used under otherwise identical conditions (entry 2). A further increase of the amount of H_2_O_2_ resulted in a gradual decrease in the yield of the desired **3a** (Table S2†). 1.2 equivalents of benzaldehyde was found to be the most appropriate ratio for the synthesis of **3a** in terms of catalytic activity and selectivity (Table S3†). A decrease of either the loading of the catalyst Fe–Fe_3_C@NC-800 or reaction temperature led to a lower yield of **3a** (entries 3, 4, and 6). Further studies show that a mixture H_2_O–THF (v/v, 4/1) as the solvent could pronouncedly improve the catalytic efficiency, and the yield of **3a** could reach as high as 95% under otherwise identical conditions (entry 5 and Table S4†). For comparison, the catalysts Fe–Fe_3_C@NC-700 and Fe–Fe_3_C@NC-900 were employed for the reaction, and both showed a relatively lower activity (entries 7 and 8). In addition, control experiments employing commercially available Fe_2_O_3_, Fe_3_O_4_, Fe(NO_3_)_3_, iron phthalocyanine (Fe(ii)Pc), and nano Fe powder as catalysts for the reaction show that all exhibited inferior reactivity (entries 9–13). However, in the absence of the catalyst, the reaction took place sluggishly to produce intermediate **I** as the major product (entry 14). These observations clearly indicate that the combination of the catalyst Fe–Fe_3_C@NC-800 and H_2_O_2_ as the oxidant is essential for the successful synthesis of 2-phenylquinazoline in high yield.

**Table 1:** Optimization of reaction conditions^*a*^

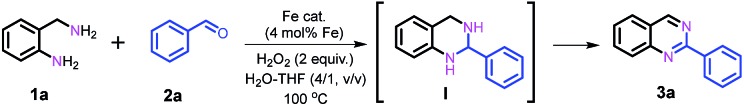					
Entry	Catalyst (Fe mol%)	Solvent	Conversion^*b*^ (%)	GC yield^*b*^ (%)	
				**I**	**3a**
1^*c*^	Fe–Fe_3_C@NC-800	H_2_O	95	88	7
2	Fe–Fe_3_C@NC-800	H_2_O	96	18	78
3^*d*^	Fe–Fe_3_C@NC-800	H_2_O	83	44	39
4^*e*^	Fe–Fe_3_C@NC-800	H_2_O	71	36	35
5	Fe–Fe_3_C@NC-800	H_2_O–THF	100	5	95
6^*f*^	Fe–Fe_3_C@NC-800	H_2_O–THF	83	20	63
7	Fe–Fe_3_C@NC-700	H_2_O–THF	88	12	76
8	Fe–Fe_3_C@NC-900	H_2_O–THF	90	6	84
9	Fe_2_O_3_	H_2_O–THF	18	6	12
10	Fe_3_O_4_	H_2_O–THF	29	3	26
11	Fe(NO_3_)_3_	H_2_O–THF	46	3	43
12	Nano Fe	H_2_O–THF	34	2	32
13	Fe(ii)Pc	H_2_O–THF	89	40	49
14^*g*^	—	H_2_O–THF	15	12	3

^*a*^Reaction conditions: 2-aminobenzylamine (**1a**) (0.2 mmol), benzaldehyde (**2a**) (0.24 mmol), catalyst (4 mol% of Fe), H_2_O_2_ (2 equivalents with respect to **1a**, 30 wt% in H_2_O), H_2_O (5 mL) or H_2_O–THF (5 mL, 4/1, v/v), 100 °C, 12 h.

^*b*^Determined by GC and GC-MS using 1,3,5-trimethyl-benzene as an internal standard sample and confirmed with their corresponding authentic samples.

^*c*^In the absence of an oxidant.

^*d*^80 °C.

^*e*^60 °C.

^*f*^Fe-Fe_3_C@NC-800 (2 mol% of Fe).

^*g*^In the absence of a catalyst.

**Fig. 1:**
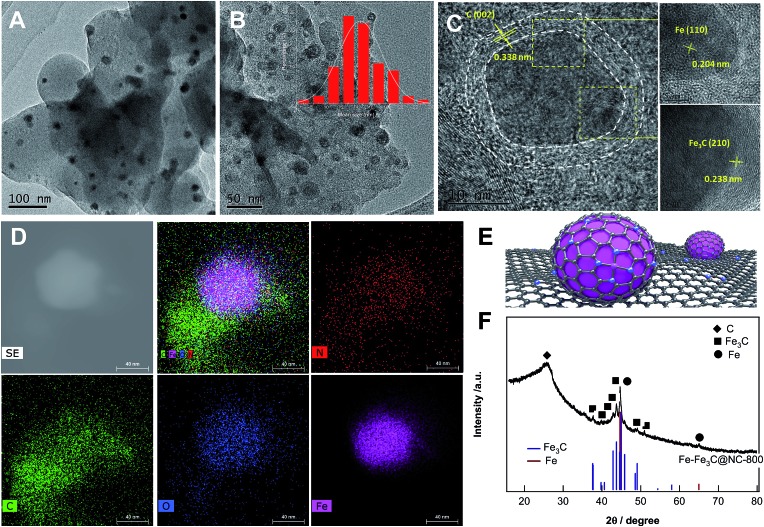
(A) TEM and (B and C) HR-TEM images of the catalyst Fe–Fe_3_C@NC-800; the inset shows the size distribution of metallic Fe nanoparticles. (D) HAADF-STEM and the corresponding EDX elemental mappings of individual Fe–Fe_3_C@NC-800, (E) schematic illustration of the catalyst Fe–Fe_3_C@NC-800, and (F) XRD pattern of the catalyst Fe–Fe_3_C@NC-800.

Given such impressive findings, we next investigated the structural properties of the catalyst Fe–Fe_3_C@NC-800 by means of comprehensive technical skills. The transmission electron microscope image (Fig. 1A) shows that nanoparticles with an average size of 14 nm are homogeneously dispersed on carbon. High resolution TEM images (Fig. 1B and C) further reveal that mixed metallic Fe and Fe_3_C NPs as the core were covered with a few layers of a graphitic carbon shell as shown in Fig. 1E. The well-resolved lattice spacing of 0.204, 0.238, and 0.338 nm is consistent with the Fe (110), Fe_3_C (210), and graphitic C (002) planes, respectively. High-angle annular dark-field scanning transmission electron microscopy (HAADF-STEM) images (Fig. 1D) demonstrate the homogeneous distribution of Fe, N, O and C atoms over the entire sample. The X-ray diffraction (XRD) pattern (Fig. 1F) discloses the formation of crystalline phases of metallic Fe and Fe_3_C with the appearance of characteristic diffraction peaks at 44.7° and 65° and at 37.6°, 37.7°, 39.8°, 40.6°, 42.9°, 43.7°, 44.9°, 45.9° and 49.1°, corresponding to the (110) and (200) phases of cubic metallic Fe (JCPDS no. 06-0696) and the (121), (210), (002), (201), (211), (102), (220), (031), (112), (221) planes of Fe_3_C (cementite, JCPDS no. 35-0772), respectively. Besides, a broad bump peak at 26.1° together with a tiny peak at 43.1° indicates the formation of graphitic carbon upon pyrolysis at 800 °C. These observations are in good agreement with HR-TEM results. The Raman spectrum (Fig. S2†) provided solid evidence for the formation of graphitic carbon with certain graphitization and defect sites. N_2_ adsorption/desorption measurements (Fig. S3†) demonstrate that the catalyst Fe–Fe_3_C@NC-800 possess hierarchically micro-, meso-, and macropores with a high specific surface area and large pore volume (Table S1†).

**Fig. 2:**
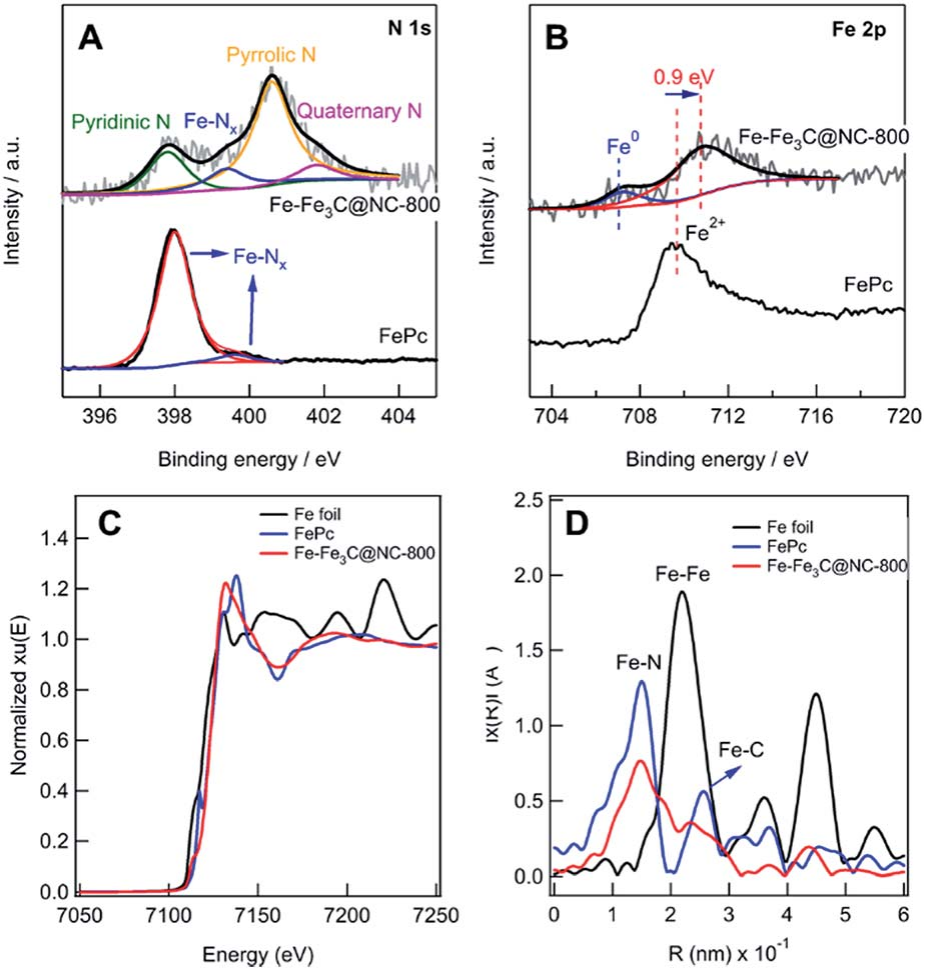
(A) The deconvoluted N 1s and (B) Fe 2p spectra of FePc and the catalyst Fe–Fe_3_C@NC-800. (C) XANES spectra and (D) Fourier transform (FT) of the Fe K-edge EXAFS data of the catalyst Fe–Fe_3_C@NC-800, Fe foil and FePc.

The N 1s XPS spectrum (Fig. 2A) shows 4 deconvoluted peaks at 398.2, 399.6, 400.4, and 401.3 eV, which are assignable to pyridinic, Fe–N_*x*_, pyrrolic, and quaternary N, respectively.5i,14c,d The Fe 2p XPS spectrum (Fig. 2B) shows 3 peaks, and the peak at 706.8 eV in the Fe 2p_3/2_ spectrum can be attributed to zero-valence Fe (metallic iron or carbide), while the peak at 710.4 eV can be assigned to Fe in the Fe(ii)–N_*x*_ configuration.5c–e Compared with Fe(ii)Pc, 0.9 eV shift to a higher value was observed, implying the interaction of Fe–N_*x*_ and metallic Fe NPs.5h,^15^ To further investigate the local iron structure of the catalyst Fe–Fe_3_C@NC-800, X-ray absorption spectroscopy (XAS) was performed. The spectroscopy of Fe K-edge X-ray absorption near edge structure (XANES) reveals that the catalyst Fe-Fe_3_C@NC-800 almost overlaps with that of Fe(ii)Pc, indicating the possible remaining Fe–N_4_ structure (Fig. 2C), which is further confirmed from the Fe K-edge extended X-ray absorption fine structure (EXAFS) in Fig. 2D. From the shape and amplitude of the first strong peak at ≈1.5 Å, it is clear that the bonding environment in the first shell of the catalyst Fe–Fe_3_C@NC-800 is notably similar to that of Fe(ii)Pc, suggesting that it more likely contains FeN_4_ complex structures.5h,^16^ Besides, the peaks at ≈2.1 Å, assignable to Fe–Fe interactions, reveals the presence of an iron-based crystalline structure in the catalyst Fe–Fe_3_C@NC-800.

**Fig. 3:**
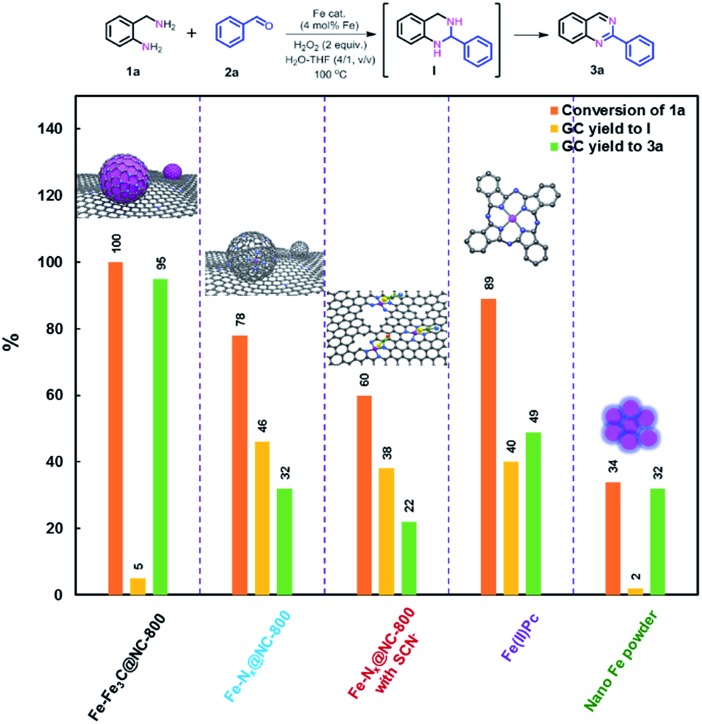
Comparison of catalytic performance over different catalysts for the benchmark reaction.

Taking all characterization results into account, we can conclude that the as-prepared catalyst Fe–Fe_3_C@NC-800 comprises core–shell structured nanoparticles with metallic Fe and Fe_3_C NPs as the core and layers of graphitic carbon as the shell and coordinated Fe–N_*x*_ sites as well. To unveil the catalytically active sites for the oxidative coupling reaction, a set of control experiments were carried out. First, the catalyst Fe–Fe_3_C@NC-800 was leached with acid to remove the metallic Fe NPs, denoted as Fe–N_*x*_@NC-800 (see details in the ESI†). The HRTEM images and XRD pattern of the acid-etched catalyst Fe–N_*x*_@NC-800 (Fig. S7 and S8†) show that no nanoparticles were found with preserved hollow-centered graphitic carbon layers. Such a finding further verifies the core–shell structure of the catalyst Fe–Fe_3_C@NC-800. When the acid-etched catalyst Fe–N_*x*_@NC-800 was subjected to the optimized reaction conditions for the benchmark reaction, a remarkable decrease in both conversion of **1a** and yield of **3a** was observed, as shown in Fig. 3. This result indicates that metallic Fe NPs are necessary for the high catalytic activity, especially for the oxidative dehydrogenation to form the aromatized product **3a**. It is known that SCN^–^ ions can poison Fe–N_*x*_ sites in catalysis.1c,5h Second, the benchmark reaction was performed using the acid-etched catalyst Fe–N_*x*_@NC-800 with the addition of NaSCN under otherwise identical conditions. In this case, a further decrease in activity was achieved, clearly implying that Fe–N_*x*_ sites indeed boost the reaction. In parallel, Fe(ii)Pc is more active for coupling but with lower selectivity to **3a**, while nano Fe powder was just in opposite position (Fig. 3). In addition, the catalytic activity has a good correlation with the content of Fe–N_*x*_ in the catalysts Fe–Fe_3_C@NC-T as shown in Fig. S9,† that is, the higher the content of Fe–N_*x*_, the better the activity towards the desired product **3a**. As such, these results unambiguously corroborate that metallic Fe and Fe_3_C nanoparticles and coordinated Fe–N_*x*_ in the catalyst Fe–Fe_3_C@NC-800 are synergistically responsible for the coupling reaction to achieve excellent catalytic activity.

**Scheme 2:**
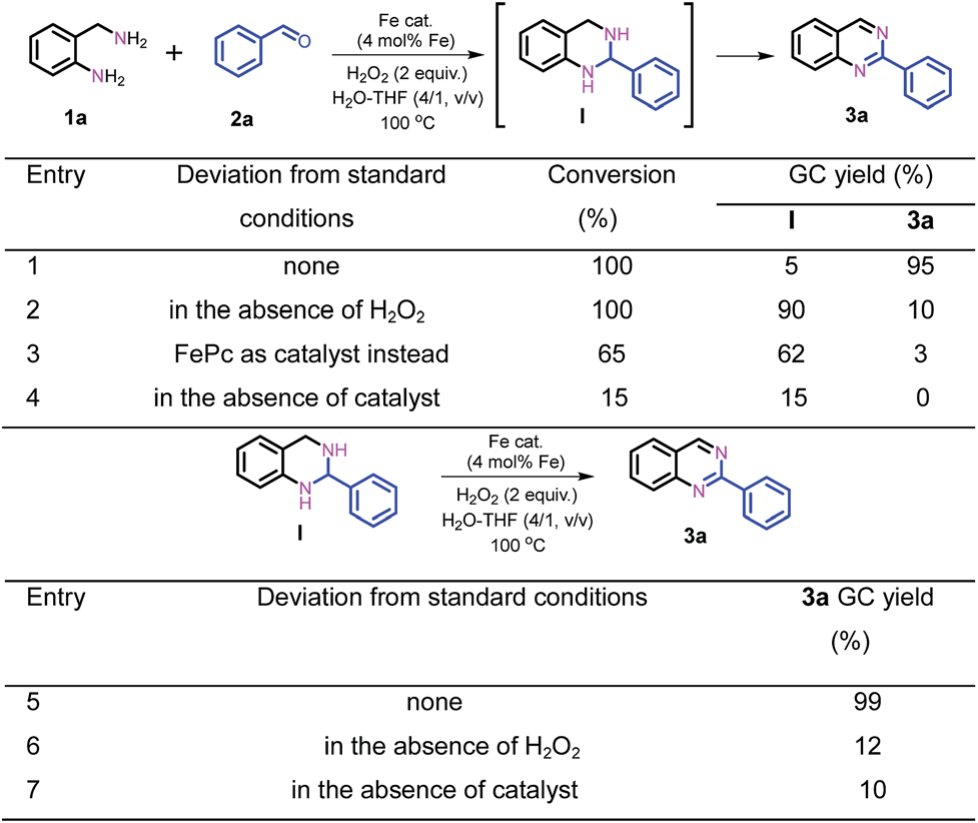
Elucidation of the individual role of the catalyst Fe–Fe_3_C@NC-800 and the oxidant H_2_O_2_.

Subsequently, further efforts were made to elucidate the individual role of the catalyst Fe–Fe_3_C@NC-800 and the oxidant H_2_O_2_ in this cascade coupling process. As stated above, the combination of the catalyst Fe–Fe_3_C@NC-800 and oxidant H_2_O_2_ is a prerequisite for the success of the coupling to afford the desired quinazoline **3a**, which was reinforced by the control experiments as shown in Scheme 2. In the absence of either the catalyst or oxidant, trace amounts of **3a** were achieved with the formation of intermediate **I** being the major product instead (Scheme 2, entries 2 and 4). Particularly, the coupling underwent sluggishly in the absence of H_2_O_2_, and only 15% of **1a** was converted. This observation clearly indicates the critical role of the catalyst Fe–Fe_3_C@NC-800 to efficiently boost the entire process. Furthermore, when the intermediate **I** was subjected to the standard conditions, quantitative conversion to **3a** was obtained (Scheme 2, entry 5). Once again, however, in the absence of either the catalyst or oxidant, the efficiency of the oxidative dehydrogenation is significantly low, yielding **3a** in 12% and 10% yield, respectively, under otherwise identical conditions (Scheme 2, entries 6 and 7). As such, we can safely conclude that the catalyst Fe–Fe_3_C@NC-800 participates in the condensation and oxidation steps and significantly facilitates the reaction, while the oxidant H_2_O_2_ benefits the dehydrogenation for aromatization to produce N-heterocycles.

After identifying the optimal reaction conditions and the catalytically active sites, we subsequently explored the generality of this protocol for the synthesis of 2-substituted quinazolines. As shown in Table 2, in general, various benzaldehydes bearing electron-donating and -withdrawing groups could efficiently couple with 2-aminobenzylamine (**1a**) to give their corresponding quinazolines in high yields, while benzaldehydes substituted by electron-donating groups (**2b–e**) gave relatively higher yields than those substituted with electron-withdrawing groups (**2j–m**). Halogen-substituted benzaldehydes, such as –F, –Cl and –Br, were tolerated under the present conditions, yielding the desired quinazolines (**2f–i**) in 85–95% yields. Heterocyclic aldehydes such as 3-thiophenecarboxaldehyde (**2o**) were also suitable as the coupling partner to deliver their corresponding quinazoline (**2o**) in 86% yield. In addition, aliphatic aldehydes, such as cyclohexanecarboxaldehyde (**2p**), cyclopropanecarboxaldehyde (**2q**) and heptaldehyde (**2r**), could also efficiently couple with **1a** to give the desired quinazolines **3p**, **3q** and **3r** in 83%, 91% and 93% yields, respectively.

**Table 2:** Substrate scope for the synthesis of quinazolines^*a*^

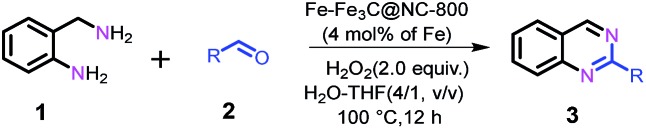
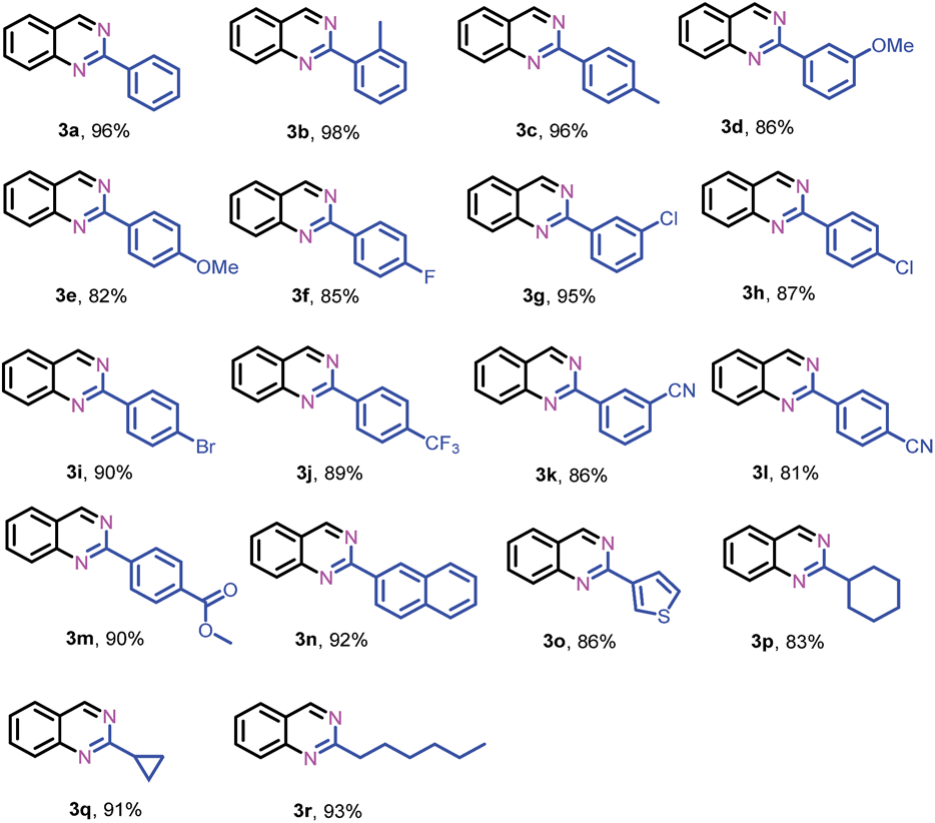

^*a*^Reaction conditions: 2-aminobenzylamine (**1a**) (0.2 mmol), aldehyde (0.24 mmol), Fe–Fe_3_C@NC-800 (4 mol% of Fe), H_2_O_2_ (2 equivalents with respect to **1a**, 30% in H_2_O), H_2_O–THF (5 mL, 4/1, v/v), 100 °C, 12 h. Yields of isolated product are reported.

Next, we further explored the substrate scope of the oxidative coupling of 2-aminobenzamide (**4a**) with various aldehydes (**2**) to synthesize quinazolinones under the optimal reaction conditions. To our delight, a broad spectrum of quinazolinones were successfully synthesized in high isolated yields as shown in Table 3. 2-Aminobenzamide could efficiently couple with benzyl aldehydes bearing electron-withdrawing and electron-donating groups as well as halogens. Likewise, heterocyclic and aliphatic aldehydes are also tolerated under the present conditions to deliver their corresponding quinazolinones in high yields. In addition, substituted 2-aminobenzamides are compatible for oxidative coupling too.

**Table 3:** Substrate scope for the synthesis of quinazolinones^*a*^

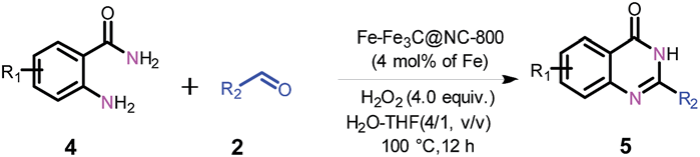
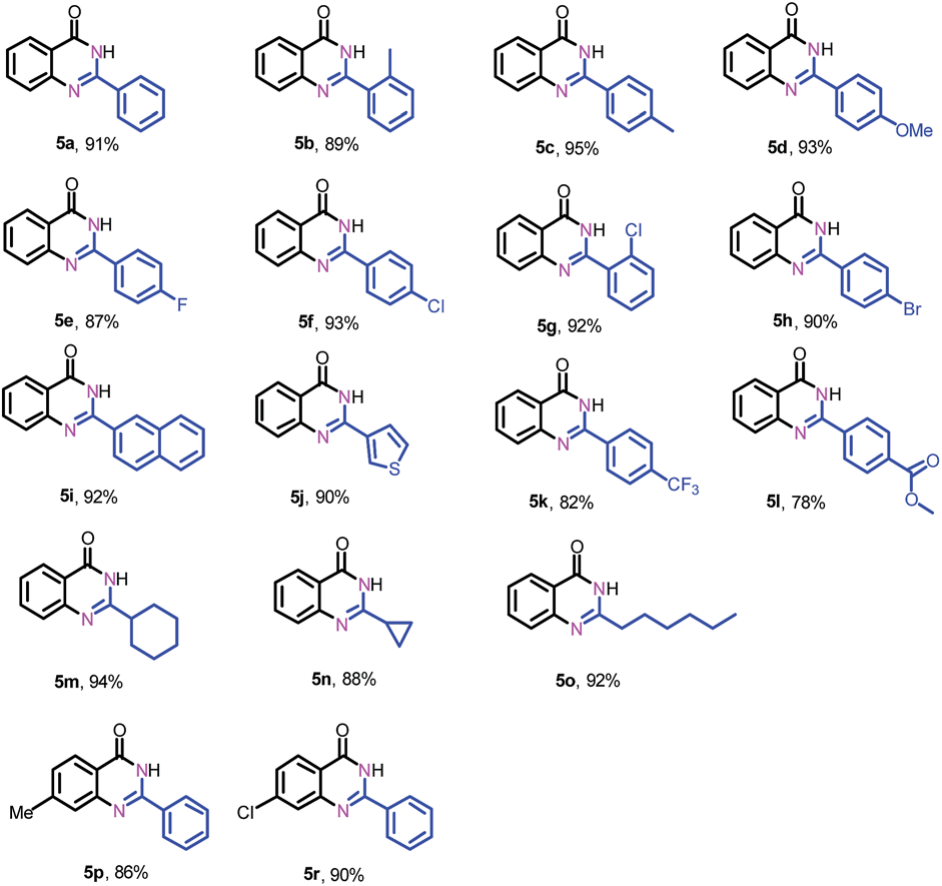

^*a*^Reaction conditions: 2-aminobenzamide (**4**) (0.2 mmol), aldehyde (0.24 mmol), Fe–Fe_3_C@NC-800 (4 mol% of Fe), H_2_O_2_ (4 equivalents with respect to **1a**, 30% in H_2_O), H_2_O–THF (5 mL, 4/1, v/v), 100 °C, 12 h. Yields of isolated product are reported.

Durability/recyclability of a heterogeneous catalyst is critical for sustainable and practical applications. To test the durability of Fe–Fe_3_C@NC-800, the used catalyst was collected, washed, and dried after completion of an oxidative coupling experiment for the synthesis of 2-phenylquinazoline (**2a**). As shown in Fig. S10,† the catalytic activity and selectivity remained high with negligible changes after six recycles, demonstrating the high durability of this catalyst.

## Conclusion

In conclusion, we developed a reusable heterogeneous earth-abundant iron nanostructured catalyst comprising metallic Fe and Fe_3_C nanoparticles as the core covered by a few layers of N-doped graphitic carbon and coordinated Fe–N_*x*_ sites as well in a facile and cost-effective manner. The resultant best catalyst Fe–Fe_3_C@NC-800 demonstrated excellent catalytic activity for oxidative coupling of amines with aldehydes to access a broad set of quinazolines and quinazolinones. The process was performed in a green and sustainable manner under mild reaction conditions with good tolerance of multifunctional groups. Moreover, the catalyst could be readily recovered for successive reuse without significant loss in activity and selectivity. Synergistic catalysis on Fe–N_*x*_ sites and metallic Fe–Fe_3_C nanoparticles is primarily responsible for the superior activity and stability. This work not only demonstrates the potential of the nanostructured Fe–N–C catalyst for complex synthetic reactions but also provides a new efficient and sustainable method for the synthesis of pharmaceutically important N-heterocycles.

## Conflicts of interest

There are no conflicts to declare.

## Supplementary Material

Supplementary information
