# Correction: Synergistic catalysis on Fe–N_*x*_ sites and Fe nanoparticles for efficient synthesis of quinolines and quinazolinones *via* oxidative coupling of amines and aldehydes

**DOI:** 10.1039/c9sc90241d

**Published:** 2019-11-11

**Authors:** Zhiming Ma, Tao Song, Youzhu Yuan, Yong Yang

**Affiliations:** a Qingdao Institute of Bioenergy and Bioprocess Technology , Chinese Academy of Sciences , Qingdao 266101 , P. R. China; b University of Chinese Academy of Sciences , Beijing , 100049 , P. R. China; c State Key Laboratory of Physical Chemistry of Solid Surface , National Engineering Laboratory for Green Chemical Productions of Alcohols-Ethers-Esters , College of Chemistry and Chemical Engineering , Xiamen University , Xiamen 361005 , P. R. China

## Abstract

Correction for ‘Synergistic catalysis on Fe–N_*x*_ sites and Fe nanoparticles for efficient synthesis of quinolines and quinazolinones *via* oxidative coupling of amines and aldehydes’ by Zhiming Ma *et al.*, *Chem. Sci.*, 2019, DOI: ; 10.1039/c9sc04060a.



## 

The Royal Society of Chemistry regrets that the email addresses for the corresponding authors were not included in the original article.

The email address for Tao Song is Email: songtao@qibebt.ac.cn.

The email address for Yong Yang is Email: yangyong@qibebt.ac.cn.

The Royal Society of Chemistry apologises for these errors and any consequent inconvenience to authors and readers.

