# Supplementary material for: Synergistic catalysis on Fe–Nx sites and Fe nanoparticles for efficient synthesis of quinolines and quinazolinones via oxidative coupling of amines and aldehydes
Source: Chem Sci. 2019 Sep 23;10(44):10283–9. doi: 10.1039/c9sc04060a (PMC6984390; doi:10.1039/c9sc04060a)
Supplement: Supplementary file 1 [file SC-010-C9SC04060A-s001.pdf]

Electronic Supplementary Material (ESI) for Chemical Science.
This journal is © The Royal Society of Chemistry 2019

Supporting Information

Synergistic Catalysis of Fe-N_x Sites and Fe Nanoparticles for Efficient Synthesis of Quinolines and Quinazolinones via Oxidative Coupling of Amines and Aldehydes

Zhiming Ma, Tao Song, Youzhu Yuan, and Yong Yang*

*Corresponding author

E-mail: yangyong@qibebt.ac.cn

1. General considerations

Unless otherwise noted, all reagents were purchased commercially from Sigma-Aldrich, or Aladdin and used as received without further purification. The fresh bamboo shoots were obtained from Anhui Taiping Test Centre, International Centre for Bamboo and Rattan, Anhui Province, China. All operations were carried out in an air atmosphere using glovebox and Schlenk techniques unless otherwise specified. The X-ray diffraction (XRD) patterns of all the catalysts were obtained on a Bruker D8 Advance X-ray diffraction diffractometer equipped with Cu Ka radiation (λ= 1.5147 Å). The morphology of catalysts was examined by an H-7600 transmission electron microscopy (TEM), a Tecnai G2 F30 high-resolution TEM (HRTEM) and a FEI Tecnai G2 F20 scanning transmission electron microscopy (STEM). Nitrogen adsorption–desorption data were obtained on a Micromeritics ASAP 2020 static volumetric sorption analyzer. The specific surface area of the samples was calculated by the Brunauer-Emmet-Teller (BET) method. The micropore volume was calculated by t-plot method. The pore size distributions were determined by non-local density functional theory (NLDFT). The X-ray photoelectron spectroscopy (XPS) data was collected on an ESCALAB 250Xi (Thermo Scientific, UK) instrument equipped with a monochromatized Al Ka line source. All the binding energies obtained were calibrated based on the C 1s peak at 284.8 eV. X-ray absorption spectroscopy (XAS) measurements were carried out at room temperature on the 1W1B beamline at BSRF (Beijing Synchrotron Radiation Facility). Samples were pelletized as disks of 6 mm diameter using paraffin as a binder, while the iron phthalocyanine (FePc) was mixed with BN powder. Transmission-mode Fe K-edge X-ray absorption spectra were collected for all samples over a range of 6974-8110 eV. The monochromator energy was calibrated using a Fe foil. The XAFS data were analyzed using IFEFFIT. The XAFS raw data were background subtracted, normalized and Fourier transformed by standard procedure within the ATHENA program. The elemental composition analysis of the catalysts was conducted on Vario El elemental analyzer. Ion Chromatography was conducted on a Thermo Scientific Dionex ICS-5000 equipped

with CS-12 column with methanesulfonic acid (20 mM) as an eluent. Raman spectra were obtained on a Horiba Jobin Yvon LabRAM HR800 Raman spectrometer system using a 532 nm wavelength laser at room temperature. Inductively coupled plasma atomic emission spectroscopy (ICP-AES) was conducted on a PerkinElmer Optima 5300 DV instrument. Gas chromatography analysis was performed on an Agilent HP-7890 instrument with a flame ionization detector (FID) and an HP-5MS capillary column (30 m, 0.25 mm i.d., 0.25 μm film thicknesses) using helium as the carrier gas. Gas chromatography-mass spectrometry analysis was carried out on an Agilent HP-7890 instrument with an Agilent HP-5975 with triple-axis detector and HP-5 capillary column using helium carrier gas. NMR spectra were from a Bruker DRX-400, or DRX-600, instrument and calibrated using residual non-deuterated solvent (CDCl3: δH = 7.26 ppm, δC = 77.16 ppm; DMSO-d6, δH = 2.50 ppm, δC = 39.60 ppm) as an internal reference.

2. Preparation of catalysts

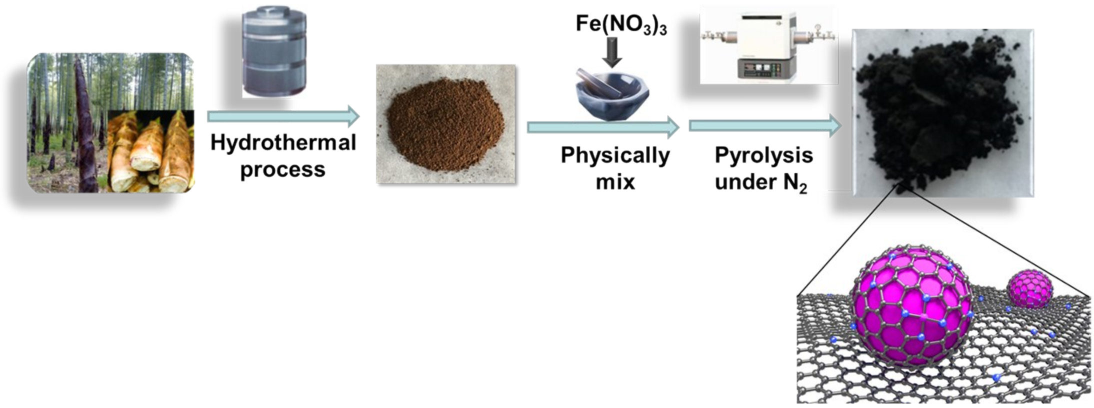

Scheme S1. Schematic Illustration of catalyst preparation.

The catalyst preparation is illustrated as Scheme S1. The hydrochars were firstly prepared by hydrothermal method using bamboo shoots as raw material. The fresh bamboo shoots were cut into slices, dried and ground into a powder. 2 g of the dried bamboo shoots were added to 20 mL of deionized water in a 100 mL Teflon-inner stainless steel autoclave, which was sealed and heated at 180 ^{o}C for 5.5 h. The resulting solids were obtained by filtration, washed thoroughly using distilled water to

remove any soluble metals, and dried by vacuum freeze-drying. After that, 1 g of the obtained hydrochars were mixed with 20 mL of $Fe(NO_3)_3$ aqueous solution (0.4 mmol Fe) and the suspension was stirred at 60 °C for 2 h and then dried at 100 °C for 10 h to remove water. Afterward, the solids were grinded to fine powders and heated to 700, 800 or 900 °C at a rate of 5 °C/min and maintained at this temperature for 2 h under N_2 atmosphere. The obtained catalysts were named as Fe-Fe_3C@NC-T, where T represents the calcination temperature. For comparison, the catalyst Fe-Fe_3C@C-800 was prepared by pyrolysis of a mixture of $Fe(NO_3)_3$ with commercially available activated carbon without N-doping, and the bare support NC without metal loading was also prepared by a similar procedure, respectively.

3. Characterization results

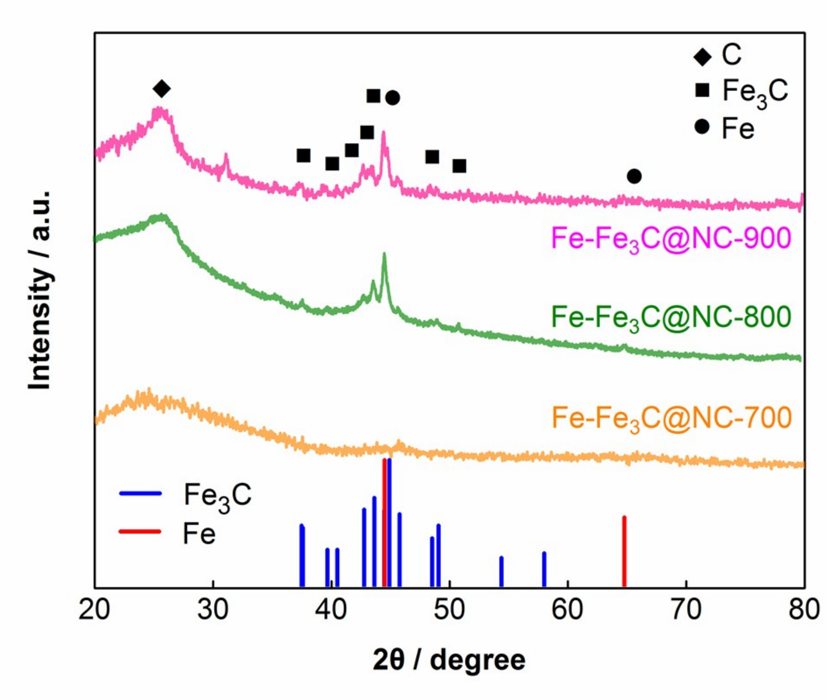

Figure S1. XRD patterns for the catalysts Fe-Fe_3C@NC-700, Fe-Fe_3C@NC-800, and Fe-Fe_3C@NC-900.

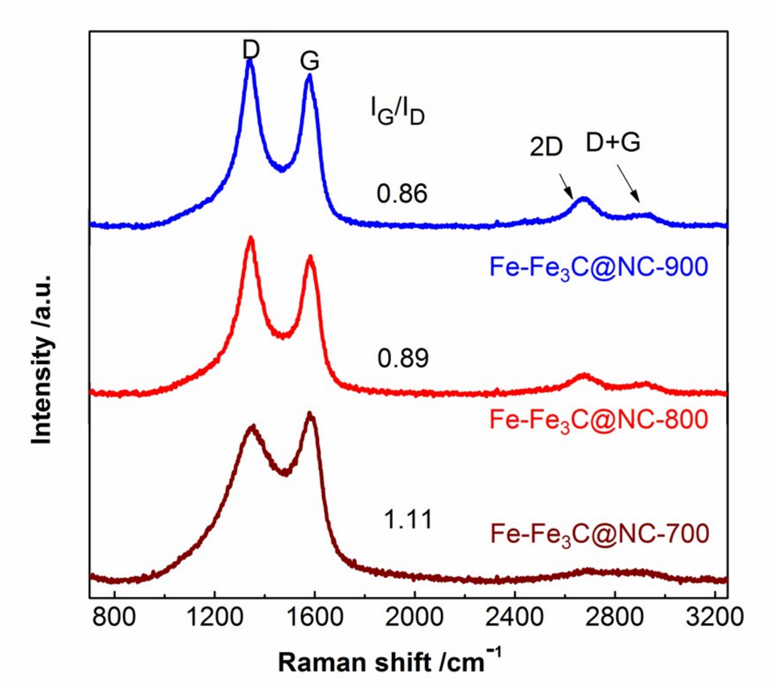

Figure S2. Raman spectrum of the catalysts Fe-Fe$_3$C@NC-700, Fe-Fe$_3$C@NC-800, and Fe-Fe$_3$C@NC-900.

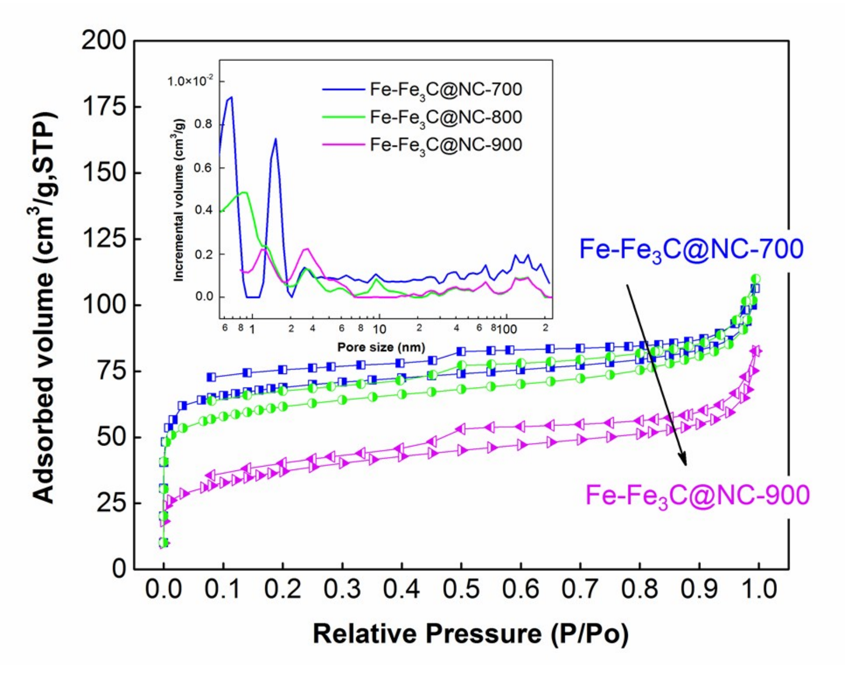

Figure S3. N_2 sorption isotherms and pore size distribution calculated using a nonlocal density functional theory (NLDFT) method for the catalysts Fe-Fe$_3$C@NC-700, Fe-Fe$_3$C@NC-800, and Fe-Fe$_3$C@NC-900.

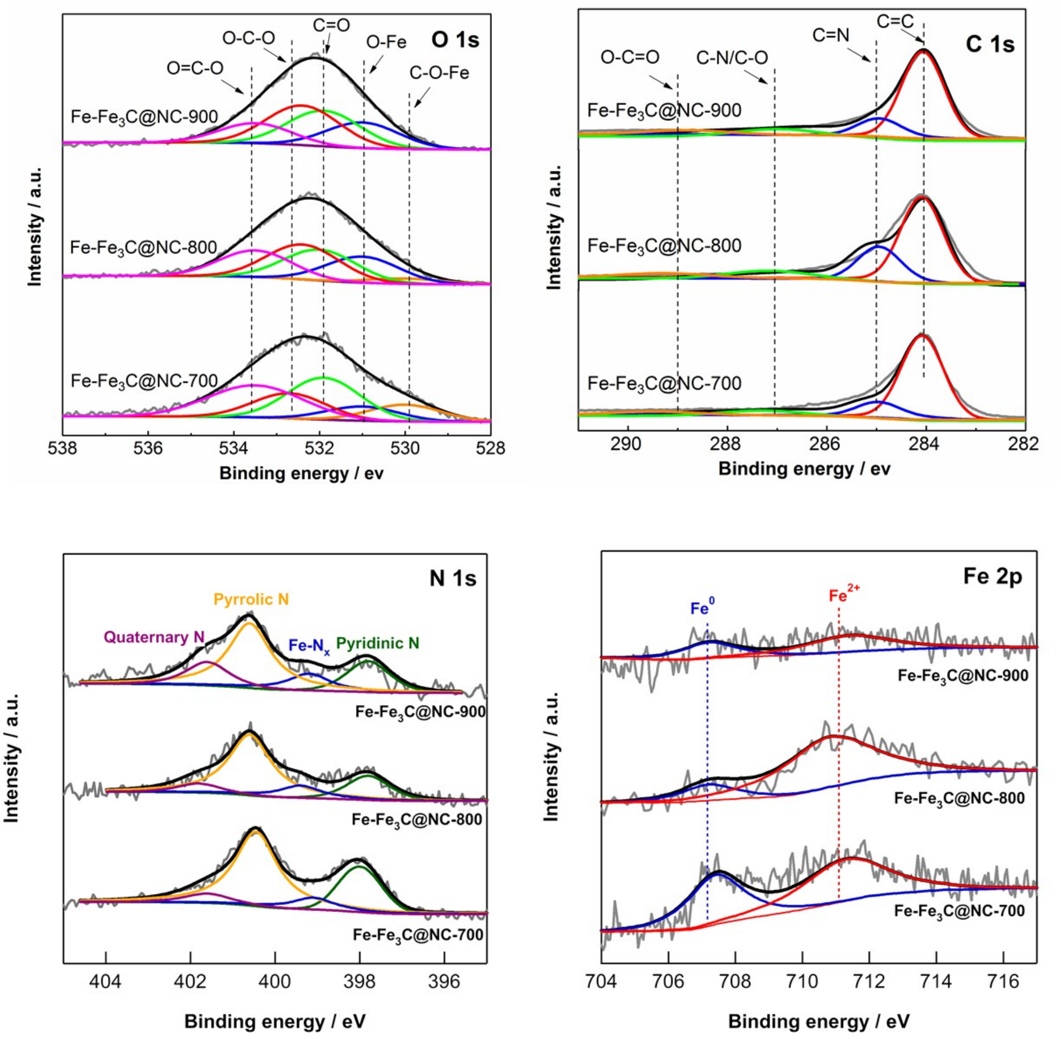

Figure S4. O 1s, C 1s, N 1s and Fe 2p XPS spectra for the catalysts Fe-Fe_3C@NC-700, Fe-Fe_3C@NC-800, and Fe-Fe_3C@NC-900.

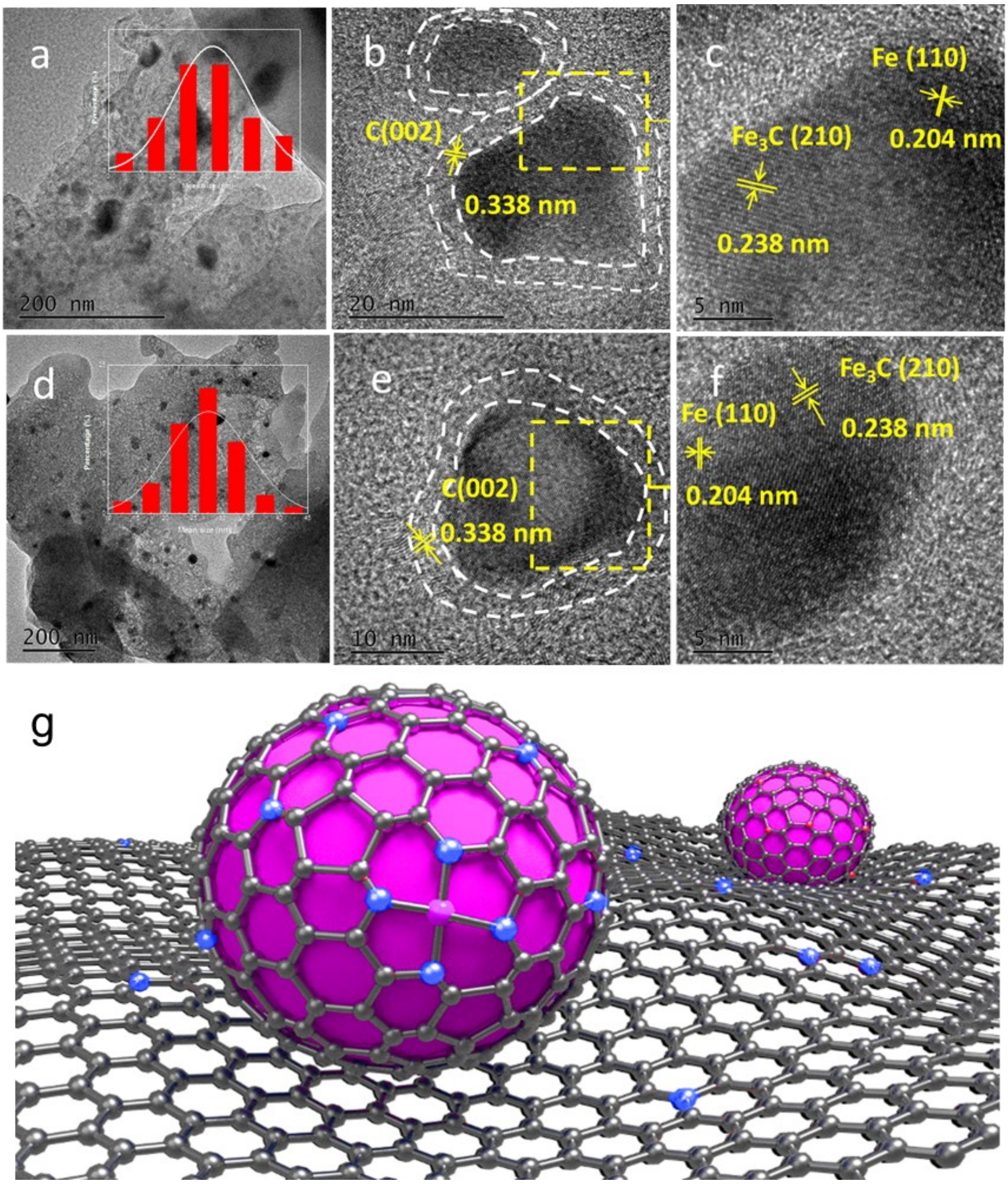

Figure S5. (a) TEM and (b & c) HR-TEM images of the catalyst Fe-Fe_3C@NC-700, the inset is the size distribution of metallic Fe nanoparticles, (d) TEM and (e & f) HR-TEM images of the catalyst Fe-Fe_3C@NC-900, the inset is the size distribution of metallic Fe nanoparticles. (g) Schematic illustration of the catalyst Fe-Fe_3C@NC-T.

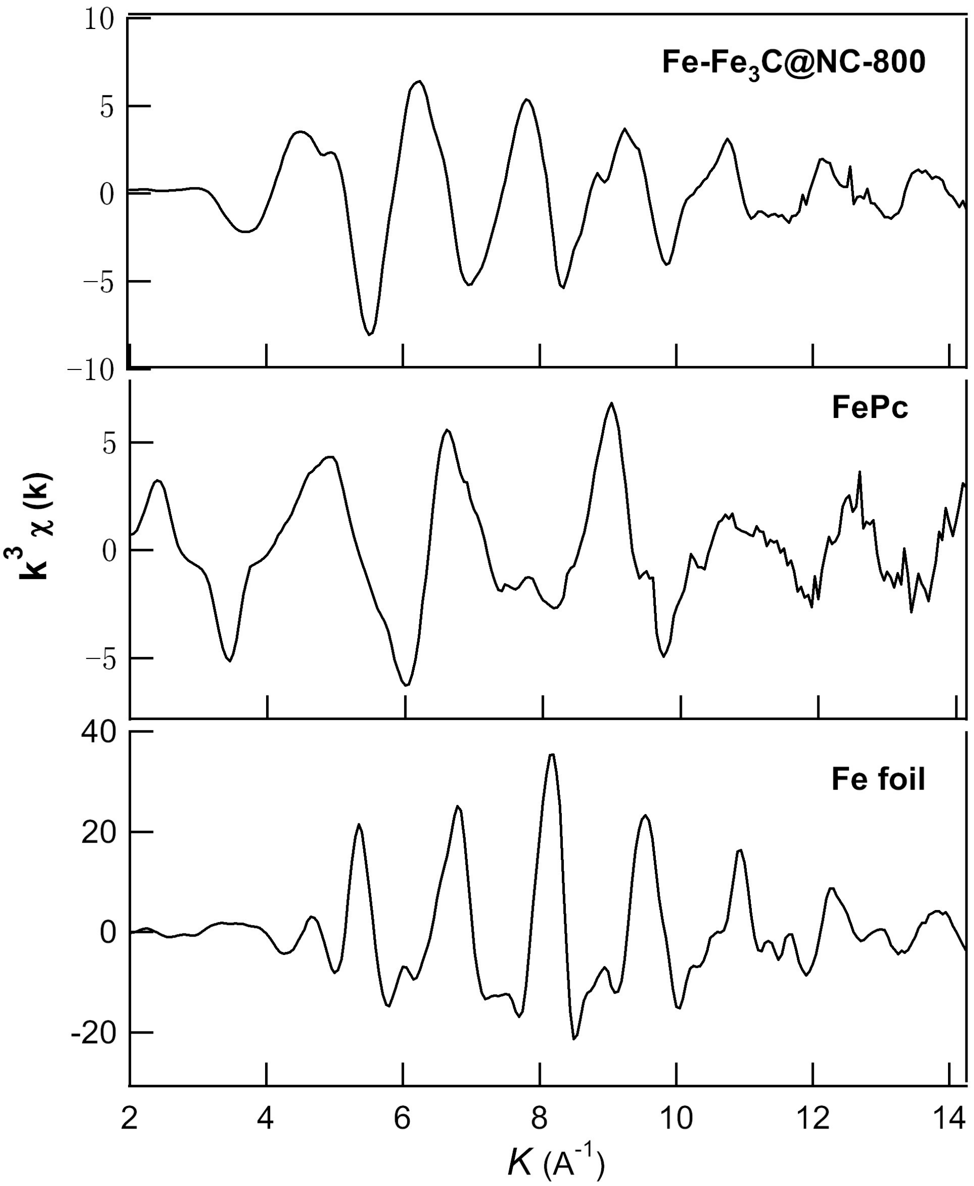

Figure S6. Fe K-edge EXAFS oscillations of Fe foil, FePc and Fe-Fe3C@NC-800 at k-space.

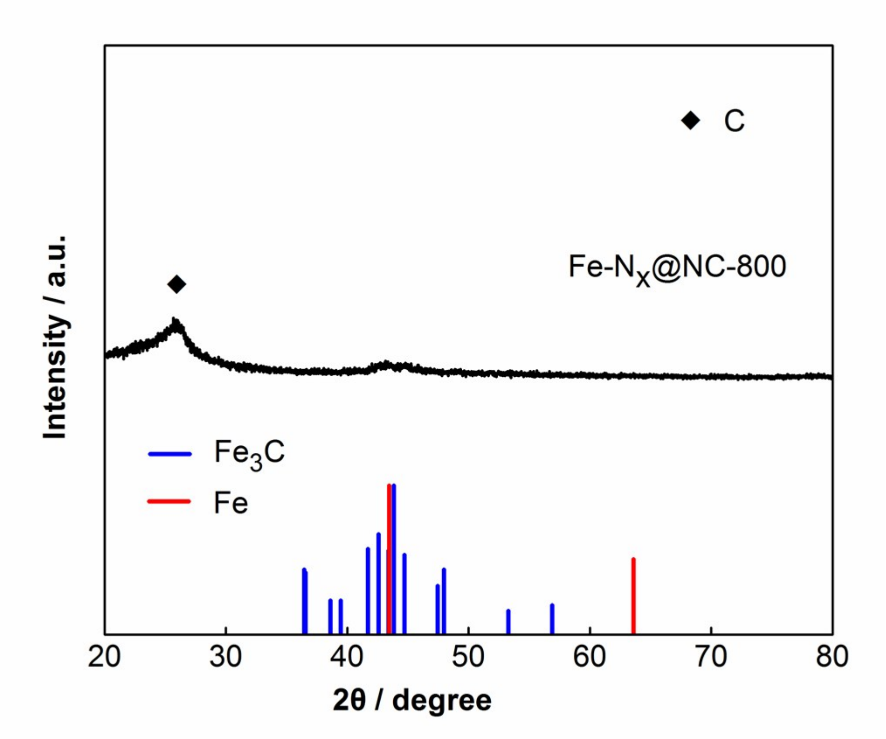

Figure S7. XRD patterns for the catalyst Fe-N_x@NC-800 after acid-etching (The catalyst Fe-Fe_3C@NC-800 was leached in 0.5 M H_2SO_4 at 60 °C for 12 h to remove or substantially diminish metallic Fe nanoparticles, denoted as Fe-Nx@NC-800).

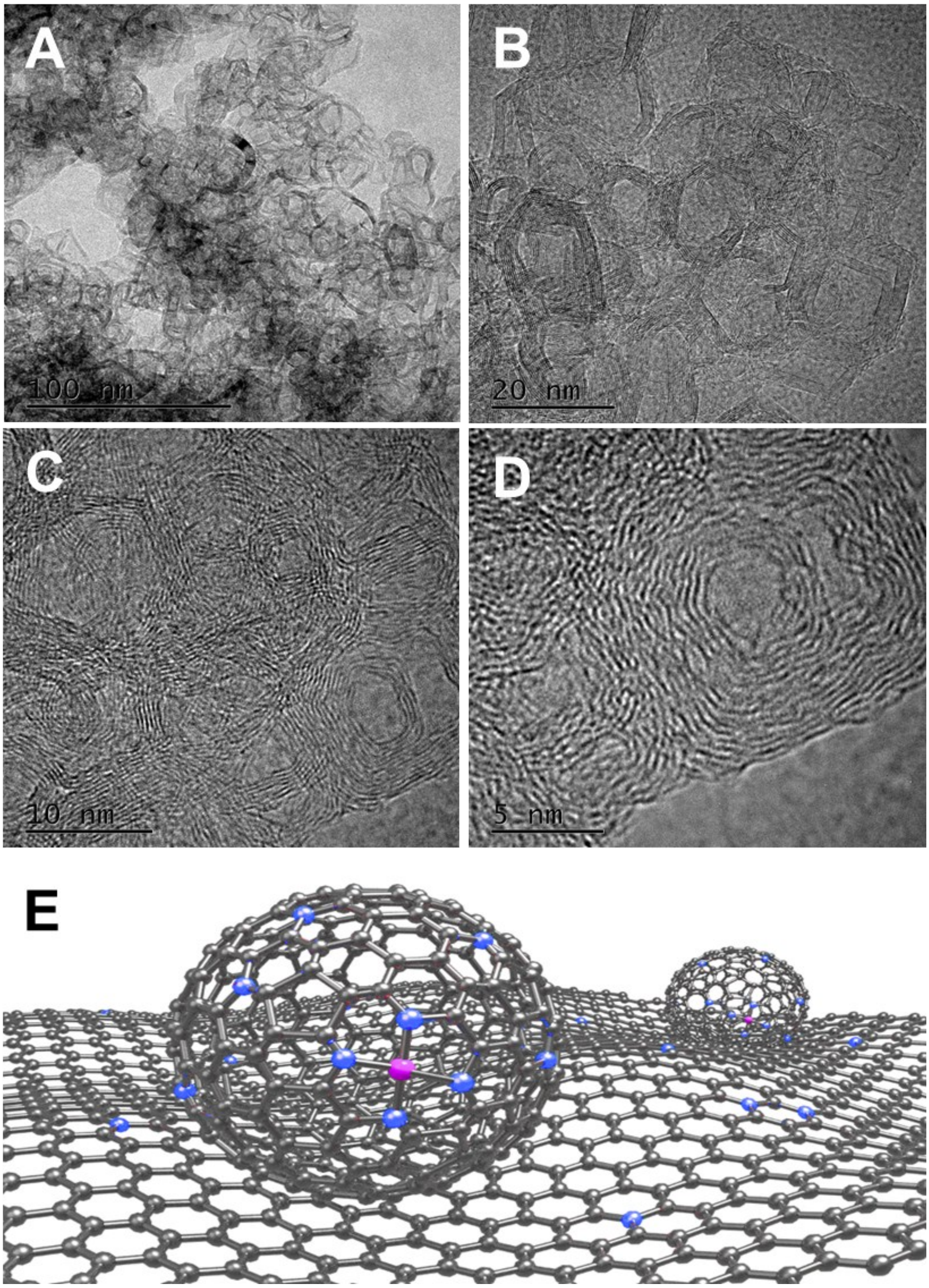

Figure S8. (A-D) TEM and HR-TEM images of the catalyst Fe-N_x@NC-800 after acid-etching and (E) Schematic illustration of the catalyst Fe-N_x@NC-800 (The catalyst Fe-Fe_3C@NC-800 was leached in 0.5 M H_2SO_4 at 60 °C for 12 h to remove or substantially diminish metallic Fe nanoparticles, denoted as Fe-N_x@NC-800).

Table S1. Chemical composition and texture properties of catalysts Fe-Fe_3C@NC-T and acid-etched catalyst Fe-N_x@NC-800.

Sample	Fe content[a] (wt%)	Elemental analysis		BET analysis	
		C (wt%)	N (wt%)	S_{BET}[b] (m^2g^{-1})	Pore volume (cm^3g^{-1})
Fe-Fe_3C@NC-700	4.29	69.56	4.05	257.7	0.164
Fe-Fe_3C@NC-800	4.38	67.96	2.22	226.1	0.170
Fe-Fe_3C@NC-900	4.51	65.32	2.14	129.5	0.128
Fe-N_x@NC-800	1.42	-	-	-	-

[a]Determined by ICP-OES. [b]Specific surface areas were determined by the BET multipoint method.

4. General procedure for synthesis of N-heterocycles

4.1. General procedure for synthesis of Quinolines

A 25 mL sealing tube was charged with a magnetic stirring, aldehydes (0.2 mmol), amines (0.24 mmol), Fe-Fe_3C@C-800 (10 mg, 4 mol% of Fe), H_2O_2 (0.4 mmol), H_2O /THF (5 mL, v/v = 4/1). The reaction was stirred for 12 h at 100°C under atmospheric air. After completion of the reaction, the reaction mixture was cooled to room temperature and the liquid was extracted by ethyl acetate and the organic phase was analyzed by GC and GC-MS to determine the conversion and yield using 1,3,5-trimethyl-benzen as an internal standard. The products were purified by column chromatography and structurally confirmed by NMR.

4.2. General procedure for synthesis of Quinazolinones

A 25 mL sealing tube was charged with a magnetic stirring, aldehydes (0.2 mmol), amides (0.24 mmol), Fe-Fe_3C@C-800 (10 mg, 4 mol% of Fe), H_2O_2 (0.8 mmol), H_2O /THF (4/1 v/v) 5 mL. The reaction was stirred for 12 h at 100°C under atmospheric air.

After completion of the reaction, the reaction mixture was cooled to room temperature and the liquid was extracted by ethyl acetate and the organic phase was analyzed by GC and GC-MS to determine the conversion and yield using 1,3,5-trimethyl-benzen as an internal standard. The products were purified by column chromatography and structurally confirmed by NMR.

5. Catalytic studies

5.1. Conditions optimization

Table S2. The influence of the amount of H_2O_2 on synthesis of 2-phenylquinazoline.[a]

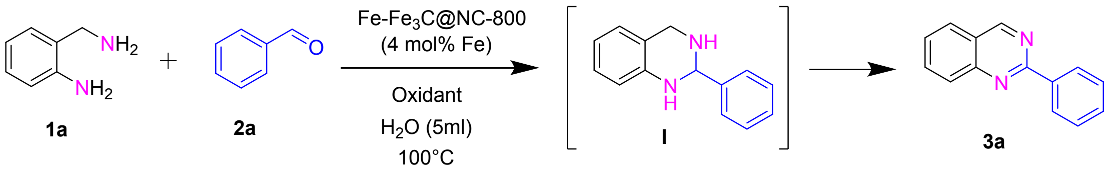

Entry	Oxidant	Conversion (%)[b]	GC yield (%)[b]	
			**I**	**3a**
1	-	95	88	7
2	H_2O_2 (2.0eq)	96	18	78
3	H_2O_2 (3.0eq)	93	23	70
4	H_2O_2 (5.0eq)	87	21	66

[a]Reaction conditions: 2-aminobenzylamine (**1a**) (0.2 mmol), benzyl aldehyde (**2a**) (0.24 mmol), Fe-Fe_3C@NC-800 (10 mg, 4 mol% of Fe), H_2O_2 (with respect to **1a**, 30% in H_2O), H_2O (5 ml), under atmospheric air, 100°C, 12 h. [b]Determined by GC and GC-MS using 1,3,5-trimethyl-benzen as an internal standard sample and confirmed with their corresponding authentic samples.

Table S3. The influence of the amount of benzaldehyde on synthesis of 2-phenylquinazoline.[a]

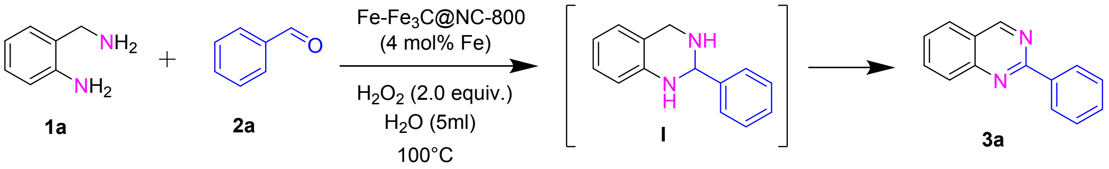

Entry	2a	Conversion (%)[b]	GC yield (%)[b]	
			**I**	**3a**
1	1.0 eq	85	18	67
2	1.2 eq	96	18	78
3	1.5 eq	80	17	63
4	2 eq	73	28	45

[a]Reaction conditions: 2-aminobenzylamine (**1a**) (0.2 mmol), benzyl aldehyde (**2a**) (with respect to **1a**), Fe-Fe_3C@NC-800 (10 mg, 4 mol% of Fe), H_2O_2 (2 equivalent with respect to **1a**, 30% in H_2O), H_2O (5 ml), under atmospheric air, 100°C, 12 h. [b]Determined by GC and GC-MS using 1,3,5-trimethyl-benzen as an internal standard sample and confirmed with their corresponding authentic samples.

Table S4. The influence of the solvent on synthesis of 2-phenylquinazoline.[a]

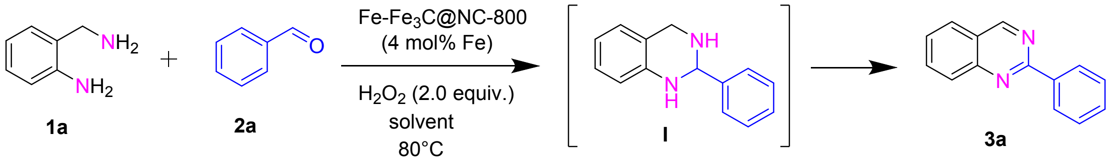

Entry	solvent (mL)	Conversion (%)[b]	GC yield (%)[b]	
			**I**	**3a**
1	H_2O-CH_3CN(4:1)	87	32	55
2	H_2O-EtOH(4:1)	83	40	43
3	H_2O-THF(4:1)	90	24	66
4[c]	H_2O-THF(4:1)	100	5	95
5[c]	H_2O-THF(3:2)	87	14	73

[a]Reaction conditions: 2-aminobenzylamine (**1a**) (0.2 mmol), benzyl aldehyde (**2a**) (0.24 mmol), Fe-Fe_3C@NC-800 (10 mg, 4 mol% of Fe), H_2O_2 (2 equivalent with respect to **1a**, 30% in H_2O), solvent (5 ml), under atmospheric air, 80 °C. 12 h. [b]Determined by GC and GC-MS using 1,3,5-trimethyl-benzen as an internal standard sample and confirmed with their corresponding authentic samples. [c]100 °C

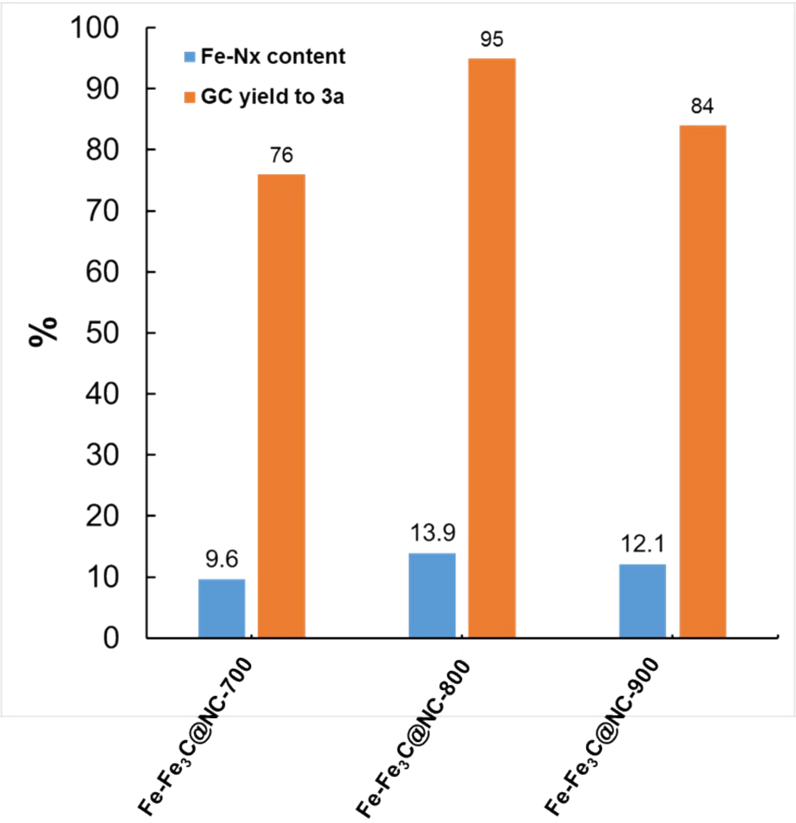

Figure S9. Effect of the content of Fe-N_x sites in the catalysts Fe-Fe_3C@NC-700, 800 and 900 for the benchmark reaction under standard conditions.

5.2 Recyclability of catalyst

The synthesis of 2-phenylquinazoline was chosen as the model reaction to investigate the recyclability of the catalyst Fe-Fe_3C@NC-800. A 25 mL sealing tube was charged with a magnetic stirring bar, 2-aminobenzylamine (**1a**) (0.2 mmol), benzyl aldehyde (**2a**) (0.24 mmol), Fe-Fe_3C@NC-800 (10 mg, 4 mol% of Fe), H_2O_2 (2 equivalent with respect to **1a**, 30% in H_2O), H_2O-THF (5 ml, 4/1, v/v). The reaction was stirred for 12 h at 100 °C under atmospheric air. After completion of the reaction, the reaction mixture was cooled to room temperature and the conversion and yield was determined by GC and GC-MS using 1,3,5-trimethyl-benzen as an internal standard sample and confirmed with their corresponding authentic samples. The residue was dispersed in 5 mL of ethanol/H_2O (v/v=4/1) and the resulting mixture was stirred for 10 min, the catalyst were separated by centrifuge. Such operation was repeated for 3 times. Finally, the obtained black solid was dried under vacuum at 40°C overnight for

successive use.

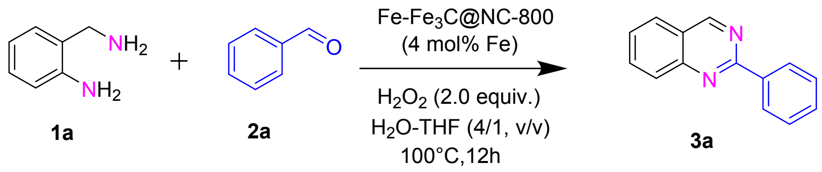

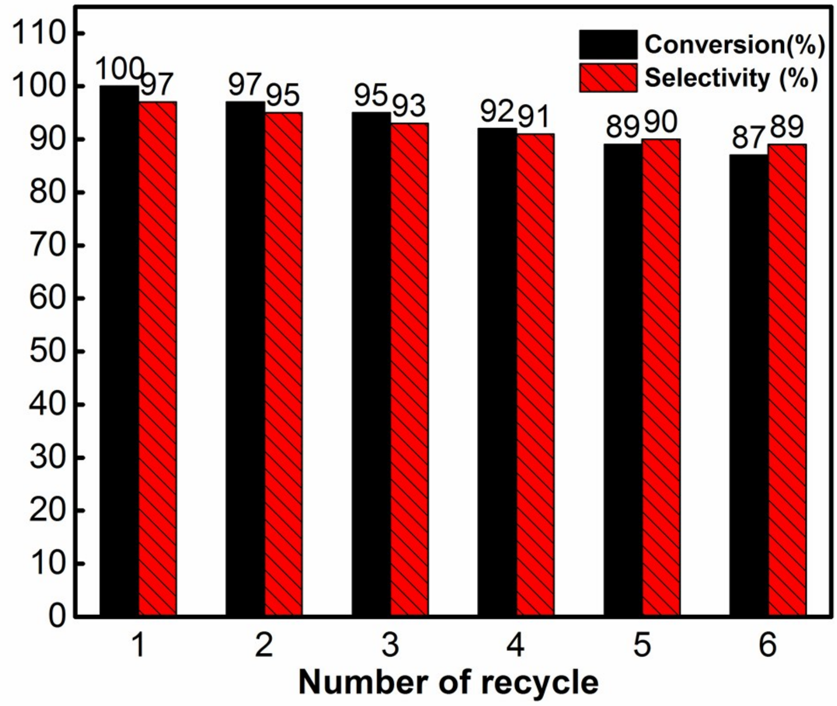

Figure S10. Recyclability of the catalyst Fe-Fe_3C@NC-800 for the synthesis of 2-phenylquinazoline under the standard conditions.

^{1}H and ^{13}C NMR Characterization of products

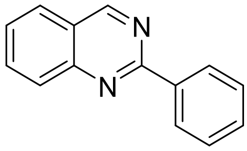

2-phenylquinazoline (3a), White solid; ^{1}H NMR (400 MHz, $CDCl_3$): δ 9.48 (s, 1H), 8.67-8.57 (m, 2H), 8.11 (dd, *J* = 8.4, 1.0 Hz, 1H), 7.98-7.87 (m, 2H), 7.68-7.45 (m, 4H). ^{13}C NMR (101 MHz, $CDCl_3$): δ 161.1, 160.5, 150.8, 149.4, 138.1, 134.2, 130.7, 128.7, 128.6, 127.3, 127.2, 123.6. The spectroscopic data matched that previously report.[1]

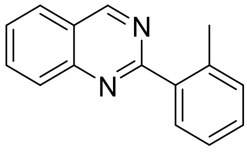

2-(o-tolyl)quinazoline (3b), Yellow solid; ^{1}H NMR (400 MHz, $CDCl_3$): δ 9.51 (d, *J* = 0.8 Hz, 1H), 8.10 (dd, *J* = 8.5, 1.0 Hz, 1H), 8.01-7.87 (m, 3H), 7.67 (s, 1H), 7.42-7.30 (m, 3H), 2.61 (s, 3H). ^{13}C NMR (101 MHz, $CDCl_3$): δ 164.1, 160.1, 150.4, 138.6, 137.4, 134.1, 131.3, 130.6, 129.3, 128.6, 127.5, 127.1, 125.9, 122.9, 21.03. The spectroscopic data matched that previously report.[1]

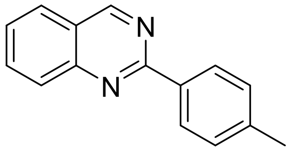

2-(p-tolyl)quinazoline (3c), White solid; ^{1}H NMR (400 MHz, $CDCl_3$): δ 9.45 (s, 1H), 8.51 (d, *J* = 8.0 Hz, 2H), 8.08 (d, *J* = 8.4 Hz, 1H), 7.96-7.85 (m, 2H), 7.60 (ddd, *J* = 8.1, 6.9, 1.1 Hz, 1H), 7.35 (d, *J* = 7.9 Hz, 2H), 2.45 (s, 3H). ^{13}C NMR (101 MHz, $CDCl_3$): δ 161.1, 160.46, 150.76, 140.95, 135.22, 134.12, 129.43, 128.56, 128.53, 127.10, 127.08, 123.52, 21.53. The spectroscopic data matched that previously report.[1]

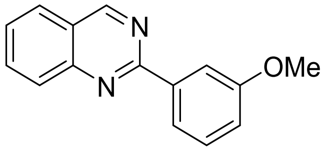

2-(3-methoxyphenyl)quinazoline (3d), White solid; ^{1}H NMR (400 MHz, $CDCl_3$): δ 9.47 (d, *J* = 1.0 Hz, 1H), 8.28-8.15 (m, 2H), 8.10 (d, *J* = 8.5 Hz, 1H), 7.97-7.87 (m,

2H), 7.62 (d, *J* = 1.1 Hz, 1H), 7.45 (s, 1H), 7.08 (d, *J* = 2.7 Hz, 1H), 3.96 (s, 3H). ^{13}C NMR (101 MHz, $CDCl_3$): δ 160.9, 160.5, 160.0, 150.8, 139.5, 134.1, 129.7, 128.7, 127.3, 127.1, 123.8, 121.2, 117.3, 113.0, 55.5. The spectroscopic data matched that previously report.[1]

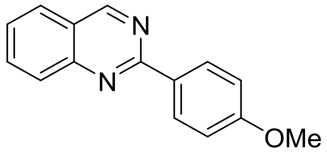

2-(4-methoxyphenyl)quinazoline (3e), faint yellow solid; ^1H NMR (400 MHz, $CDCl_3$): δ 9.42 (s, 1H), 8.58 (d, *J* = 8.9 Hz, 2H), 8.04 (dd, *J* = 8.4, 1.0 Hz, 1H), 7.93-7.82 (m, 2H), 7.57 (ddd, *J* = 8.1, 6.9, 1.1 Hz, 1H), 7.10-7.00 (m, 2H), 3.90 (s, 3H). ^{13}C NMR (101 MHz, $CDCl_3$): δ 161.8, 160.9, 160.4, 150.9, 134.0, 130.7, 130.2, 128.43 127.1, 126.8, 123.3, 113.9, 55.4. The spectroscopic data matched that previously report.[1]

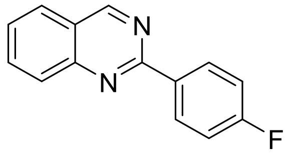

2-(4-fluorophenyl)quinazoline (3f), White solid; ^1H NMR (400 MHz, $CDCl_3$): δ 9.44 (s, 1H), 8.63 (dd, *J* = 8.8, 5.7 Hz, 2H), 8.06 (dd, *J* = 8.3, 1.0 Hz, 1H), 7.92 (dd, *J* = 7.5, 1.4 Hz, 2H), 7.65-7.56 (m, 1H), 7.21 (t, *J* = 8.7 Hz, 2H). ^{13}C NMR (101 MHz, $CDCl_3$): δ 164.6 (d, *J* = 248.3 Hz), 160.5, 160.1, 150.7, 134.2, 130.8, 130.6, 128.5, 127.3, 127.1, 123.5, 115.6 (d, *J* = 21.1 Hz). The spectroscopic data matched that previously report.[1]

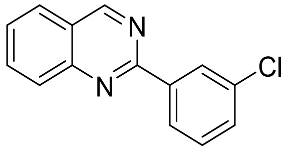

2-(3-chlorophenyl)quinazoline (3g), White solid; ^1H NMR (400 MHz, $CDCl_3$): δ 9.50 (d, *J* = 0.9 Hz, 1H), 8.98 (d, *J* = 1.8 Hz, 1H), 8.88 (dt, *J* = 8.0, 1.5 Hz, 1H), 8.12 (dd, *J* = 8.3, 1.0 Hz, 1H), 8.01-7.93 (m, 2H), 7.82-7.74 (m, 1H), 7.73-7.60 (m, 2H). ^{13}C NMR (101 MHz, $CDCl_3$) δ 165.9, 163.4, 160.5, 160.1, 150.7, 134.3 130.7, 130.6, 128.6, 127.3 127.2 123.5, 115.7, 115.5. The spectroscopic data matched that previously report.[1]

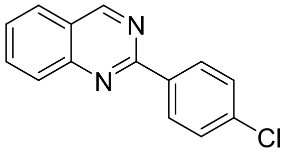

2-(4-chlorophenyl)quinazoline (3h), White solid; ^{1}H NMR (400 MHz, $CDCl_3$): δ 9.46 (s, 1H), 8.62-8.53 (m, 2H), 8.09 (d, *J* = 8.4 Hz, 1H), 7.93 (ddd, *J* = 9.9, 8.1, 1.9 Hz, 2H), 7.68-7.59 (m, 1H), 7.54-7.45 (m, 2H). ^{13}C NMR (101 MHz, $CDCl_3$): δ 160.6, 160.0, 150.7, 136.9, 136.5, 134.3, 129.9, 128.8, 128.6, 127.5, 127.2, 123.6. The spectroscopic data matched that previously report.[1]

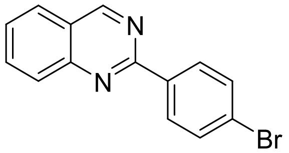

2-(4-bromophenyl)quinazoline (3i), White solid; ^{1}H NMR (400 MHz, $CDCl_3$): δ 9.51-9.40 (m, 1H), 8.56-8.46 (m, 2H), 8.08 (dd, *J* = 8.3, 1.0 Hz, 1H), 7.98-7.87 (m, 2H), 7.65 (dd, *J* = 8.3, 6.6 Hz, 3H). ^{13}C NMR (101 MHz, $CDCl_3$): δ 159.5, 159.2, 149.7, 135.9, 133.3, 130.8, 129.1, 127.6, 127.5, 126.5, 126.1, 124.4. The spectroscopic data matched that previously report.[1]

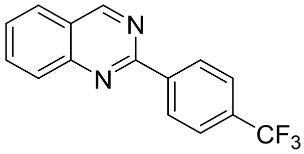

2-(4-(trifluoromethyl)phenyl)quinazoline (3j), White solid; ^{1}H NMR (400 MHz, $CDCl_3$): δ 9.49 (s, 1H), 8.75 (d, *J* = 8.1 Hz, 2H), 8.11 (d, *J* = 8.4 Hz, 1H), 7.96 (d, *J* = 8.8 Hz, 2H), 7.79 (d, *J* = 8.2 Hz, 2H), 7.68 (d, *J* = 7.5 Hz, 1H). ^{13}C NMR (101 MHz, $CDCl_3$): δ 160.6, 159.6, 150.7, 141.3, 134.4, 132.6, 132.3 (q, $J_{C\text{-}F}$ = 32.2 Hz), 128.8, 128.7, 127.9, 127.2, 125.5 (q, $J_{C\text{-}F}$ = 3.8 Hz), 123.85. The spectroscopic data matched that previously report.[1]

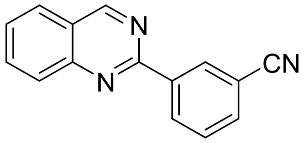

3-(quinazolin-2-yl)benzonitrile (3k), White solid; ^{1}H NMR (400 MHz, $CDCl_3$): δ 9.48 (d, *J* = 3.0 Hz, 1H), 8.65 (d, *J* = 2.8 Hz, 1H), 8.52 (dd, *J* = 5.0, 2.5 Hz, 1H), 8.11 (dd, *J* = 8.5, 2.9 Hz, 1H), 8.00 – 7.88 (m, 2H), 7.66 (d, *J* = 2.8 Hz, 1H), 7.55 – 7.41

(m, 2H). 13C NMR (101 MHz, $CDCl_3$): δ = 160.59, 158.20, 152.16, 150.28, 138.36, 134.81, 134.33, 130.56, 129.87, 128.71, 127.67, 126.65, 117.22, 116.72, 111.80. The spectroscopic data matched that previously report.[2]

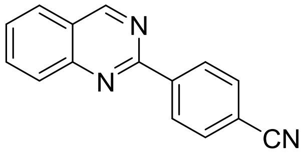

4-(quinolin-2-yl)benzonitrile (3l), White solid; ^1H NMR (400 MHz, $CDCl_3$): δ 9.54 – 9.47 (m, 1H), 8.82 – 8.70 (m, 2H), 8.12 (dt, J = 8.4, 1.0 Hz, 1H), 7.97 (ddd, J = 9.7, 8.0, 1.6 Hz, 2H), 7.89 – 7.79 (m, 2H), 7.69 (ddd, J = 8.2, 7.0, 1.1 Hz, 1H). ^{13}C NMR (101 MHz, $CDCl_3$): δ = 160.71, 159.10, 150.61, 134.59, 132.43, 129.91, 129.04, 128.81, 128.21, 127.24, 123.89, 118.92, 113.83. The spectroscopic data matched that previously report.[2]

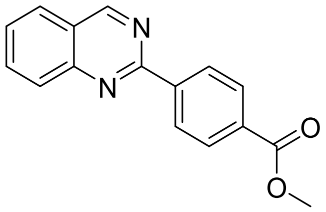

Methyl 4-(quinazolin-2-yl)benzoate (3m), yellow oil; ^1H NMR (400 MHz, $CDCl_3$): δ 9.50 (s, 1H), 8.70 (d, J = 8.1 Hz, 2H), 8.16 (dd, J = 32.3, 8.3 Hz, 3H), 8.03-7.85 (m, 2H), 7.66 (t, J = 7.5 Hz, 1H), 3.97 (s, 3H). ^{13}C NMR (101 MHz, $CDCl_3$): δ 166.9, 160.6, 160.0, 150.7, 142.1, 134.4, 131.7, 129.9, 128.8, 128.5, 127.8, 127.2, 123.8, 52.26. The spectroscopic data matched that previously report.[3]

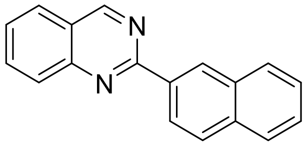

2-(naphthalen-2-yl)quinazoline (3n), White solid; ^1H NMR (400 MHz, $CDCl_3$): δ 9.53 (s, 1H), 9.17 (s, 1H), 8.74 (dd, J = 8.7, 1.7 Hz, 1H), 8.15 (dd, J = 8.4, 1.0 Hz, 1H), 8.09-7.84 (m, 5H), 7.70-7.60 (m, 1H), 7.59-7.46 (m, 2H). ^{13}C NMR (101 MHz, $CDCl_3$): δ 161.0, 160.5, 150.9, 135.4, 134.7, 134.2, 133.4, 129.3, 128.9, 128.7, 128.3, 127.7, 127.3, 127.2, 127.1, 126.2, 125.4, 123.7. The spectroscopic data matched that previously report.[3]

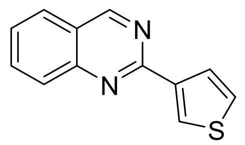

2-(thiophen-3-yl)quinazoline (3o), Brown solid; ^{1}H NMR (400 MHz, $CDCl_3$): δ 9.48 (s, 1H), 8.70-8.55 (m, 1H), 8.10 (d, J = 8.4 Hz, 1H), 8.02-7.84 (m, 2H), 7.70-7.44 (m, 3H). ^{13}C NMR (101 MHz, $CDCl_3$): δ 161.1, 160.5, 150.8, 138.1, 134.1, 130.6, 128.8, 128.7, 128.6, 127.3, 127.1, 123.6. The spectroscopic data matched that previously report.[3]

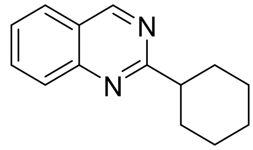

2-cyclohexylquinazoline (3p), White solid; ^{1}H NMR (400 MHz, $CDCl_3$): δ 9.35 (s, 1H), 8.04-7.92 (m, 1H), 7.86 (dd, J = 8.8, 6.6 Hz, 2H), 7.57 (td, J = 7.4, 6.8, 1.1 Hz, 1H), 3.05 (tt, J = 11.8, 3.5 Hz, 1H), 2.08 (ddq, J = 12.4, 3.9, 2.1 Hz, 2H), 1.90 -1.69 (m, 5H), 1.50-1.27 (m, 3H). ^{13}C NMR (101 MHz, $CDCl_3$): δ 170.9, 160.4, 150.4, 133.8, 128.0, 127.0, 126.8, 123.3, 47.9, 31.9, 26.3, 26.1. The spectroscopic data matched that previously report.[5]

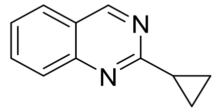

2-cyclopropylquinazoline (3q), White solid; ^{1}H NMR (600 MHz, $CDCl_3$): δ 9.20 (d, J = 2.8 Hz, 1H), 7.94-7.71 (m, 3H), 7.58-7.42 (m, 1H), 2.38 (tt, J = 8.1, 4.7 Hz, 1H), 1.25 (dt, J = 6.6, 3.4 Hz, 2H), 1.12 (dt, J = 8.2, 3.3 Hz, 2H). ^{13}C NMR (151 MHz, $CDCl_3$): δ 171.1, 168.4, 160.3, 150.4, 133.9, 127.5, 127.1, 126.3, 123.2, 18.6, 10.6. The spectroscopic data matched that previously report.[4]

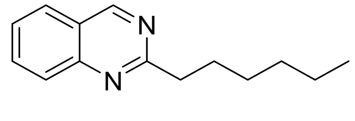

2-hexylquinazoline (3r), White solid; ^{1}H NMR (600 MHz, $CDCl_3$): δ 9.34 (s, 1H), 7.92 (dd, J = 36.1, 8.1 Hz, 3H), 7.59 (d, J = 7.5 Hz, 1H), 3.11 (t, J = 7.8 Hz, 2H), 1.92 (d, J = 7.8 Hz, 2H), 1.47-1.22 (m, 7H), 0.87 (t, J = 6.8 Hz, 3H). ^{13}C NMR (151 MHz, $CDCl_3$): δ 167.9, 160.4, 150.4, 133.9, 127.9, 127.1, 126.9, 123.1, 40.1, 31.7, 29.3,

29.0, 22.6, 14.1. The spectroscopic data matched that previously report.[1]

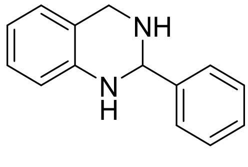

2-phenyl-1,2,3,4-tetrahydroquinazoline, Brown solid; ^{1}H NMR (400 MHz, $CDCl_3$): δ 7.60-7.30 (m, 5H), 7.06 (t, *J* = 7.8 Hz, 1H), 6.96 (d, *J* = 7.5 Hz, 1H), 6.74 (t, *J* = 7.4 Hz, 1H), 6.59 (d, *J* = 8.0 Hz, 1H), 5.25 (d, *J* = 2.0 Hz, 1H), 4.14 (dd, *J* = 111.5, 16.6 Hz, 2H). ^{13}C (101 MHz, $CDCl_3$): δ 142.7, 140.5, 127.7, 127.5, 126.3, 125.6, 125.2, 120.2, 117.1, 113.9, 68.6, 45.4. The spectroscopic data matched that previously report.[1]

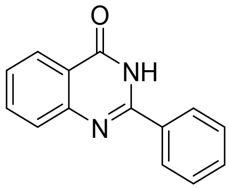

2-phenylquinazolin-4(3H)-one (5a), white solid; ^{1}H NMR (400 MHz, DMSO-d_6) δ 12.58 (brs, 1H), 8.21 (t, *J* = 8.7 Hz, 3H), 7.86 (t, *J* = 7.2 Hz, 1H), 7.78 (d, *J* = 8.0 Hz, 1H), 7.68-7.42 (m, 4H). ^{13}C NMR (101 MHz, DMSO-d_6): δ 162.7, 152.8, 149.2, 135.1, 133.2, 131.8, 129.1, 128.2, 127.9, 127.0, 126.3, 121.5. The spectroscopic data matched that previously report.[7]

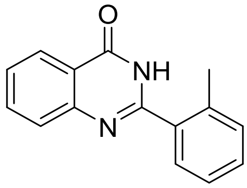

2-(o-tolyl)quinazolin-4(3H)-one (5b), yellow solid; ^{1}H NMR (400 MHz, DMSO-d_6): δ 12.50 (brs, 1H), 8.22 (dd, *J* = 7.9, 1.1 Hz, 1H), 7.95-7.84 (m, 1H), 7.74 (d, *J* = 8.0 Hz, 1H), 7.62-7.49 (m, 2H), 7.48 (td, *J* = 7.5, 1.2 Hz, 1H), 7.44-7.27 (m, 2H), 2.43 (s, 3H). ^{13}C NMR (101 MHz, DMSO-d_6): δ 162.3, 154.8, 149.2, 136.6, 134.9, 134.7, 131.0, 130.4, 129.6, 127.8, 127.1, 126.3, 126.2, 121.5, 20.0. The spectroscopic data matched that previously report.[7]

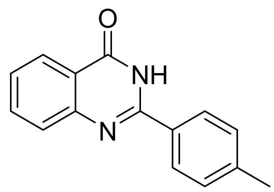

2-(p-tolyl)quinazolin-4(3H)-one (5c), yellow solid; ^{1}H NMR (400 MHz, DMSO-d_6): δ 12.51 (brs, 1H), 8.18 (dd, *J* = 7.9, 0.9 Hz, 1H), 8.13 (d, *J* = 8.2 Hz, 2H), 7.92-7.83 (m, 1H), 7.76 (d, *J* = 8.0 Hz, 1H), 7.54 (dd, *J* = 11.0, 3.9 Hz, 1H), 7.38 (d, *J* = 8.1 Hz, 2H), 2.42 (s, 3H). ^{13}C NMR (101 MHz, DMSO-d_6): δ 162.8, 152.7, 149.3, 141.9, 135.0, 130.4, 129.6, 128.1, 127.9, 126.8, 126.3, 121.4. The spectroscopic data matched that previously report.[7]

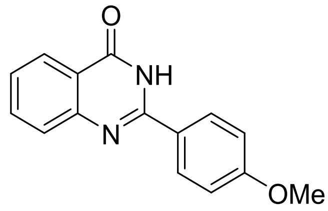

2-(4-methoxyphenyl)quinazolin-4(3H)-one (5d), yellow solid; ^{1}H NMR (400 MHz, DMSO-d_6): δ 12.46 (brs, 1H), 8.29-8.21 (m, 2H), 8.18 (dd, *J* = 7.9, 1.2 Hz, 1H), 7.86 (ddd, *J* = 8.5, 7.2, 1.5 Hz, 1H), 7.75 (d, *J* = 7.7 Hz, 1H), 7.57-7.51 (m, 1H), 7.19-7.10 (m, 2H), 3.90 (s, 3H).. ^{13}C NMR (101 MHz, DMSO-d_6): δ 162.8, 162.3, 152.3, 149.4, 135.0, 129.9, 127.8, 126.6, 126.3, 125.3, 121.2, 114.5, 55.9. The spectroscopic data matched that previously report.[7]

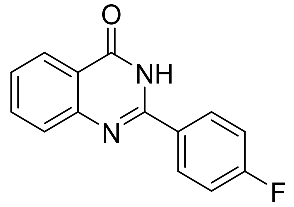

2-(4-fluorophenyl)quinazolin-4(3H)-one (5e), yellow solid; ^{1}H NMR (400 MHz, DMSO-d_6): δ 12.65 (brs, 1H), 8.40-8.28 (m, 2H), 8.22 (dd, *J* = 7.9, 1.2 Hz, 1H), 7.99-7.84 (m, 1H), 7.80 (d, *J* = 7.8 Hz, 1H), 7.69-7.53 (m, 1H), 7.53-7.38 (m, 2H). ^{13}C NMR (101 MHz, DMSO-d_6): δ 165.8, 163.3, 162.7, 150.49 (d, *J* = 275.8 Hz), 135.1, 130.85 (d, *J* = 9.0 Hz), 129.72, 129.69, 127.9, 127.1, 126.3, 121.3, 116.11 (d, *J* = 21.9 Hz, 4H). The spectroscopic data matched that previously report.[7]

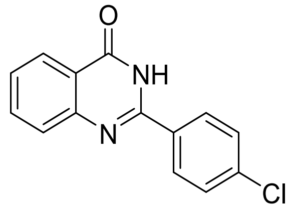

(4-chlorophenyl)(phenyl)methanone (5f), yellow solid; ^{1}H NMR (400 MHz, DMSO-d_6): δ 12.68 (brs, 1H), 8.31-8.24 (m, 2H), 8.22 (dd, J = 7.9, 1.2 Hz, 1H), 7.95-7.87 (m, 1H), 7.81 (d, J = 7.8 Hz, 1H), 7.72 -7.65 (m, 2H), 7.65-7.53 (m, 1H). ^{13}C NMR (101 MHz, DMSO-d_6): δ 162.7, 151.8, 149.0, 136.8, 135.2, 132.0, 130.1, 129.2, 127.9, 127.3, 126.4, 121.5. The spectroscopic data matched that previously report.[7]

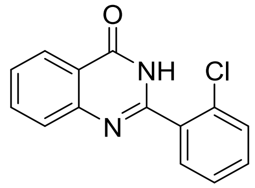

2-(4-methoxyphenyl)quinazolin-4(3H)-one (5g), yellow solid; ^{1}H NMR (400 MHz, DMSO-d_6): δ 12.69 (brs, 1H), 8.23 (dd, J = 7.9, 1.3 Hz, 1H), 7.91 (ddd, J = 8.5, 7.2, 1.6 Hz, 1H), 7.81-7.70 (m, 2H), 7.70-7.59 (m, 3H), 7.55 (td, J = 7.4, 1.4 Hz, 1H). ^{13}C NMR (101 MHz, DMSO-d_6): δ 161.9, 152.7, 149.1, 135.1, 134.3, 132.1, 131.9, 131.4, 130.1, 127.9, 127.7, 127.6, 126.3, 121.7. The spectroscopic data matched that previously report.[8]

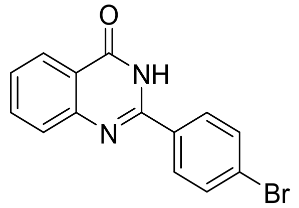

2-(4-bromophenyl)quinazolin-4(3H)-one (5h), yellow solid; ^{1}H NMR (400 MHz, DMSO-d_6): δ 12.68 (brs, 1H), 8.25-8.16 (m, 3H), 7.91 (ddd, J = 8.5, 7.2, 1.5 Hz, 1H), 7.8607.79 (m, 3H), 7.64-7.57 (m, 1H). ^{13}C NMR (101 MHz, DMSO-d_6): δ 162.6, 151.9, 149.0, 135.2, 132.4, 132.1, 130.3, 128.0, 127.3, 126.4, 125.7, 121.5. The spectroscopic data matched that previously report.[7]

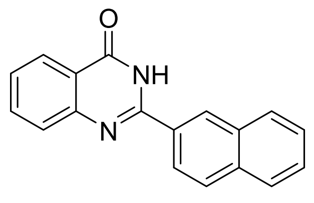

2-(naphthalen-2-yl)quinazolin-4(3H)-one (5i), White solid; ^{1}H NMR (400 MHz, DMSO-d_6): δ 12.67 (brs, 1H), 8.83 (s, 1H), 8.31 (dd, *J* = 8.6, 1.8 Hz, 1H), 8.19 (dd, *J* = 7.9, 1.2 Hz, 1H), 8.08 (t, *J* = 8.3 Hz, 2H), 8.02 (d, *J* = 7.4 Hz, 1H), 7.89-7.83 (m, 1H), 7.81 (d, *J* = 7.9 Hz, 1H), 7.65 (ddd, *J* = 8.7, 6.6, 1.1 Hz, 2H), 7.59-7.50 (m, 1H). ^{13}C NMR (101 MHz, DMSO-d_6): δ 162.7, 152.7, 149.2, 135.2, 134.6, 132.8, 130.4, 129.4, 128.7, 128.5, 128.4, 128.1, 128.05, 127.4, 127.2, 126.4, 125.0, 121.5. The spectroscopic data matched that previously report.[7]

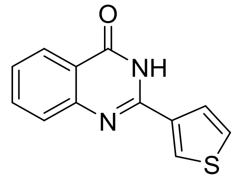

2-(thiophen-3-yl)quinazolin-4(3H)-one (5j), yellow solid; ^{1}H NMR (400 MHz, DMSO-d_6): δ 12.47 (brs, 1H), 8.61 (dd, *J* = 2.7, 1.1 Hz, 1H), 8.14 (dd, *J* = 7.9, 1.1 Hz, 1H), 7.88 (dd, *J* = 5.1, 1.0 Hz, 1H), 7.85-7.78 (m, 1H), 7.71 (dd, *J* = 8.6, 5.9 Hz, 2H), 7.56-7.41 (m, 1H). ^{13}C NMR (101 MHz, DMSO-d_6): δ 162.55 (brs, 1H), 149.3, 148.7, 135.8, 135.1, 129.1, 127.8, 127.8, 127.5, 126.8, 126.3, 121.4. The spectroscopic data matched that previously report.[8]

2-(thiophen-2-yl)quinazolin-4(3H)-one (5k), yellow solid; ^{1}H NMR (400 MHz, DMSO): δ 12.75 (brs, 1H), 8.36 (t, *J* = 8.7 Hz, 2H), 8.18 (dd, *J* = 7.9, 1.1 Hz, 1H), 7.99-7.81 (m, 3H), 7.78 (d, *J* = 7.8 Hz, 1H), 7.62-7.48 (m, 1H). ^{13}C NMR (101 MHz, DMSO): δ 162.1, 149.8 (d, $J_{C\text{-}F}$ = 353.5 Hz), 136.6, 134.6, 131.0 (q, $J_{C\text{-}F}$ =32.5 Hz), 128.5, 127.3, 127.0, 125.5, 125.4 (q, $J_{C\text{-}F}$ = 3.8 Hz), 121.6. The spectroscopic data matched that previously report.[7]

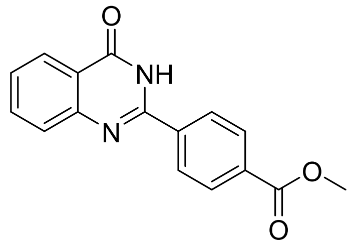

methyl 4-(4-oxo-3,4-dihydroquinazolin-2-yl)benzoate (5l), yellow solid; ^{1}H NMR (400 MHz, DMSO-d_6): δ 12.70 (brs, 1H), 8.31 (d, *J* = 8.5 Hz, 2H), 8.18 (dd, *J* = 7.9, 1.2 Hz, 1H), 8.11 (dd, *J* = 8.4, 5.0 Hz, 2H), 7.90-7.82 (m, 1H), 7.78 (d, *J* = 7.9 Hz, 1H), 7.59-7.51 (m, 1H), 3.91 (s, 3H). ^{13}C NMR (101 MHz, DMSO-d_6): δ 166.2, 162.6, 151.9, 148.9, 137.4, 135.2, 132.2, 129.7, 128.7, 128.1, 127.5, 126.4, 121.6, 52.9. The spectroscopic data matched that previously report.[9]

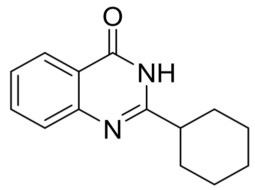

2-cyclohexylquinazolin-4(3H)-one (5m), yellow solid; ^{1}H NMR (400 MHz, DMSO-d_6): δ 12.09 (brs, 1H), 8.07 (d, *J* = 7.1 Hz, 1H), 7.82-7.69 (m, 1H), 7.59 (d, *J* = 8.1 Hz, 1H), 7.44 (t, *J* = 7.4 Hz, 1H), 2.57 (ddd, *J* = 11.7, 7.5, 3.3 Hz, 1H), 1.90 (d, *J* = 12.1 Hz, 2H), 1.78 (d, *J* = 12.6 Hz, 2H), 1.70-1.51 (m, 3H), 1.38-1.14 (m, 3H). ^{13}C NMR (101 MHz, DMSO-d_6): δ 162.4, 161.2, 149.4, 134.7, 127.4, 126.4, 126.1, 121.4, 43.29 (s, 2H), 30.7, 25.9, 25.8. The spectroscopic data matched that previously report.[7]

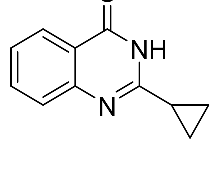

2-cyclopropylquinazolin-4(3H)-one (5n), yellow solid; ^{1}H NMR (400 MHz, DMSO-d_6): δ 12.47 (brs, 1H), 8.09 (dd, *J* = 7.9, 1.2 Hz, 1H), 7.76 (ddd, *J* = 8.5, 7.2, 1.6 Hz, 1H), 7.52 (d, *J* = 8.0 Hz, 1H), 7.48-7.31 (m, 1H), 2.27-1.84 (m, 1H), 1.14 (qd, *J* = 4.7, 2.4 Hz, 2H), 1.07 (ddd, *J* = 10.4, 6.6, 3.7 Hz, 2H). ^{13}C NMR (101 MHz, DMSO-d_6): δ 162.1, 159.5, 149.6, 134.7, 126.9, 126.2, 125.7, 121.1, 13.9, 9.9. The spectroscopic data matched that previously report.[10]

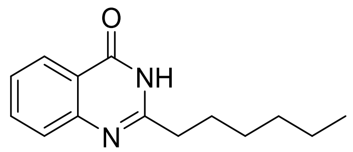

2-hexylquinazolin-4(3H)-one (5o), yellow solid; ^{1}H NMR (400 MHz, DMSO-d_6): δ 12.16 (brs, 1H), 8.08 (dd, J = 7.9, 1.3 Hz, 1H), 7.76 (ddd, J = 8.5, 7.2, 1.6 Hz, 1H), 7.59 (d, J = 8.0 Hz, 1H), 7.54-.25 (m, 1H), 2.64-.54 (m, 2H), 1.78-.60 (m, 2H), 1.41-1.13 (m, 6H), 0.85 (dd, J = 8.7, 5.2 Hz, 3H). ^{13}C NMR (101 MHz, DMSO-d_6): δ 162.3, 157.9, 149.4, 134.7, 127.3, 126.3, 126.1, 121.2, 34.9, 31.4, 28.7, 27.2, 22.4, 14.4. The spectroscopic data matched that previously report.[7]

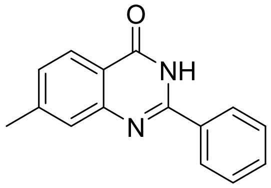

7-methyl-2-phenylquinazolin-4(3H)-one (5p), White solid; ^{1}H NMR (400 MHz, DMSO-d_6): δ 12.45 (brs, 1H), 8.18 (dd, J = 8.1, 1.4 Hz, 2H), 8.04 (d, J = 8.1 Hz, 1H), 7.65-7.47 (m, 4H), 7.34 (dd, J = 8.1, 1.1 Hz, 1H), 2.47 (s, 3H). ^{13}C NMR (101 MHz, DMSO-d_6): δ 162. 6, 152.7, 149.3, 145.5, 133.2, 131.8, 129.1, 128.5, 128.2, 127.6, 126.2, 119.1, 21.8. The spectroscopic data matched that previously report.[7]

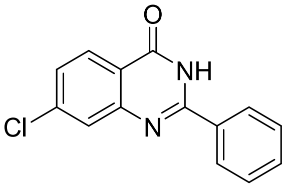

7-chloro-2-phenylquinazolin-4(3H)-one (5q), White solid; ^{1}H NMR (400 MHz, DMSO-d_6): δ 12.67 (brs, 1H), 8.24-8.08 (m, 3H), 7.80 (d, J = 2.0 Hz, 1H), 7.66-7.44 (m, 4H). ^{13}C NMR (101 MHz, DMSO-d_6): δ 162.1, 154.3, 150.4, 139.7, 132.8, 132.2, 129.1, 128.4, 127.3, 127.1, 120.3. The spectroscopic data matched that previously report.[7]

^{1}H and ^{13}C NMR spectra of products

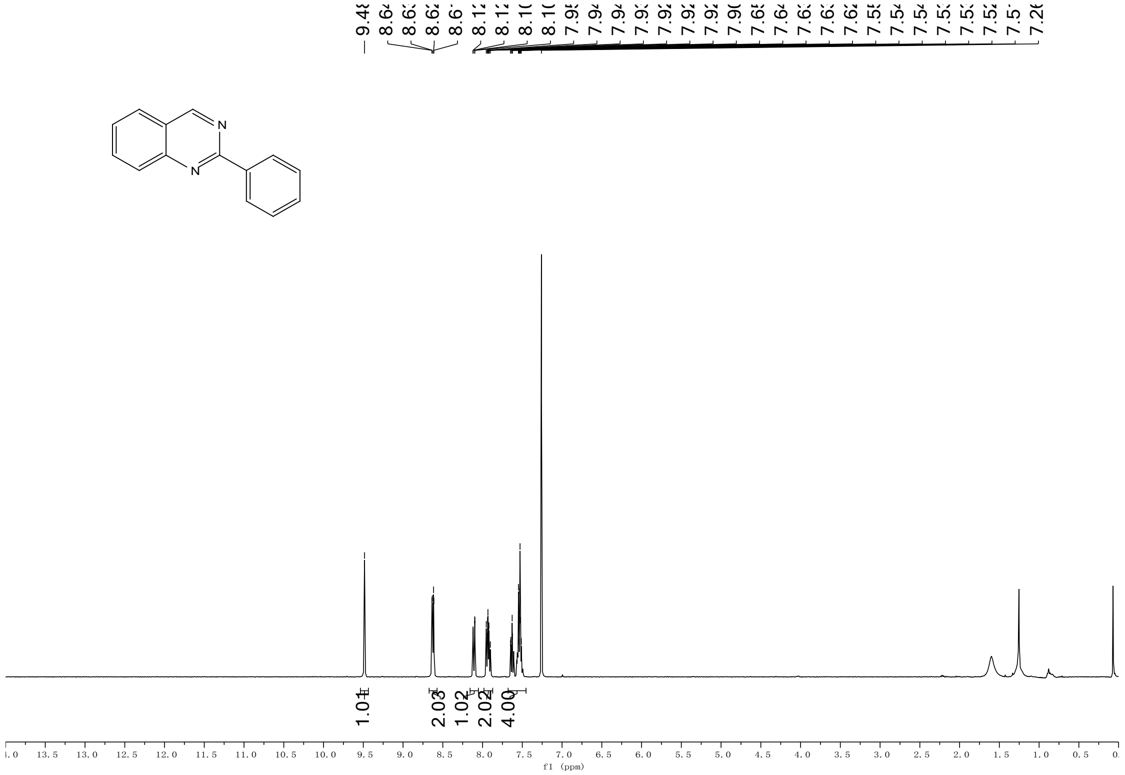

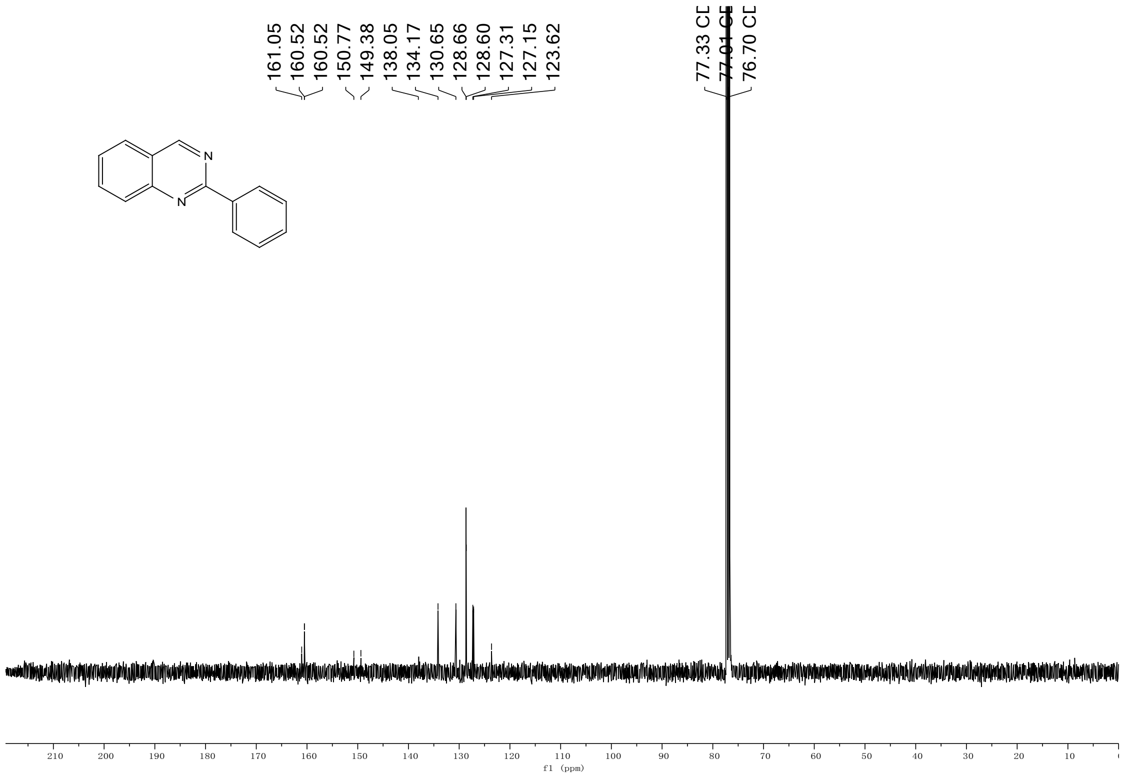

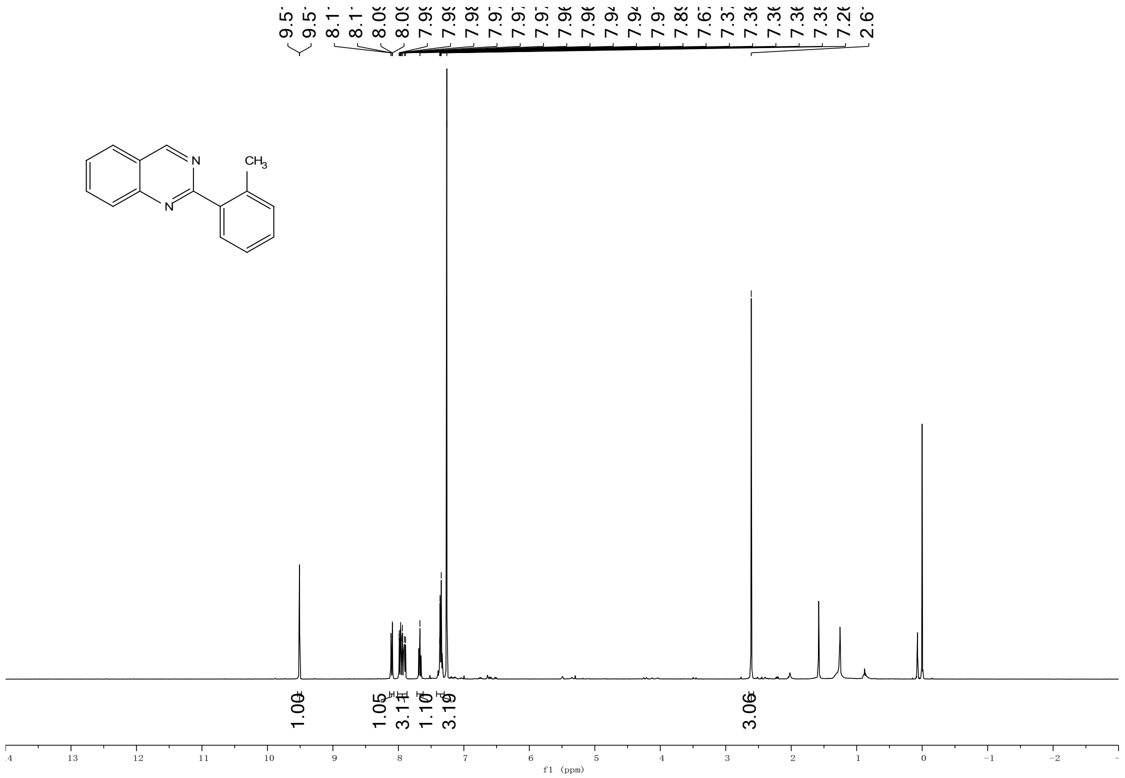

CH3
N
N
1.00
1.05
3.11
1.10
3.19
3.06
f1 (ppm)

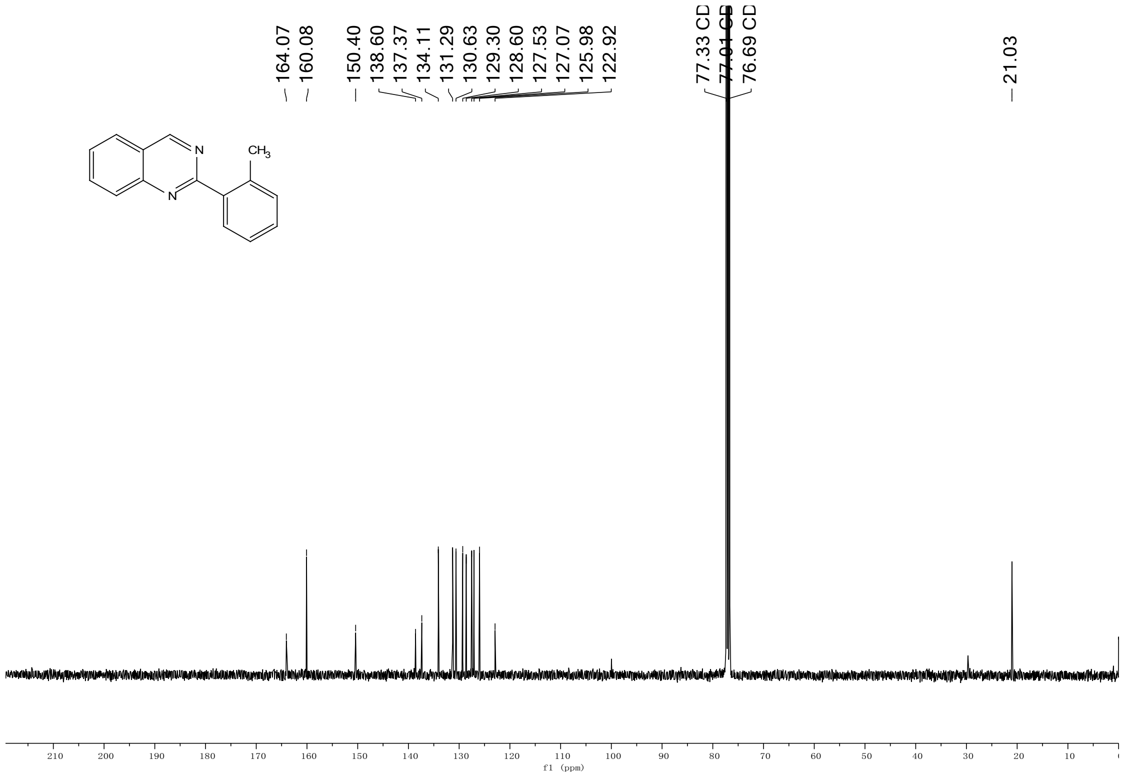

164.07
160.08
150.40
138.60
137.37
134.11
131.29
130.63
129.30
128.60
127.53
127.07
125.98
122.92
77.33 CD
76.69 CD
21.03
CH3
N
N
f1 (ppm)

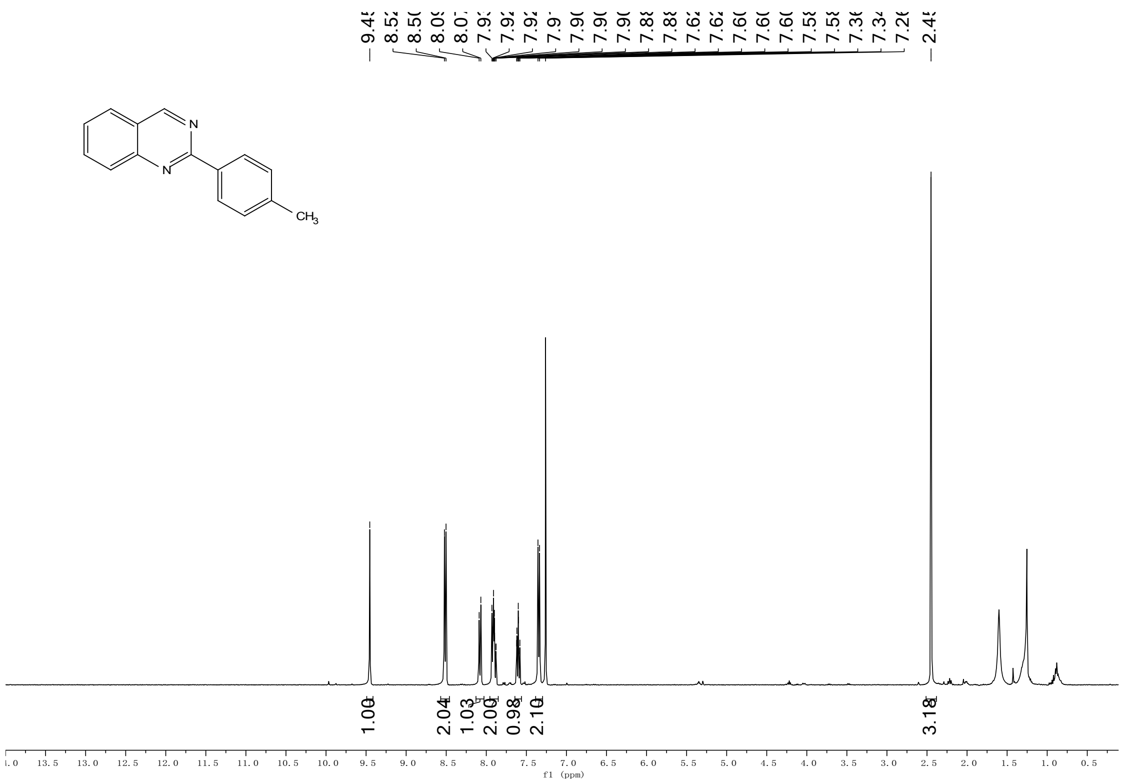
N
N
CH3
f1 (ppm)

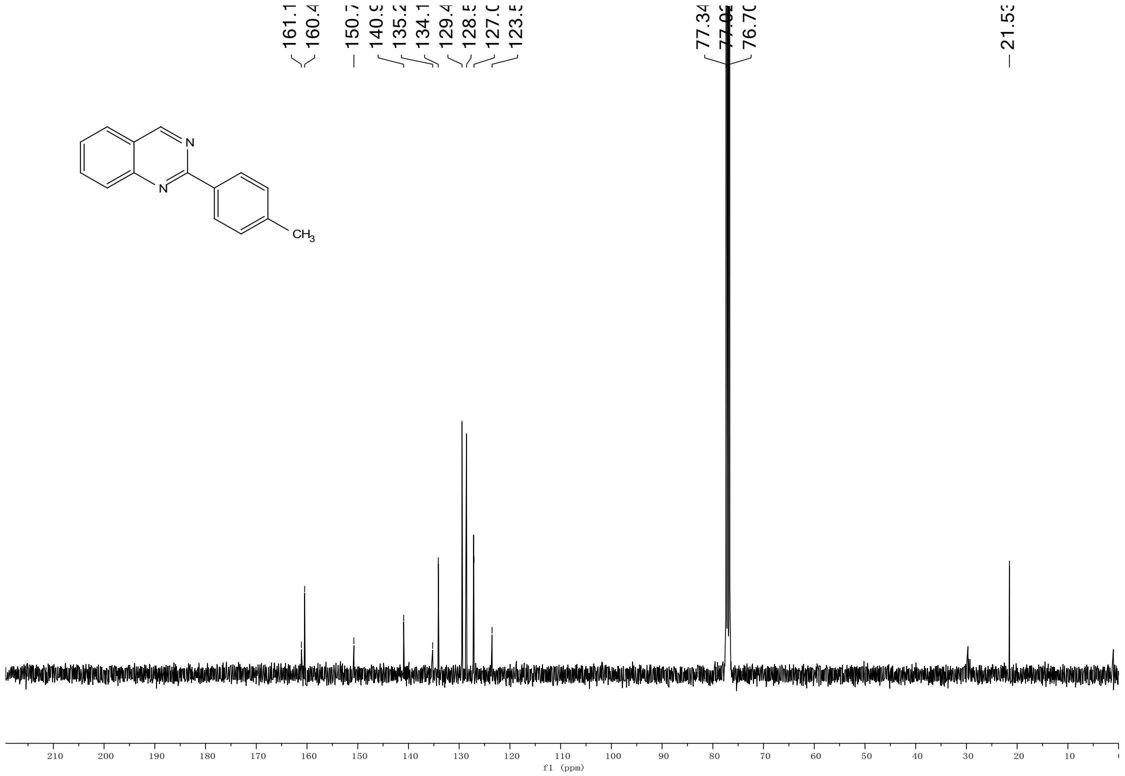
N
N
CH3
f1 (ppm)

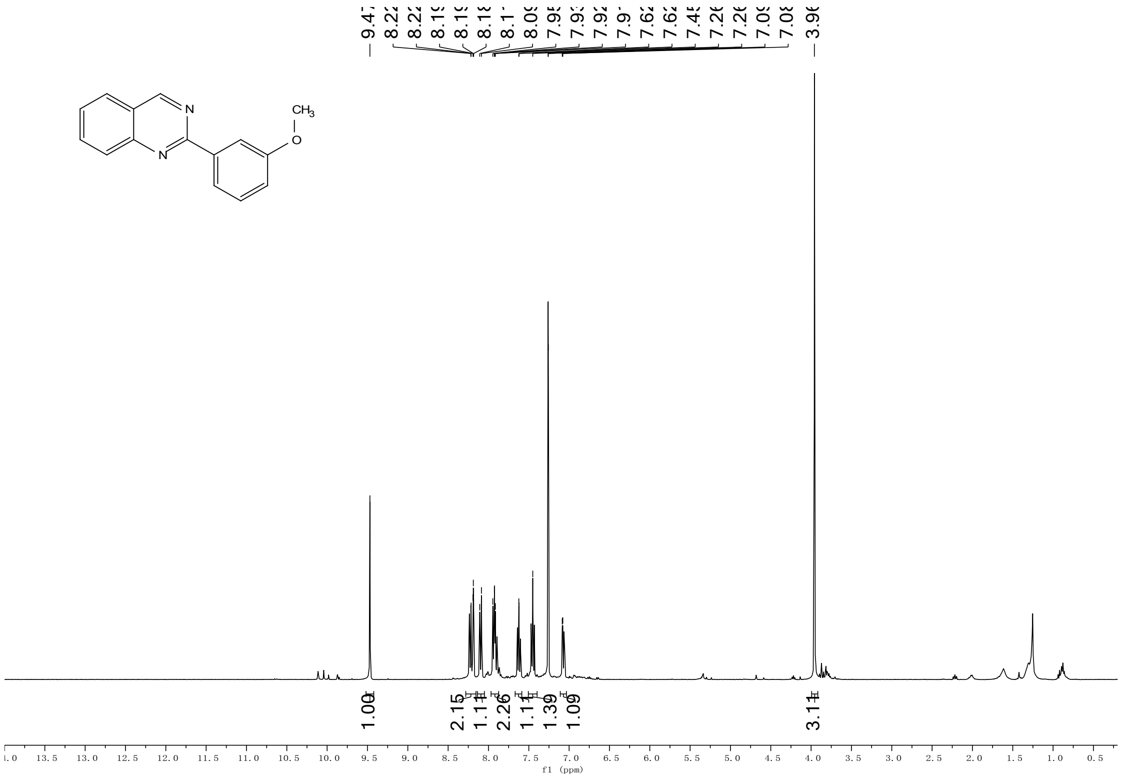
N
N
CH3
O
3.96
1.00
2.15
1.11
2.26
1.11
1.39
1.09
3.11
f1 (ppm)

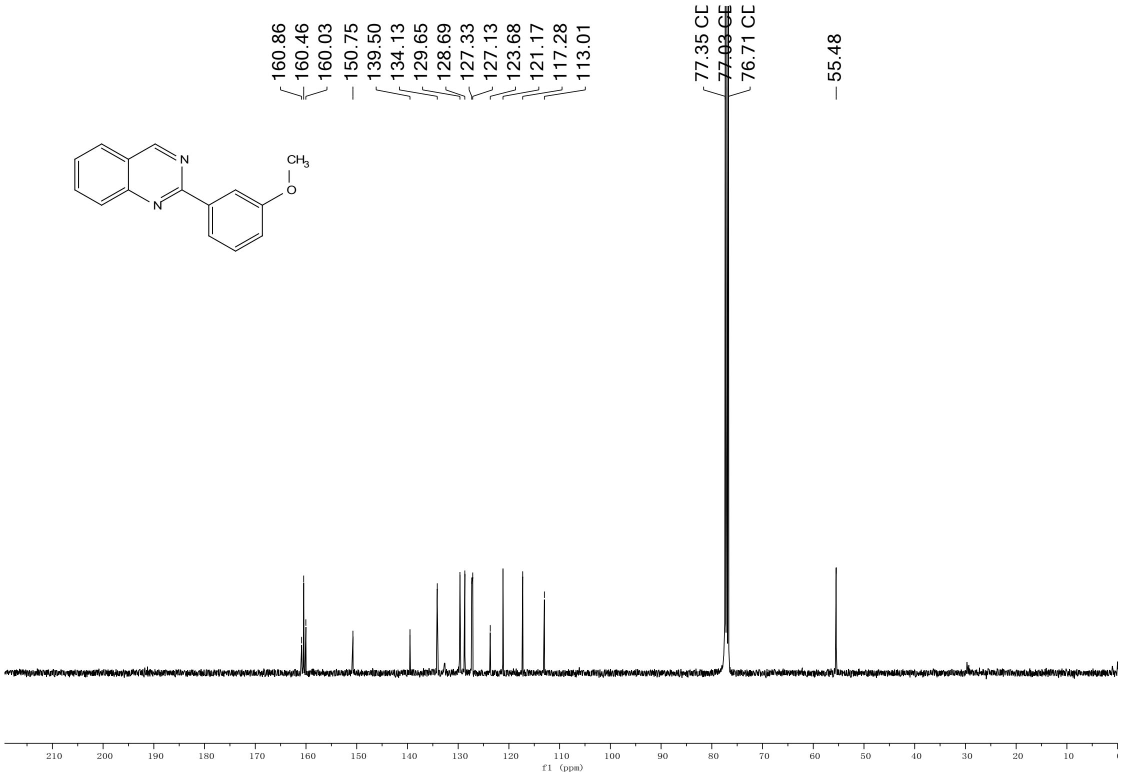
N
N
CH3
O
160.86
160.46
160.03
150.75
139.50
134.13
129.65
128.69
127.33
127.13
123.68
121.17
117.28
113.01
77.35 CD
77.03 CD
76.71 CD
55.48
f1 (ppm)

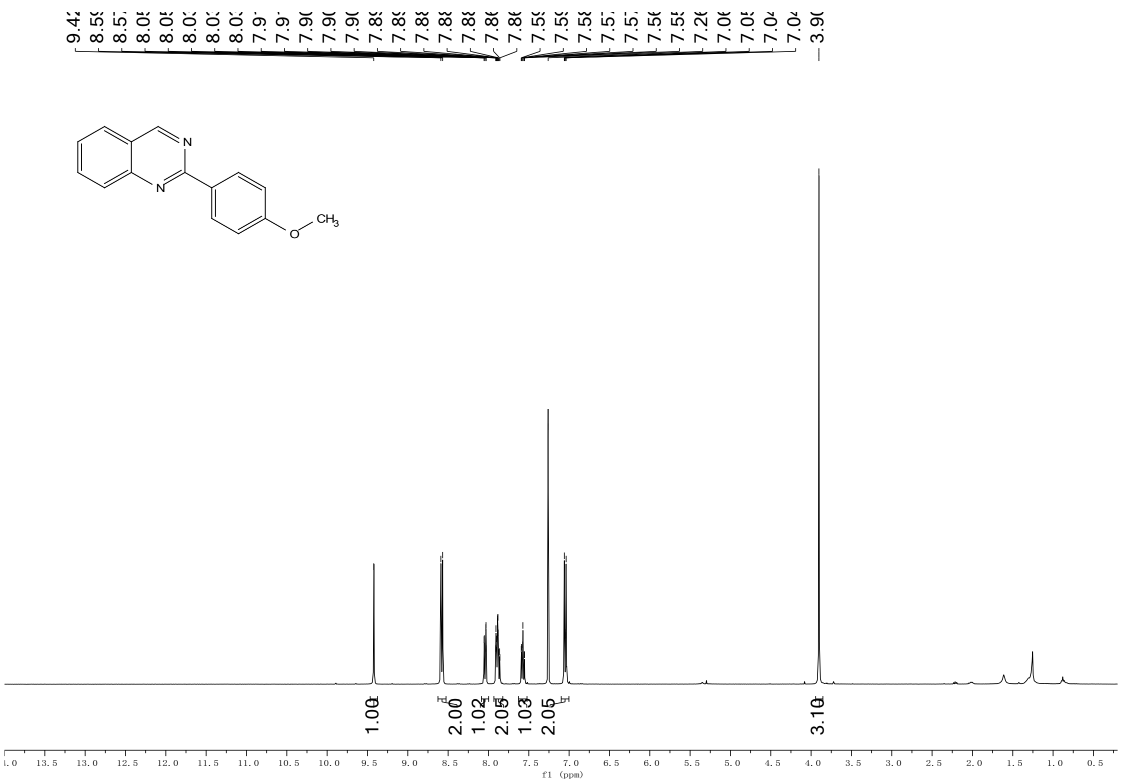
9.42
3.90
N
N
O
CH3
1.00
2.00
1.02
1.03
2.05
3.10
f1 (ppm)

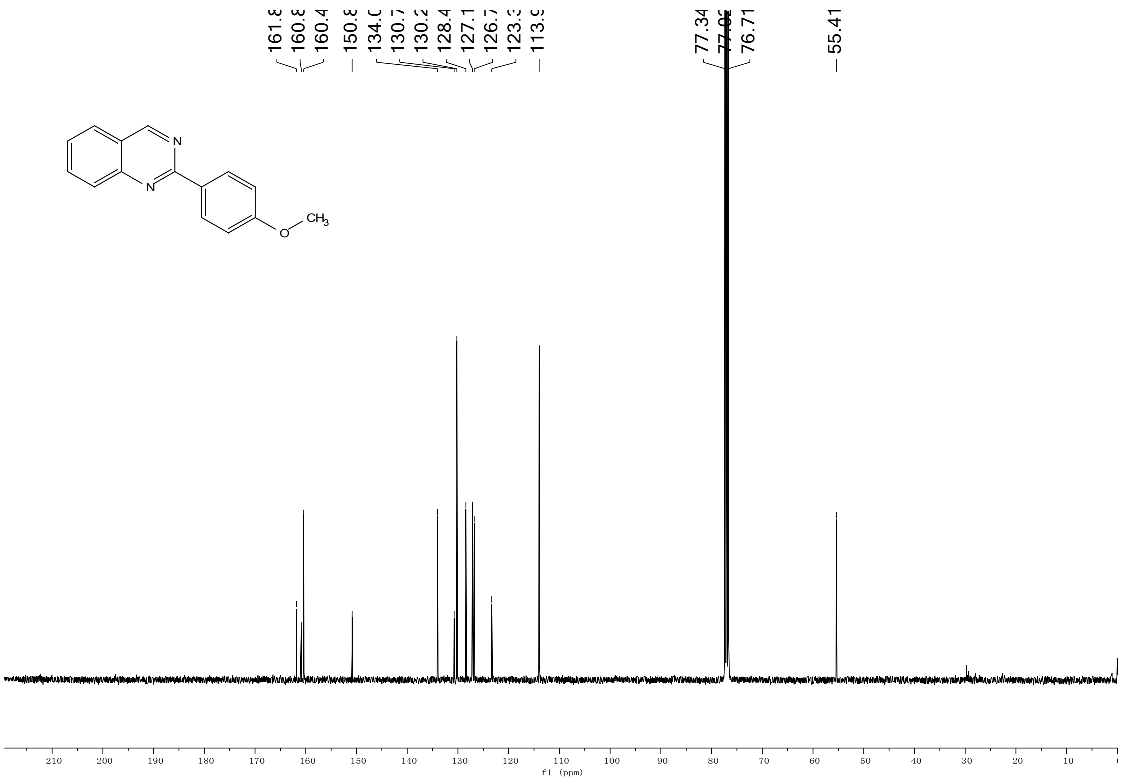
77.34
76.71
55.41
N
N
O
CH3
f1 (ppm)

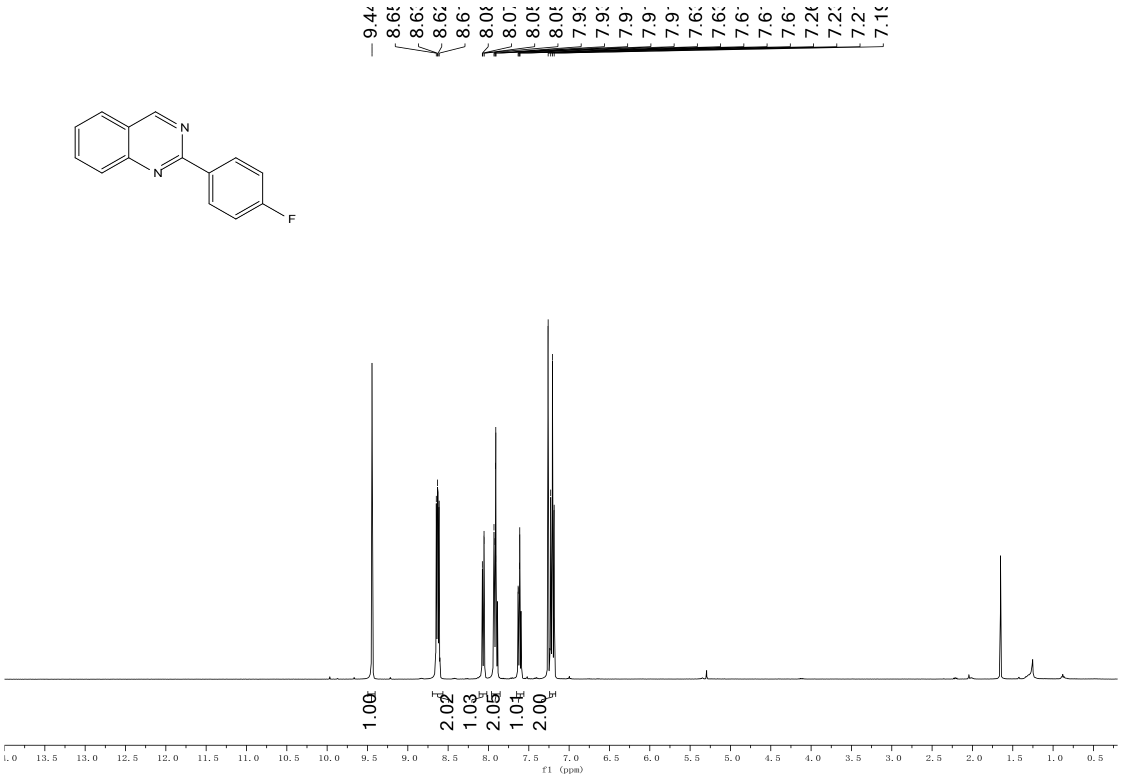
9.44
N
N
F
1.00
2.02
1.03
2.05
1.04
2.00
f1 (ppm)

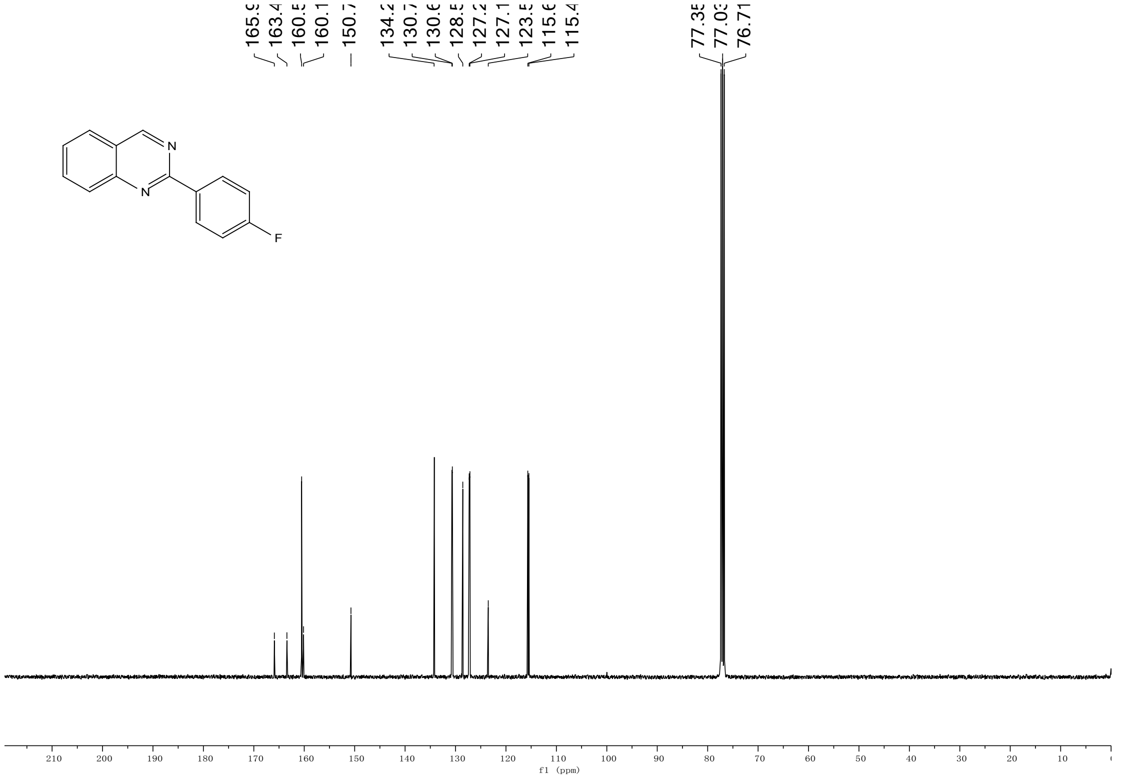
150.7
77.35
77.03
76.71
N
N
F
f1 (ppm)

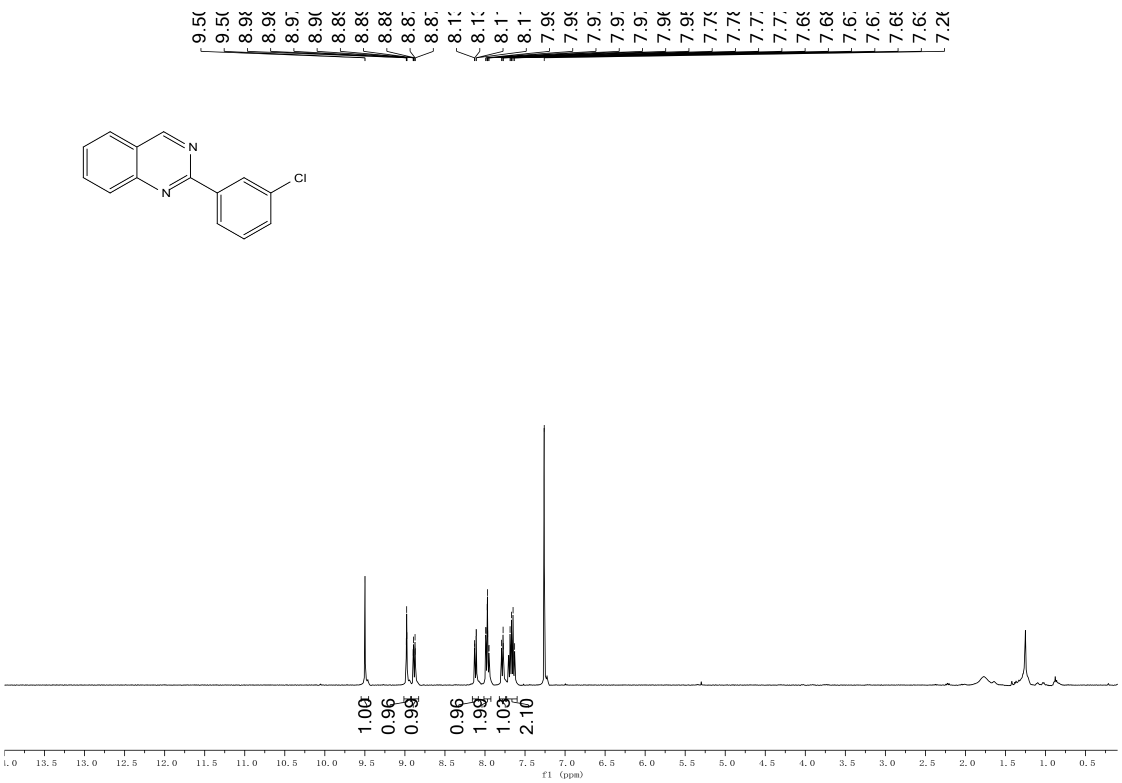
N
N
Cl
1.00
0.96
0.99
0.96
1.99
1.03
2.10
f1 (ppm)

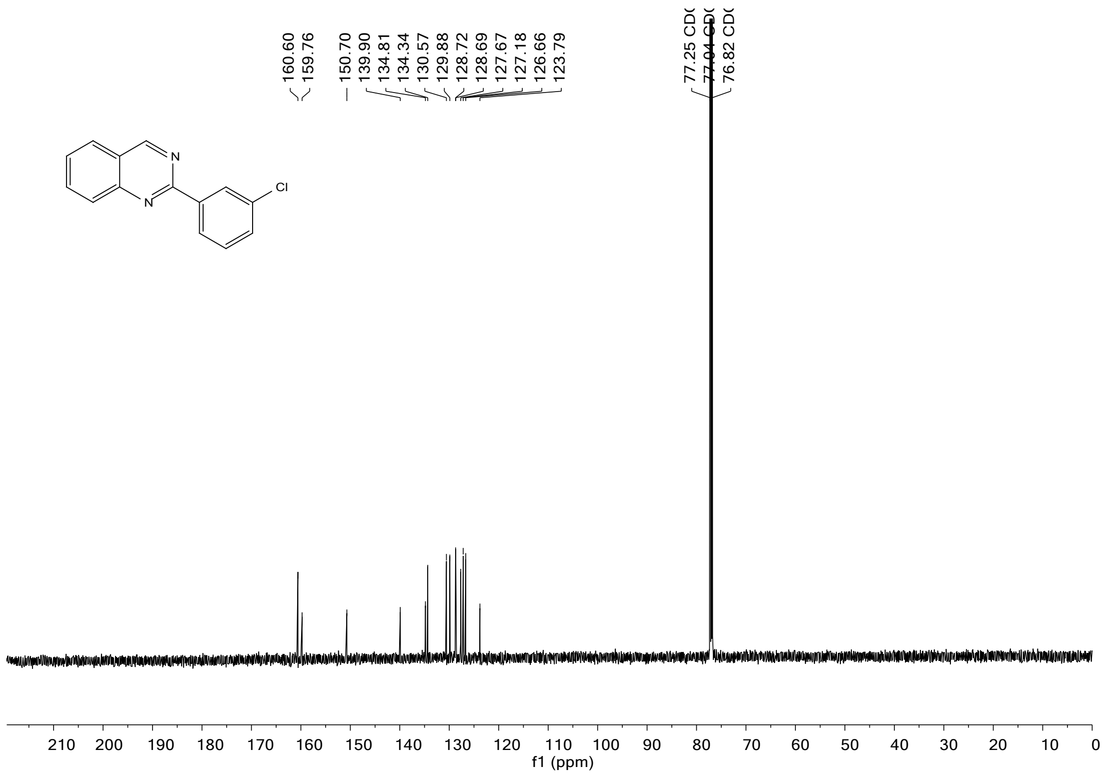
160.60
159.76
150.70
139.90
134.81
134.34
130.57
129.88
128.72
128.69
127.67
127.18
126.66
123.79
N
N
Cl
f1 (ppm)

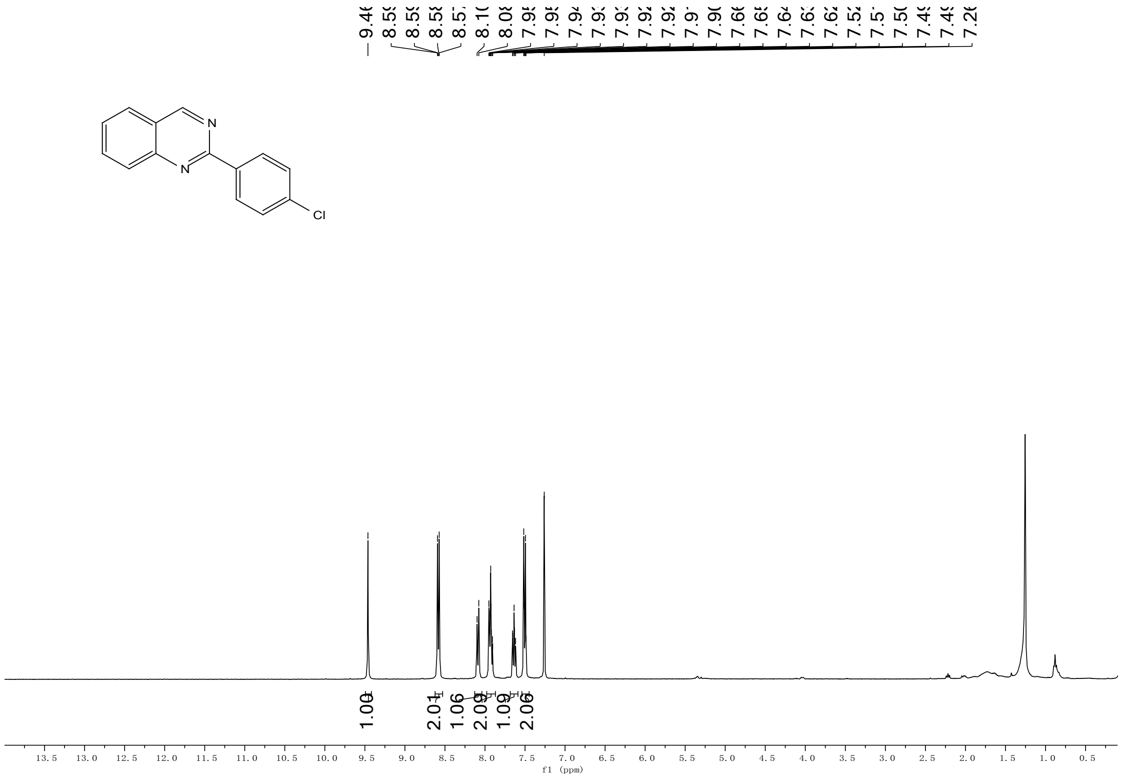
N
N
Cl
f1 (ppm)

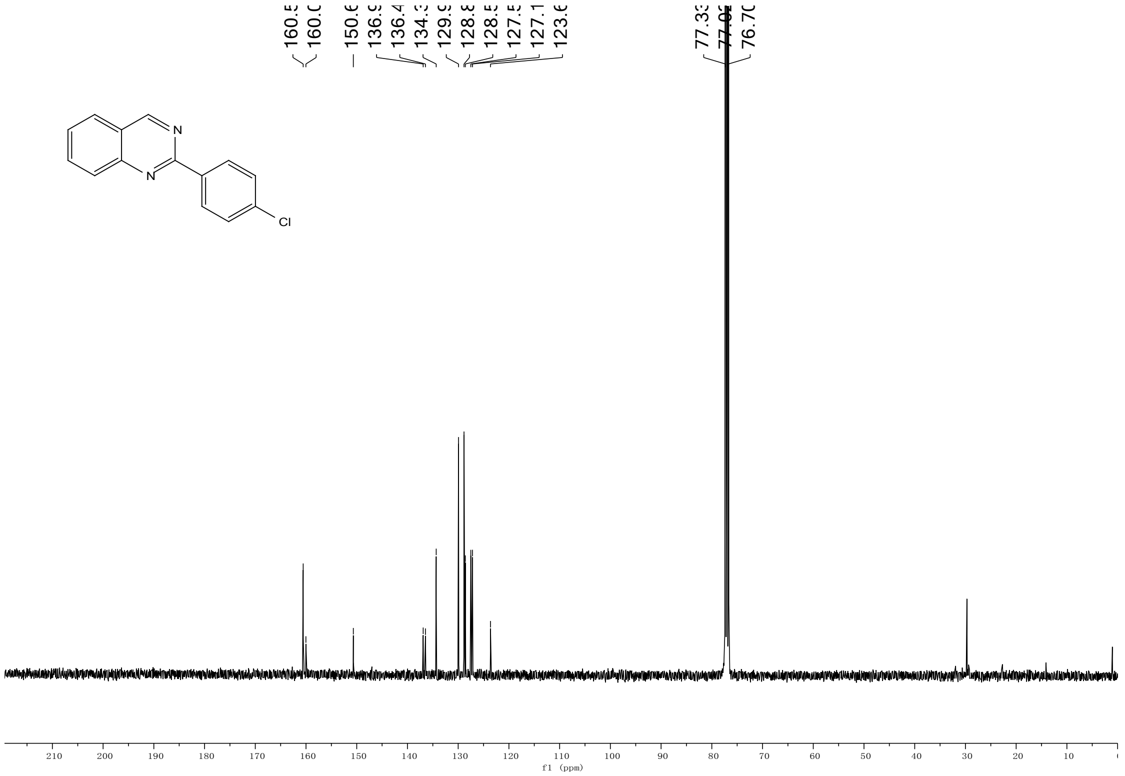
N
N
Cl
f1 (ppm)

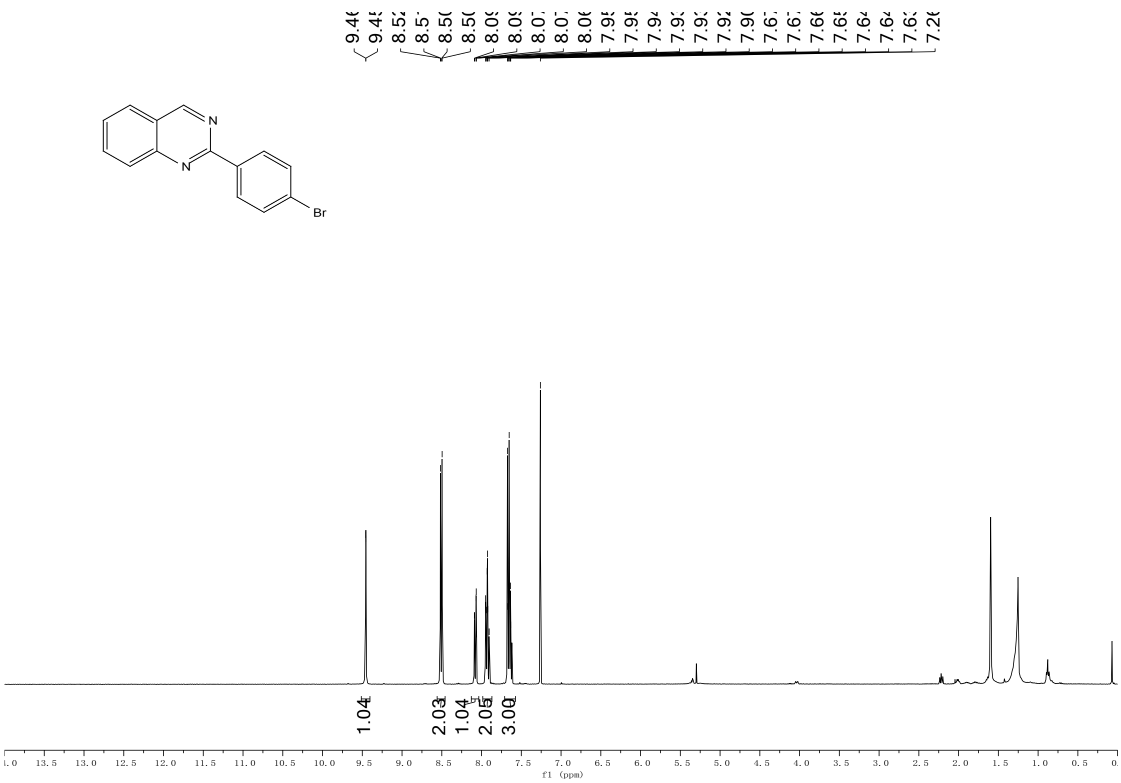
N
N
Br
f1 (ppm)

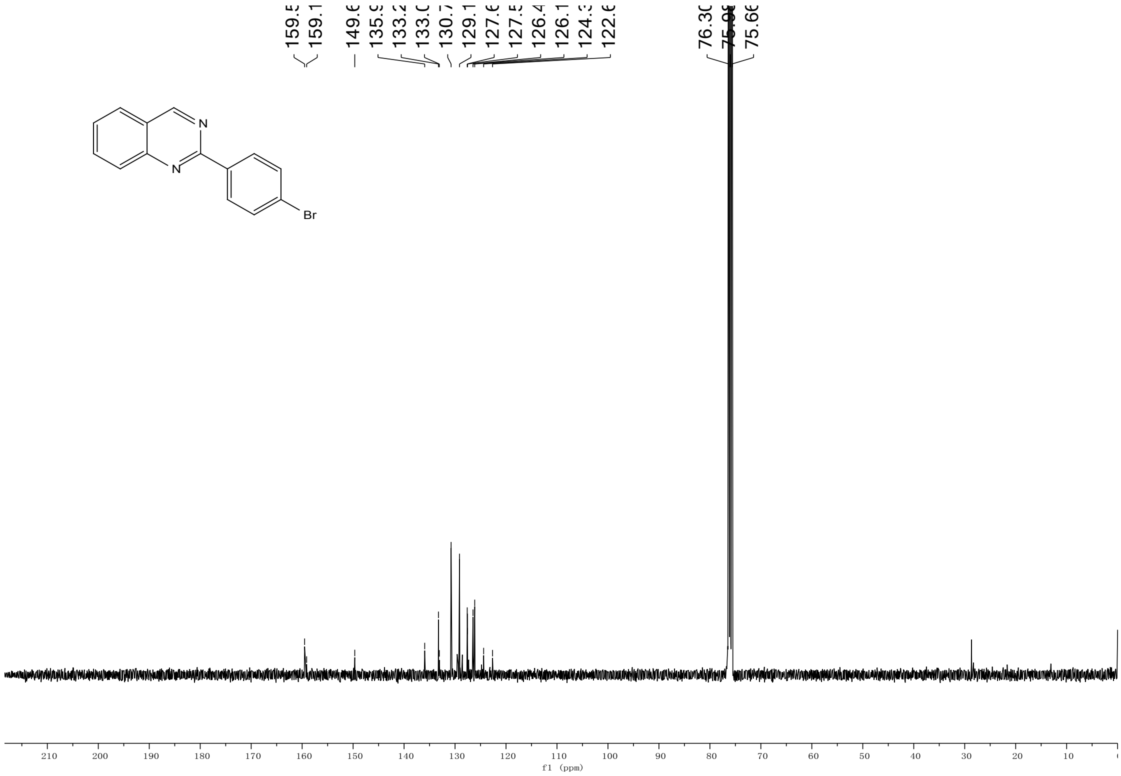
N
N
Br
f1 (ppm)

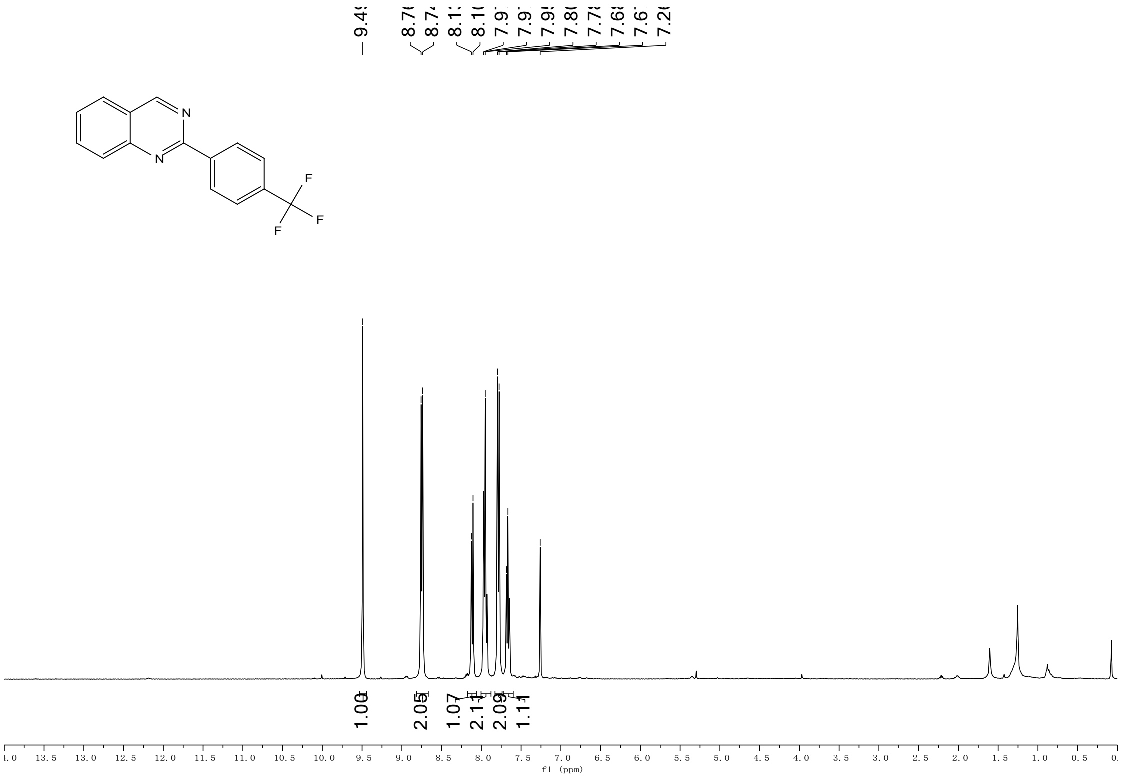
N
N
F
F
F
1.00
2.05
1.07
2.09
f1 (ppm)

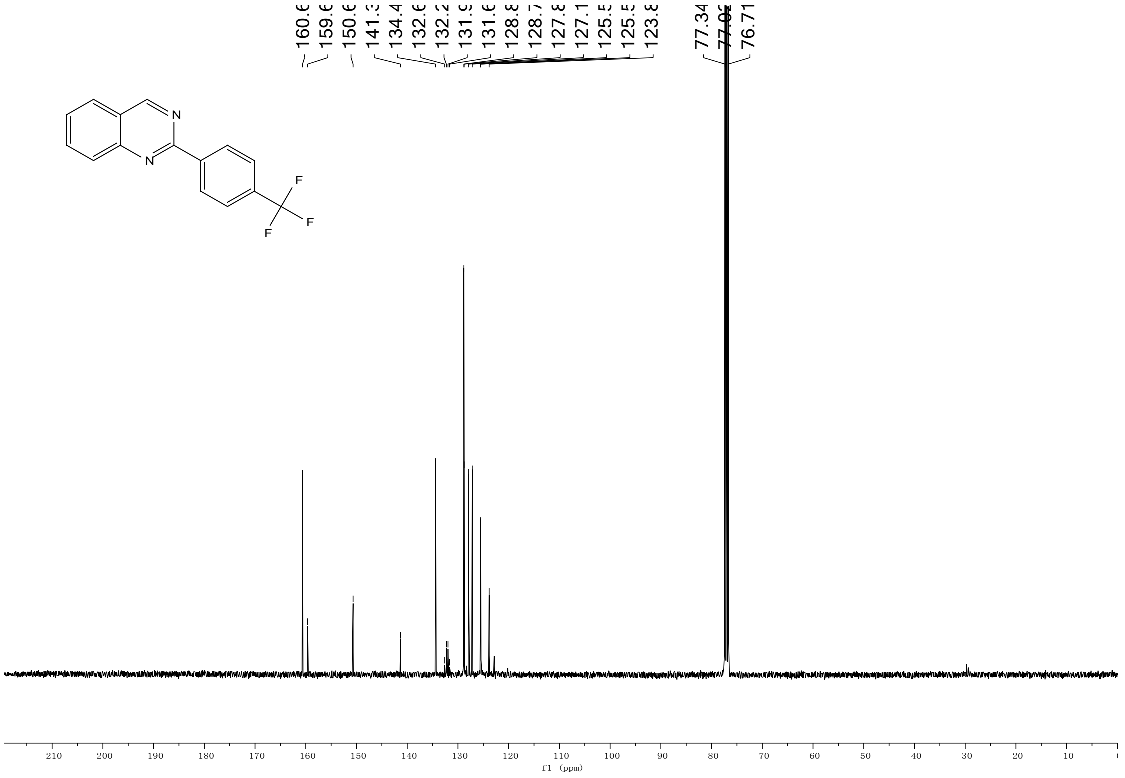
N
N
F
F
F
77.34
76.71
f1 (ppm)

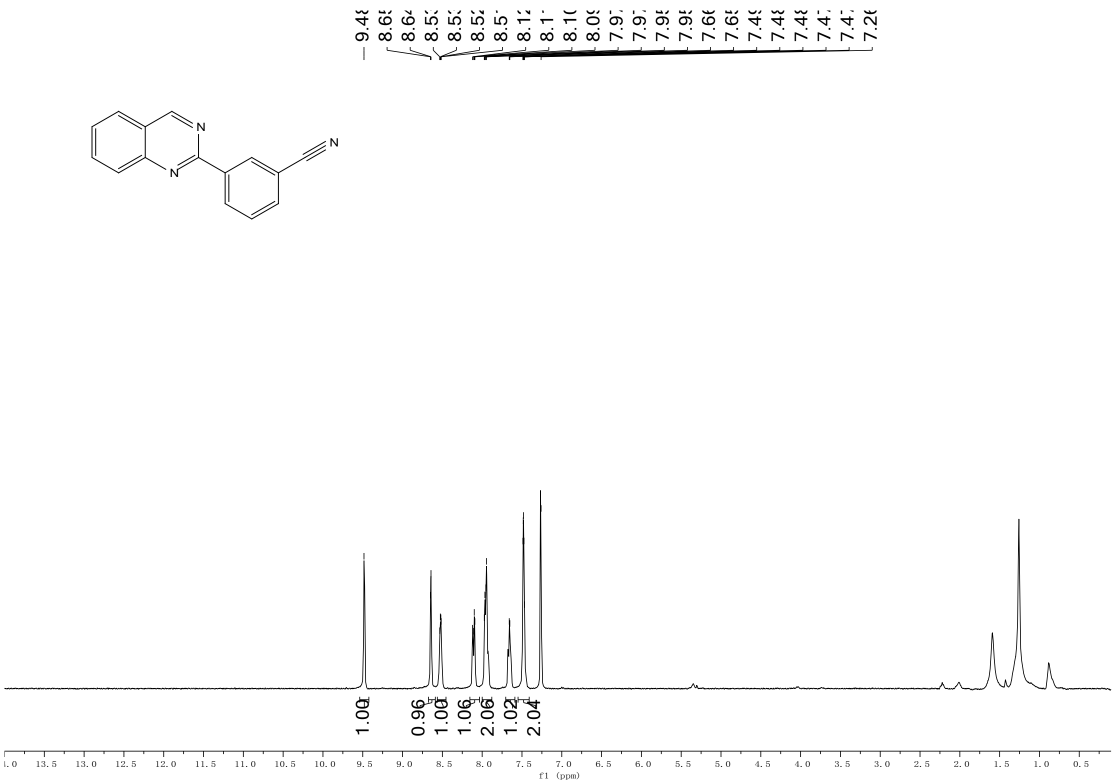

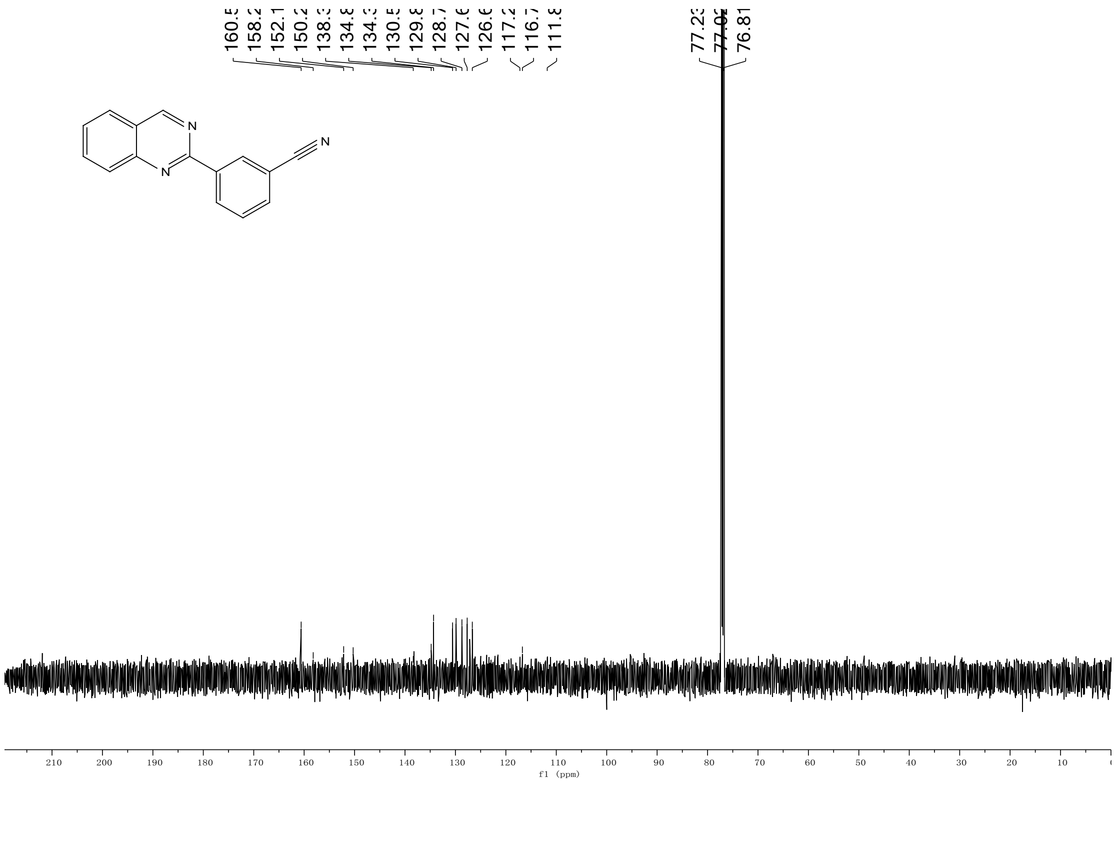

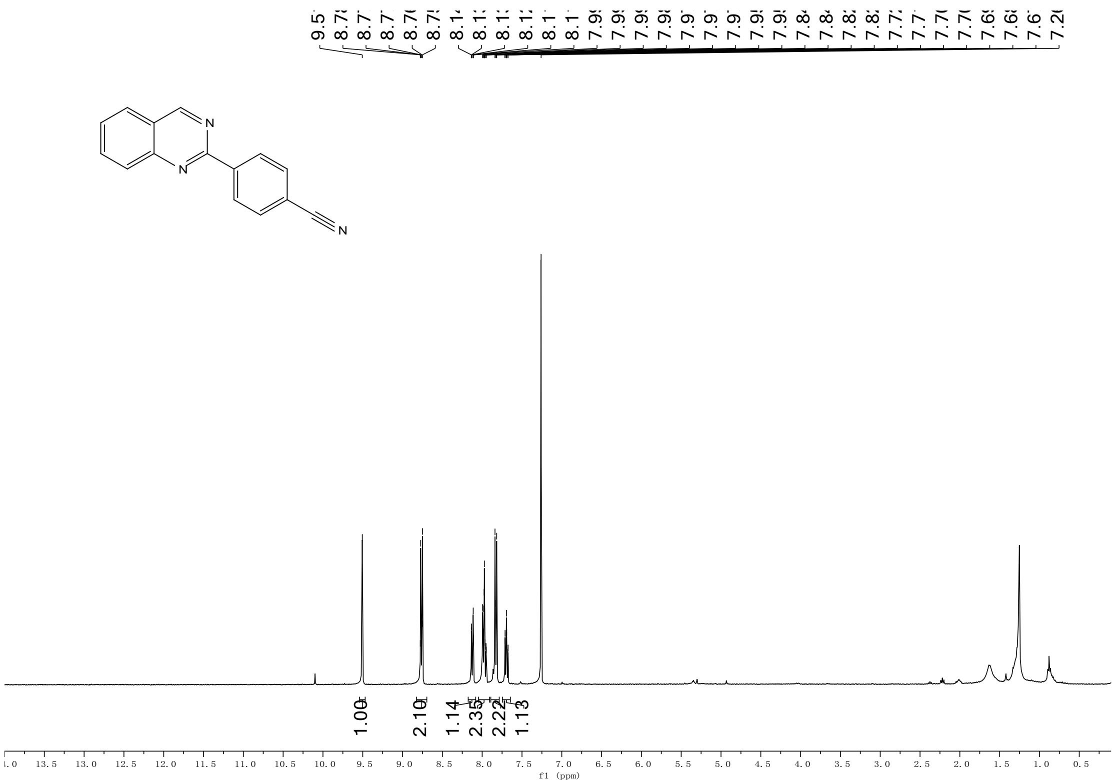
N
N
N
1.00
2.10
1.14
2.35
2.22
1.13
f1 (ppm)

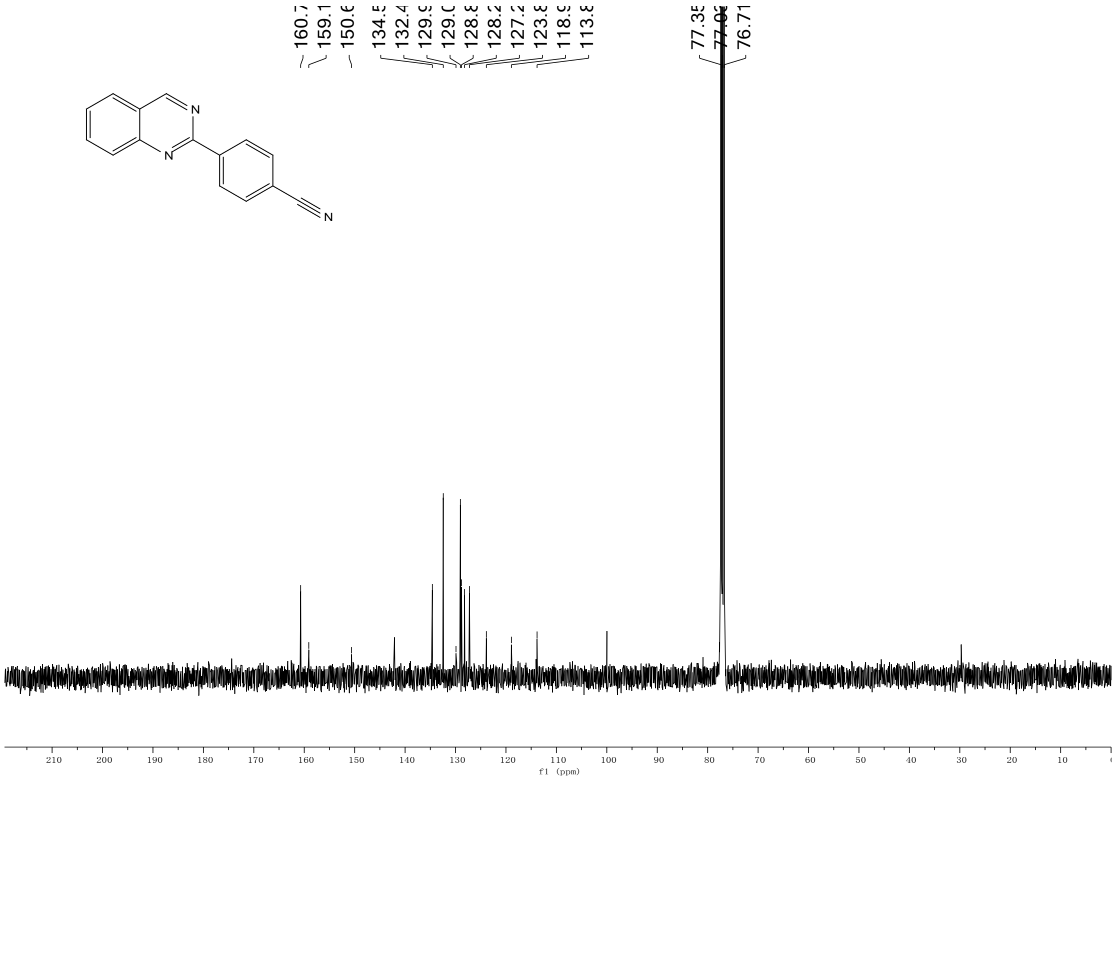
N
N
N
77.35
76.71
f1 (ppm)

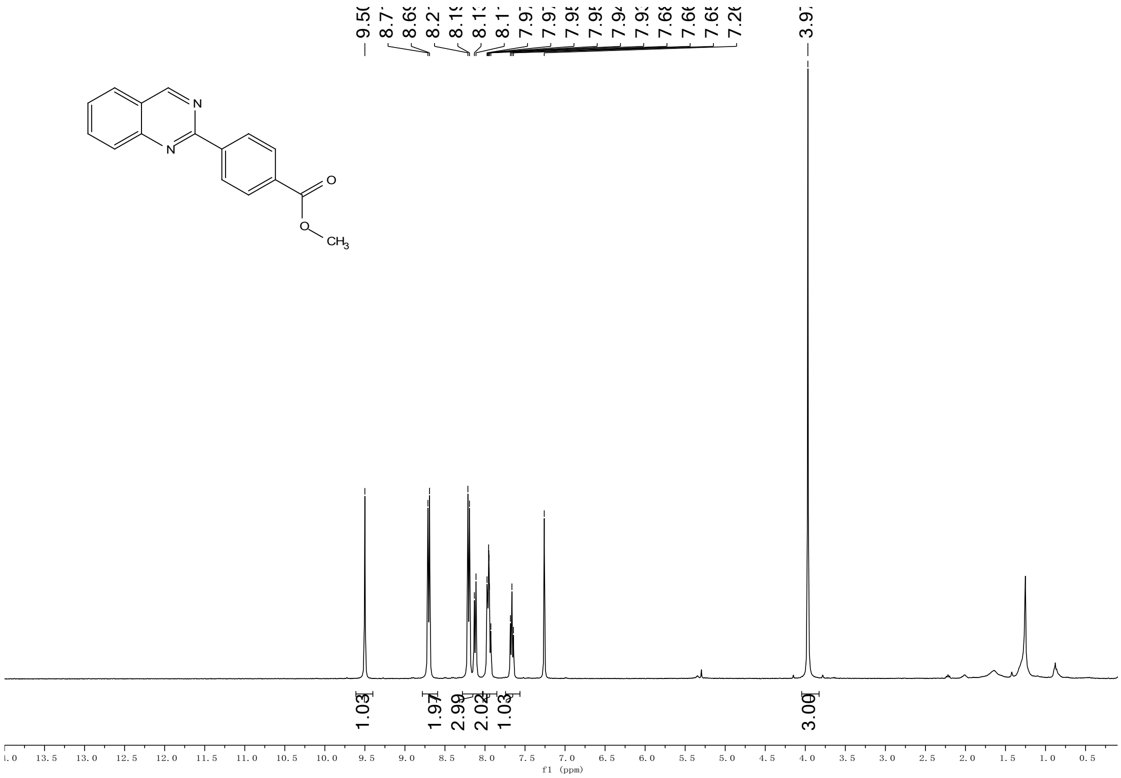
f1 (ppm)

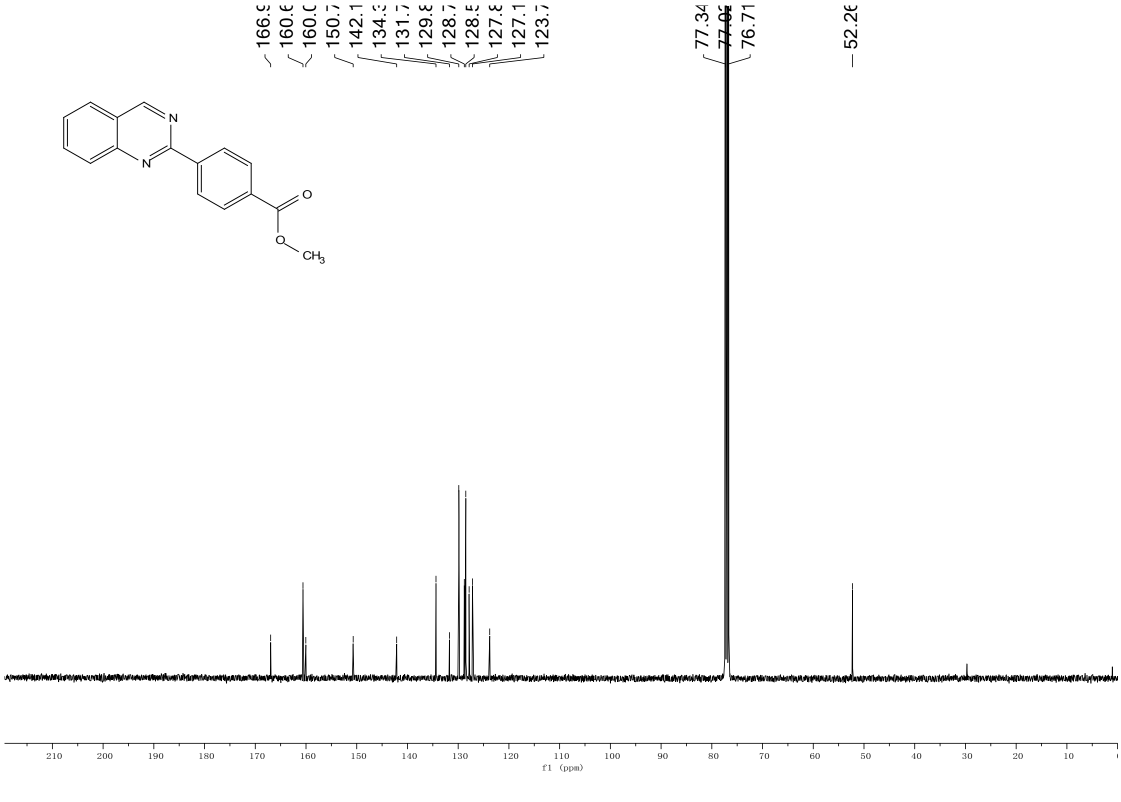
f1 (ppm)

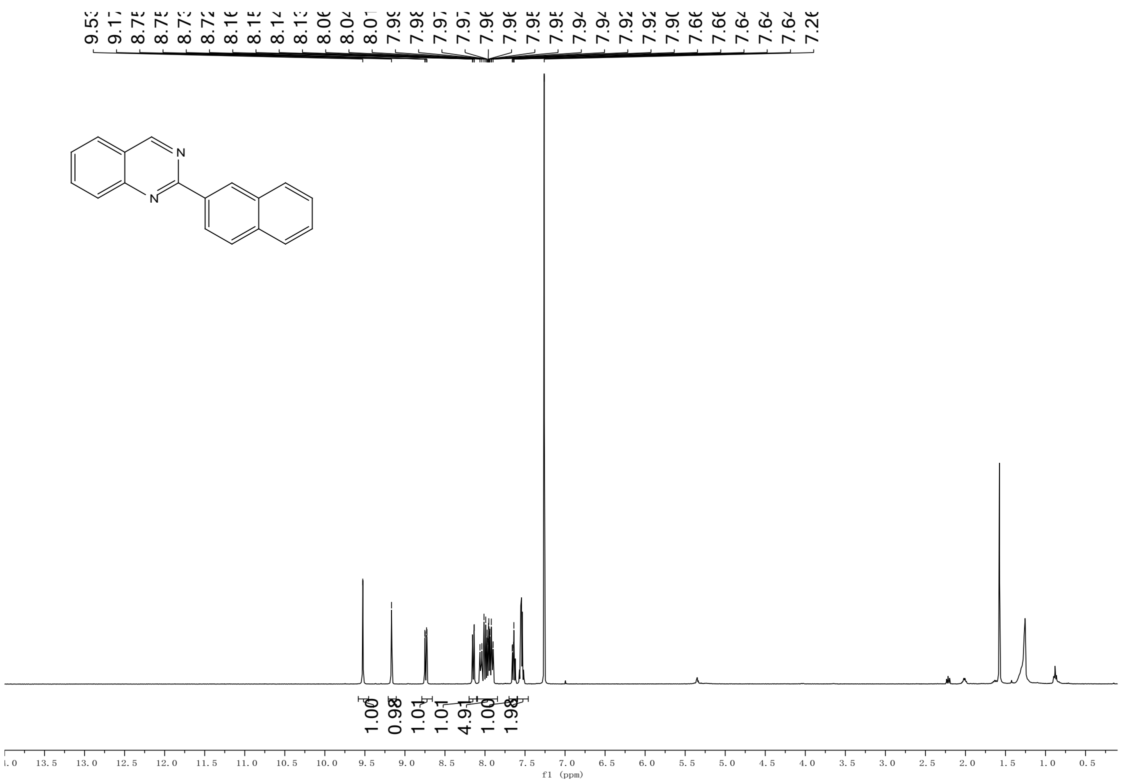

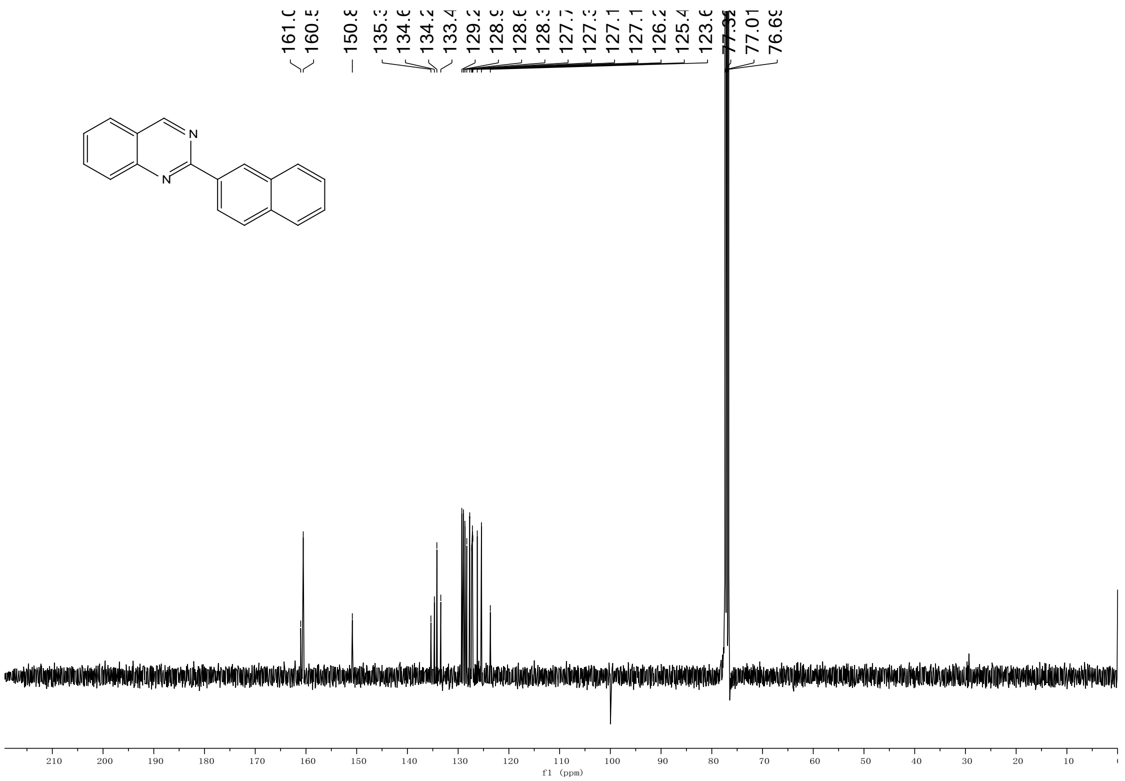

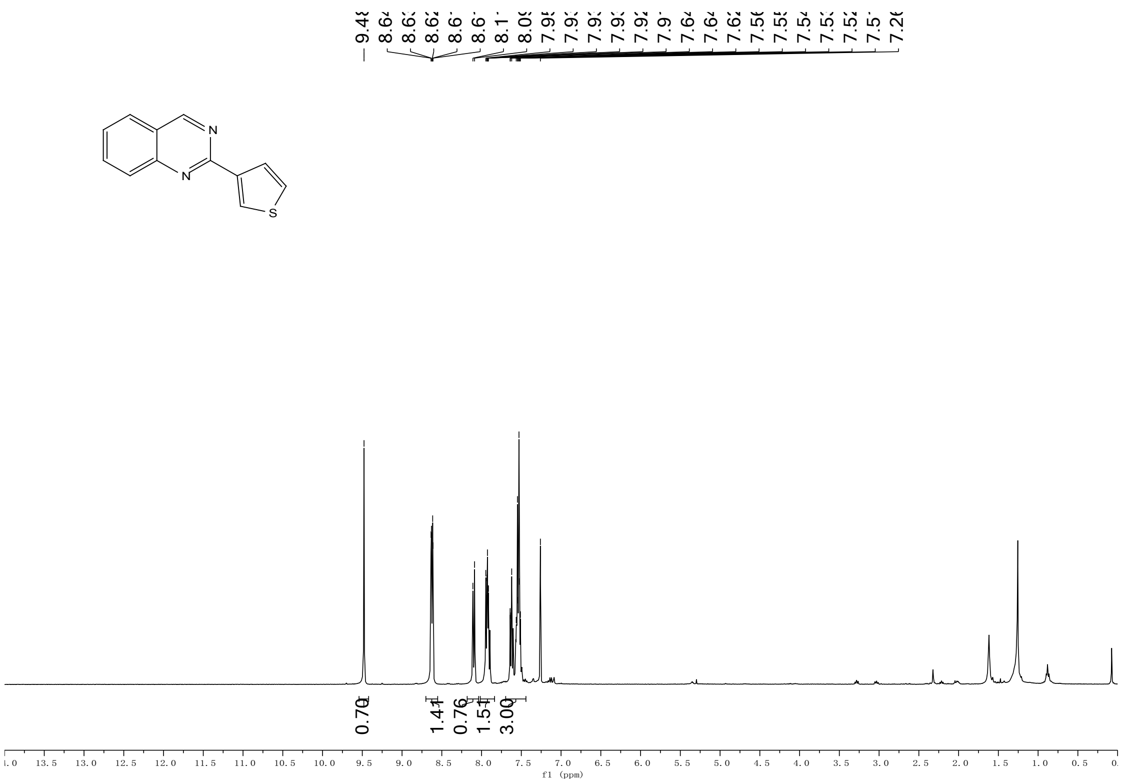

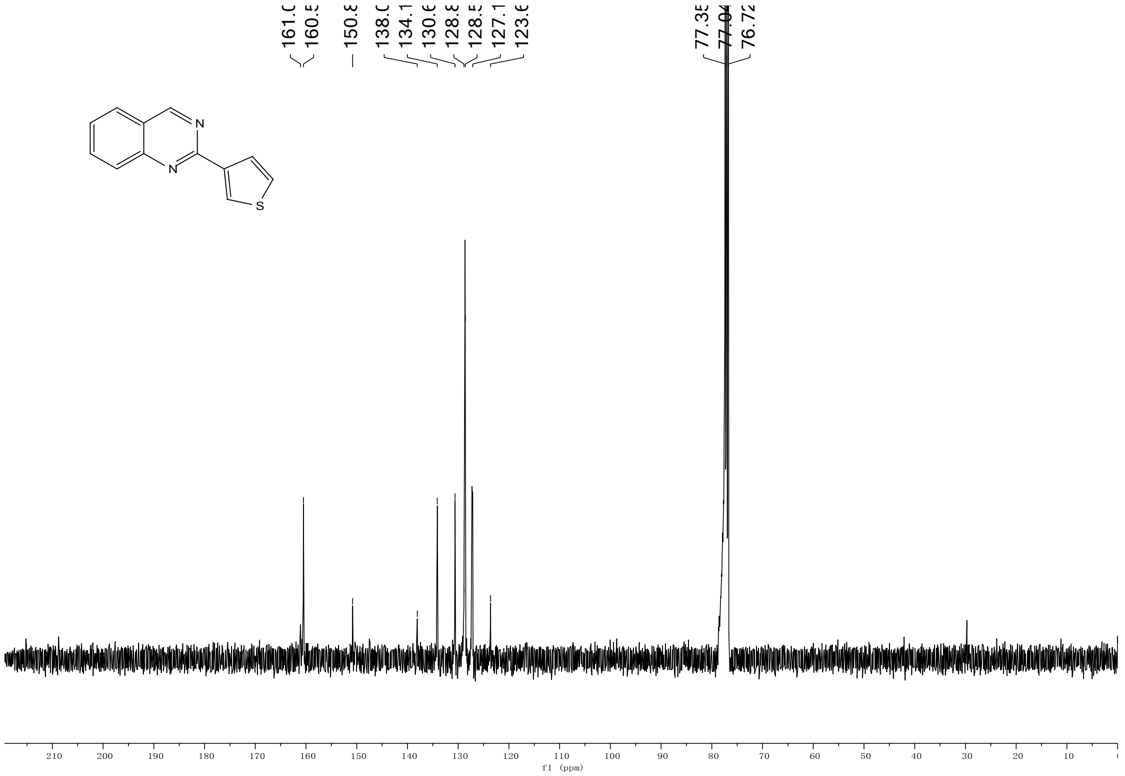

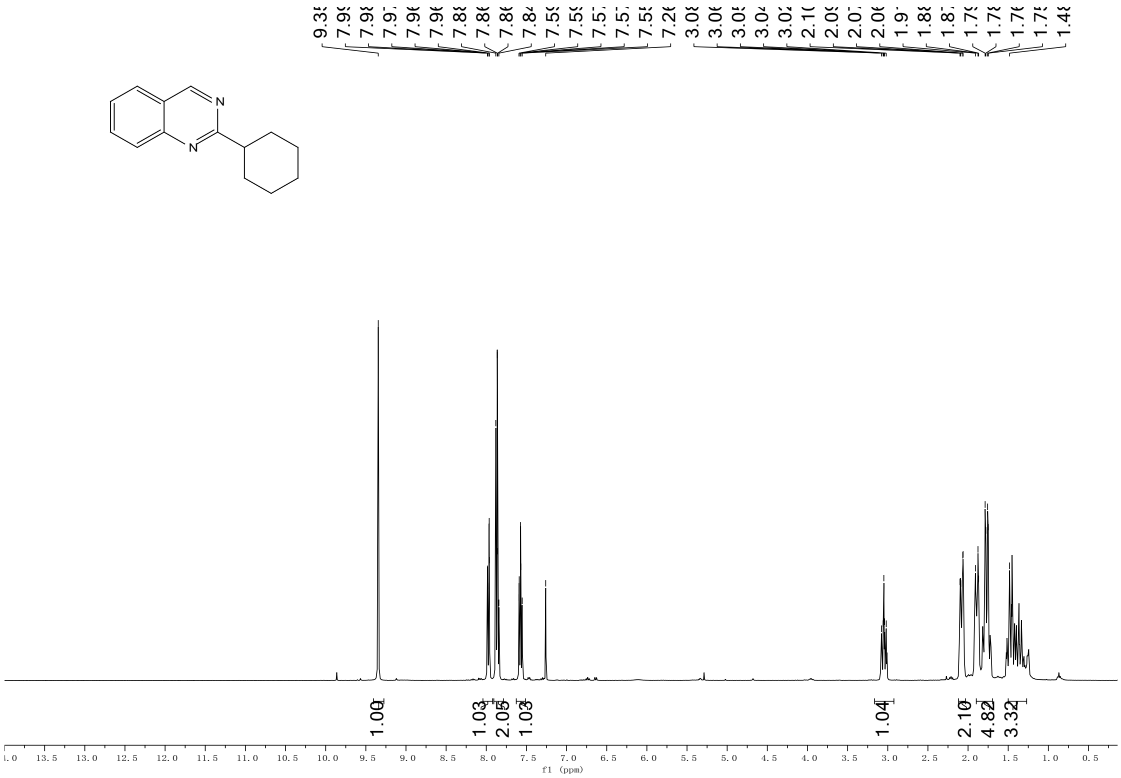

N
1.00
1.03
2.05
1.03
1.04
2.10
4.82
3.32
f1 (ppm)

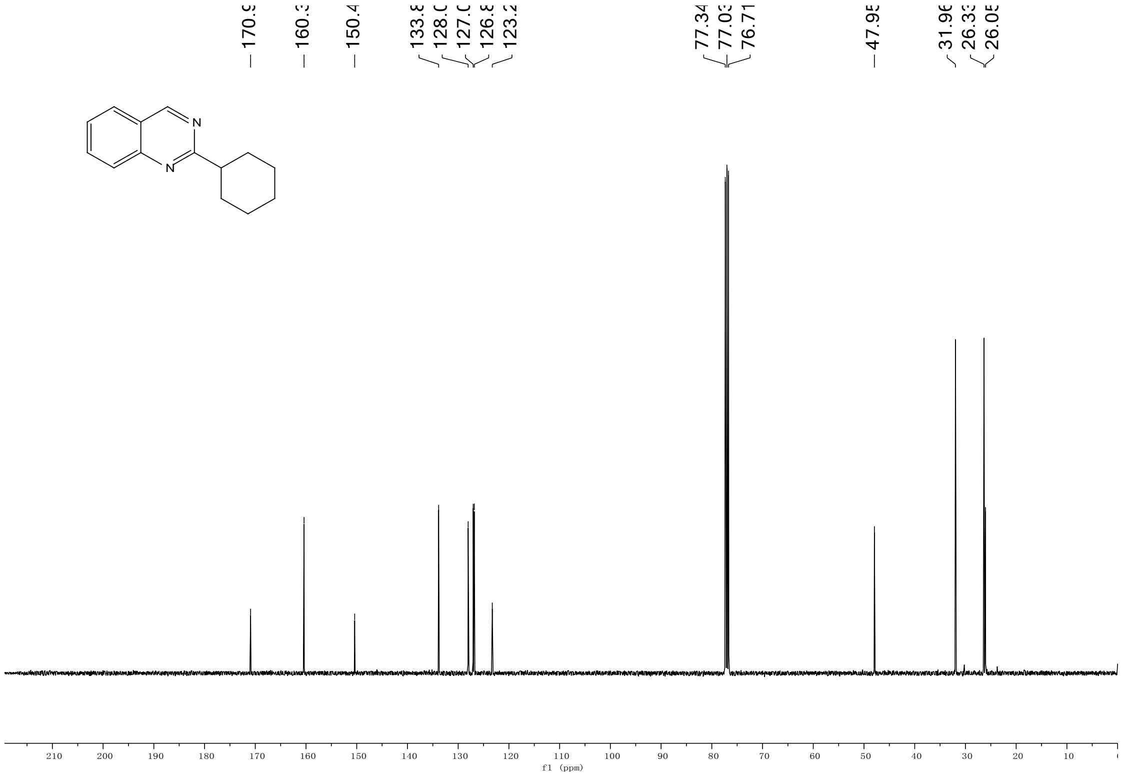

N
77.34
76.71
47.95
31.96
26.05
f1 (ppm)

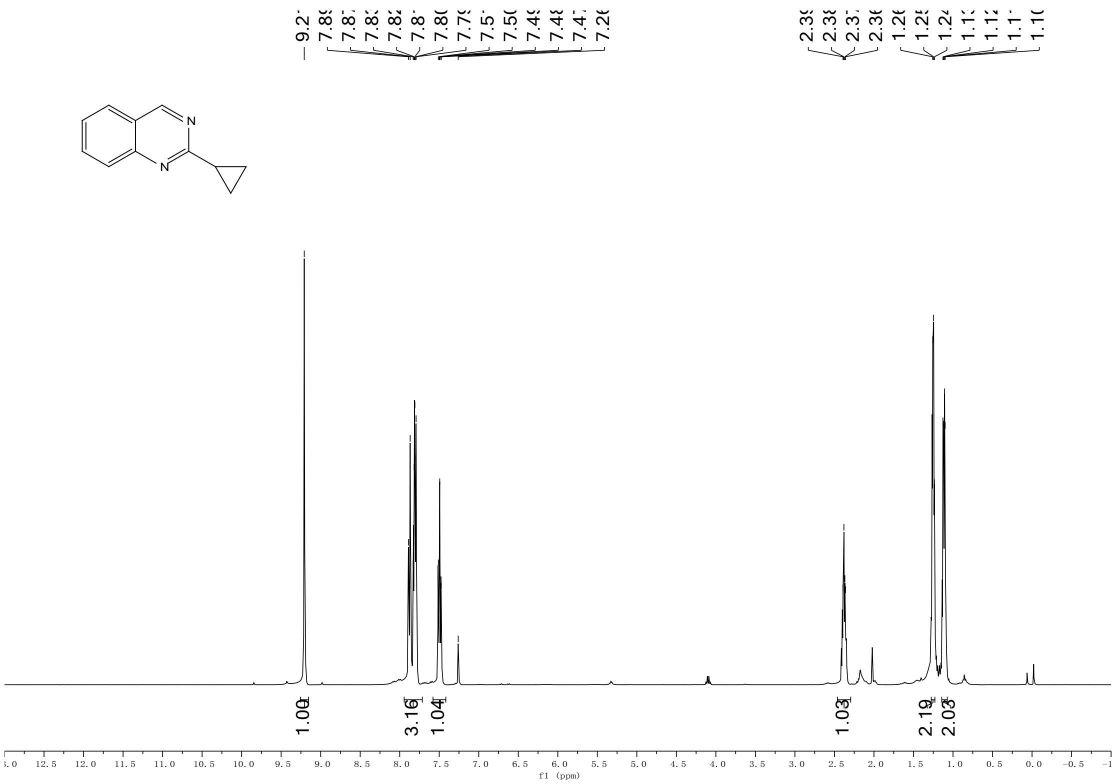
f1 (ppm)

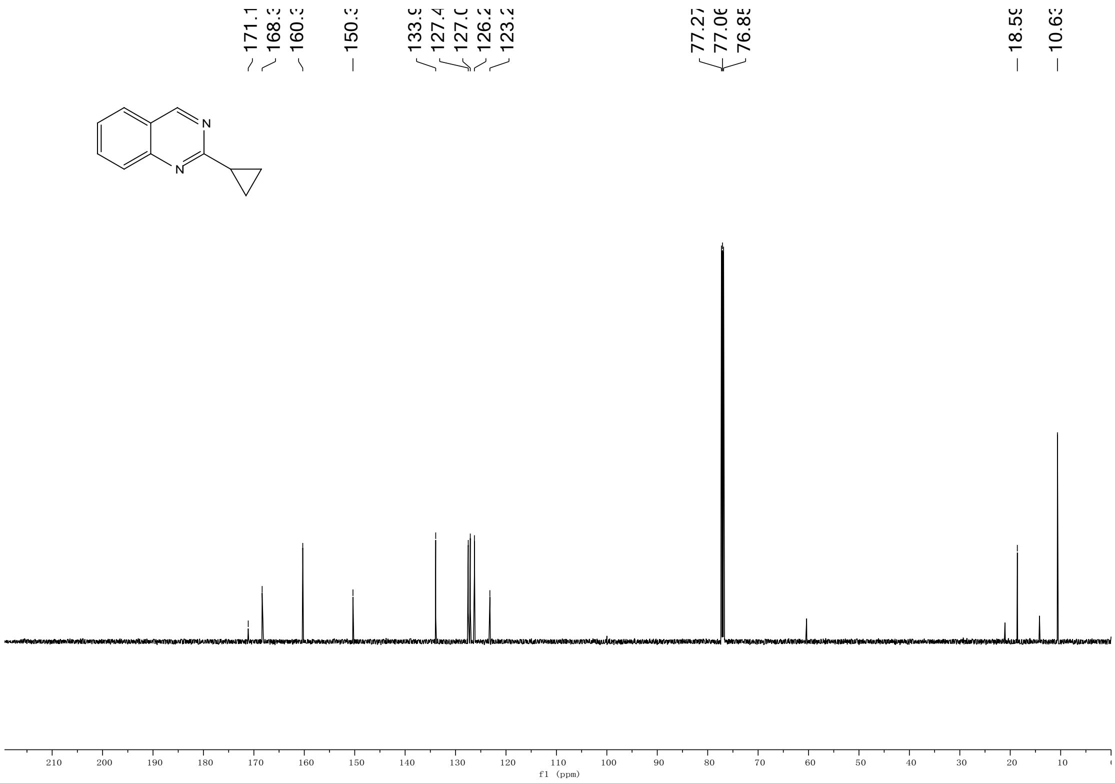
f1 (ppm)

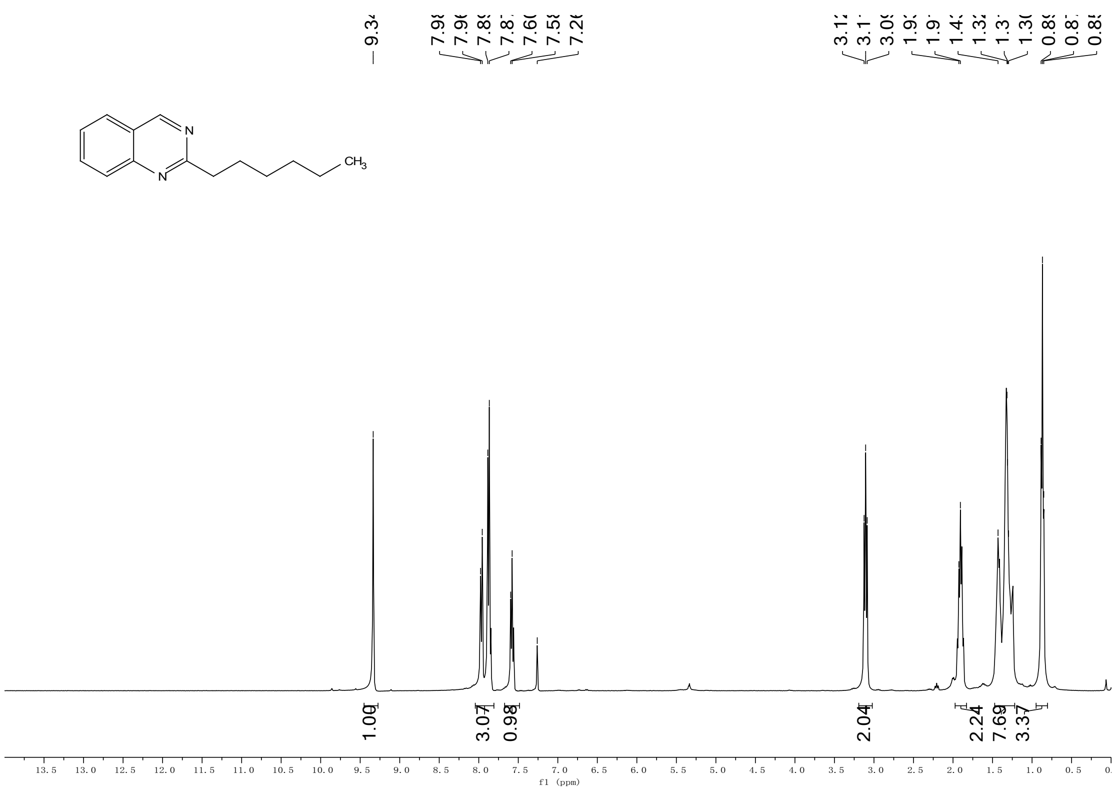
CH3
N
N
f1 (ppm)

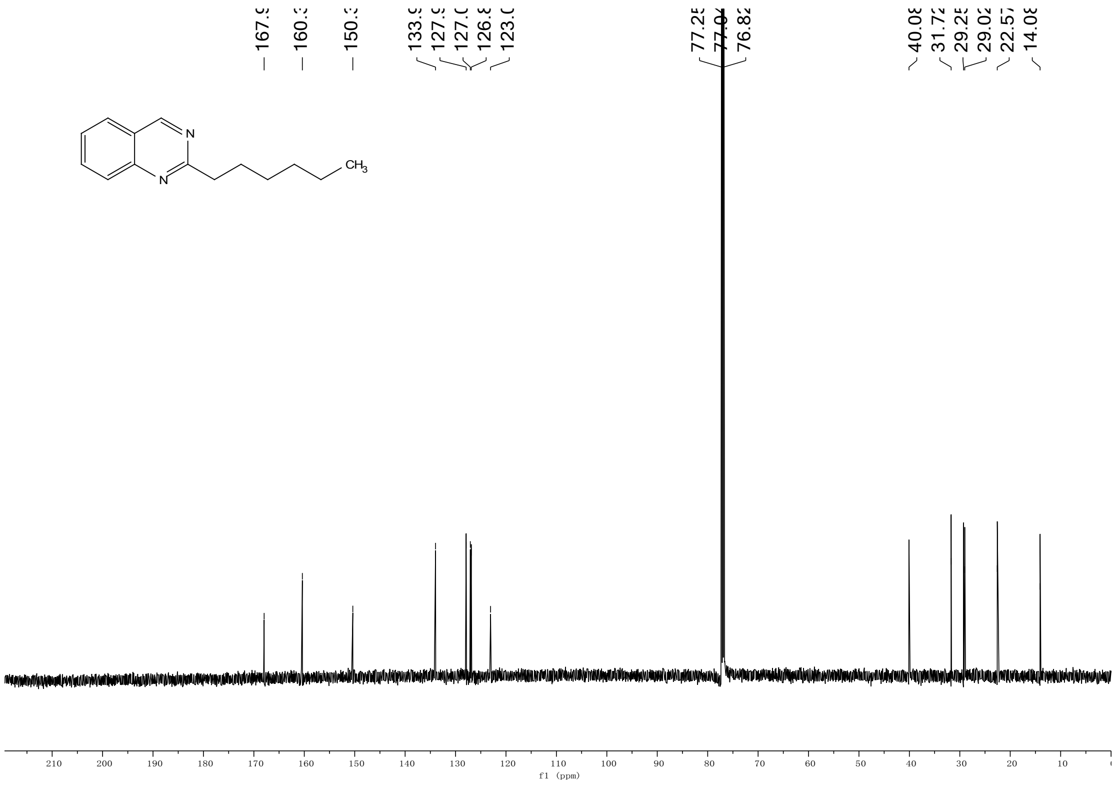
CH3
N
N
f1 (ppm)

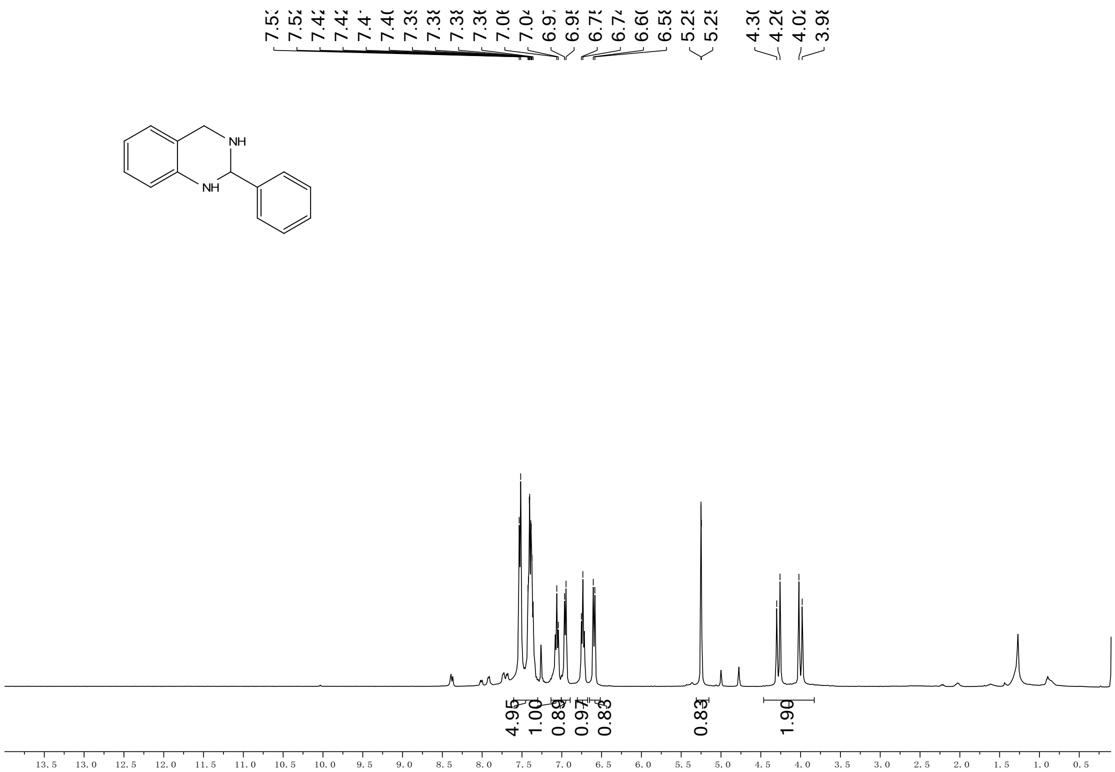
NH
NH
4.95
1.00
0.89
0.97
0.88
0.83
1.90
f1 (ppm)

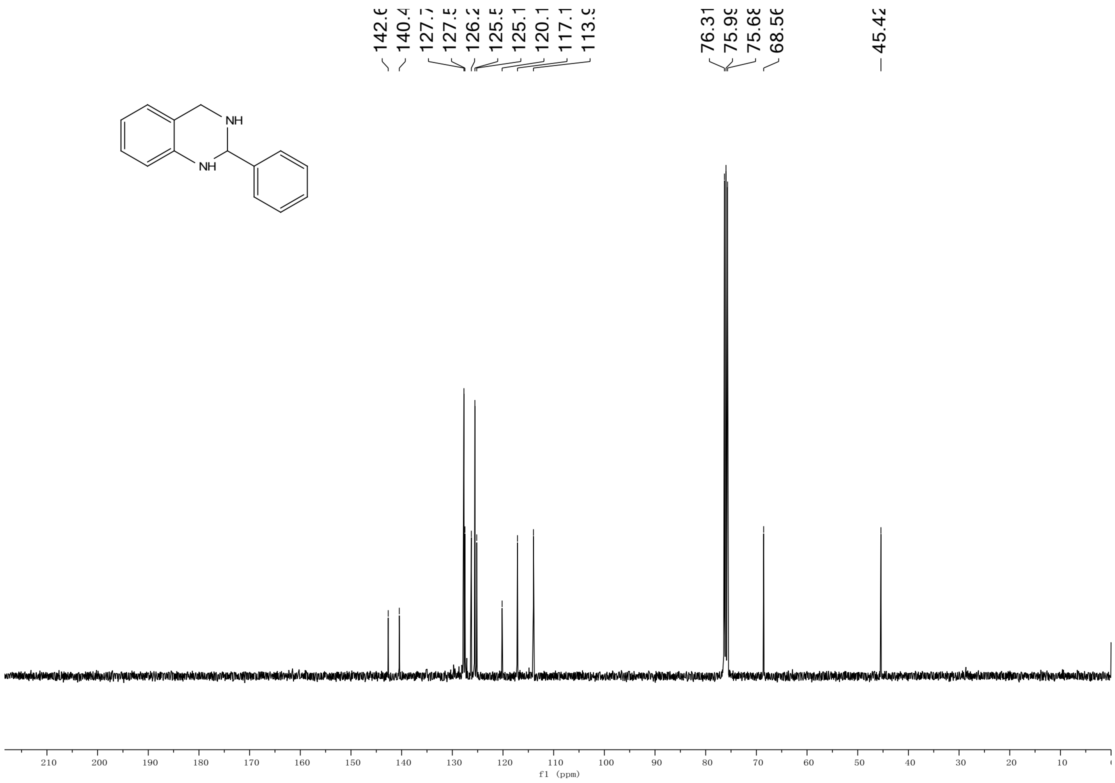
76.31
75.99
75.68
68.56
45.42
NH
NH
f1 (ppm)

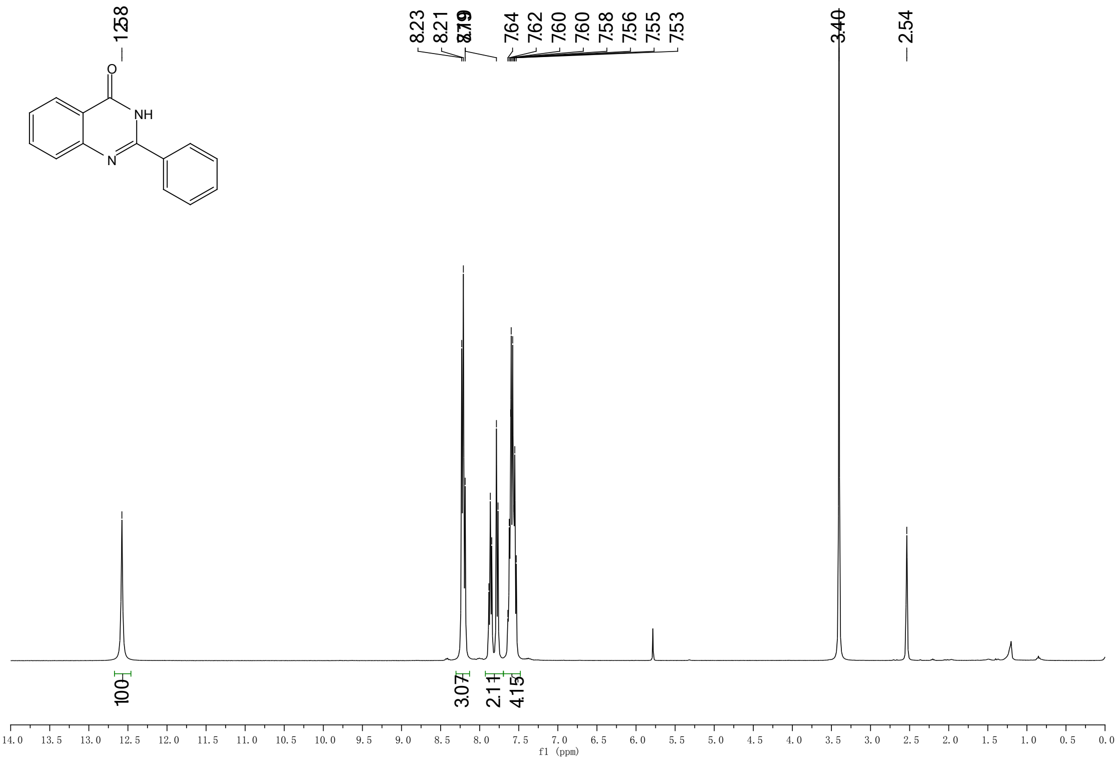

O
NH
N
8.23
8.21
7.64
7.62
7.60
7.60
7.58
7.56
7.55
7.53
2.54
3.07
2.11
14.0 13.5 13.0 12.5 12.0 11.5 11.0 10.5 10.0 9.5 9.0 8.5 8.0 7.5 7.0 6.5 6.0 5.5 5.0 4.5 4.0 3.5 3.0 2.5 2.0 1.5 1.0 0.5 0.0
f1 (ppm)

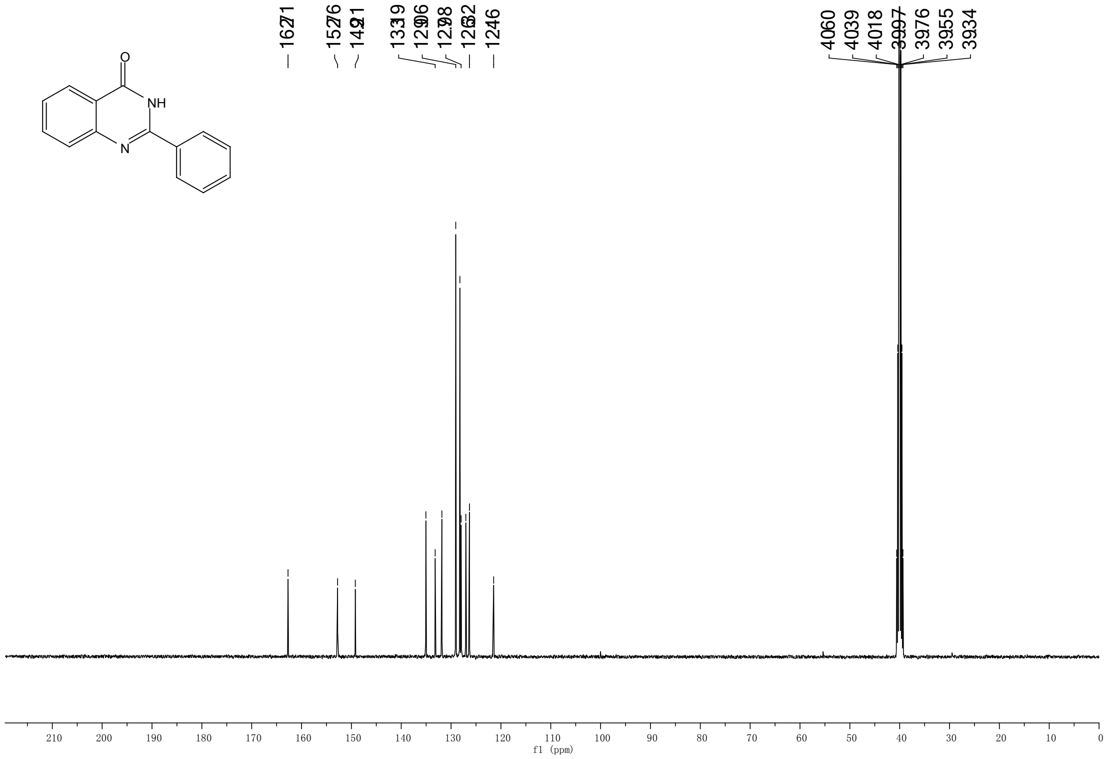

O
NH
N
124.6
40.60
40.39
40.18
39.76
39.55
39.34
210 200 190 180 170 160 150 140 130 120 110 100 90 80 70 60 50 40 30 20 10 0
f1 (ppm)

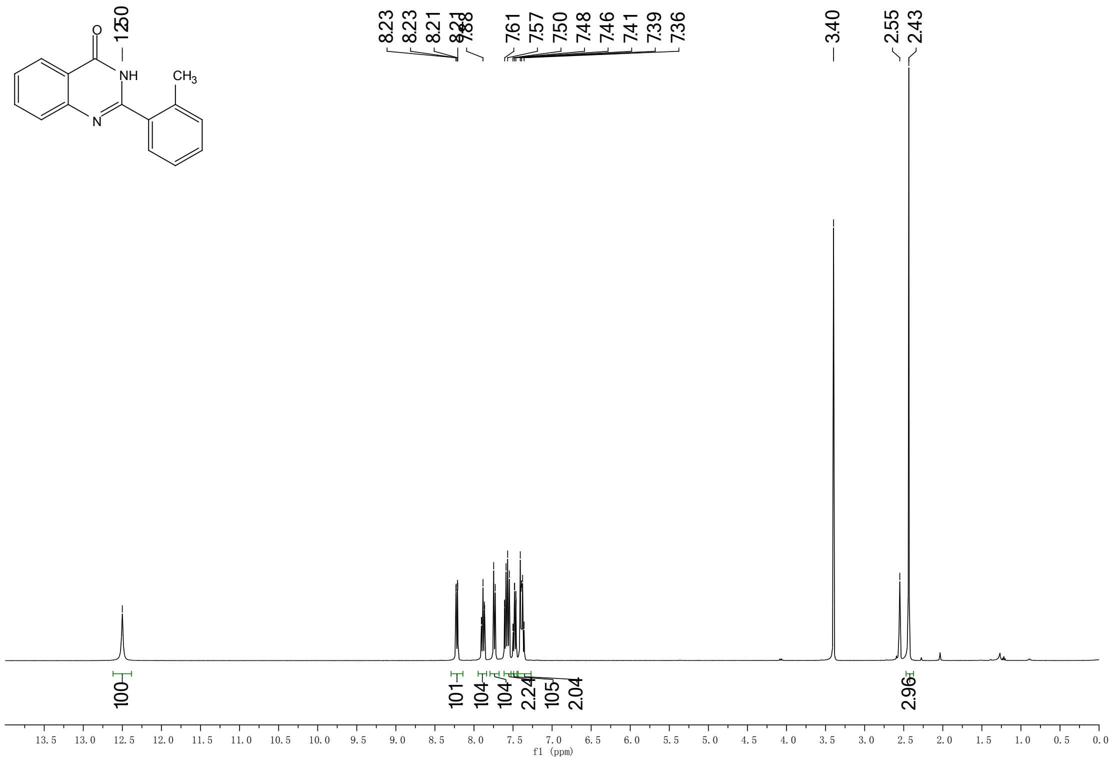
O
NH
CH3
N
8.23
8.23
8.21
7.61
7.57
7.50
7.48
7.46
7.41
7.39
7.36
3.40
2.55
2.43
2.24
2.04
2.96
f1 (ppm)

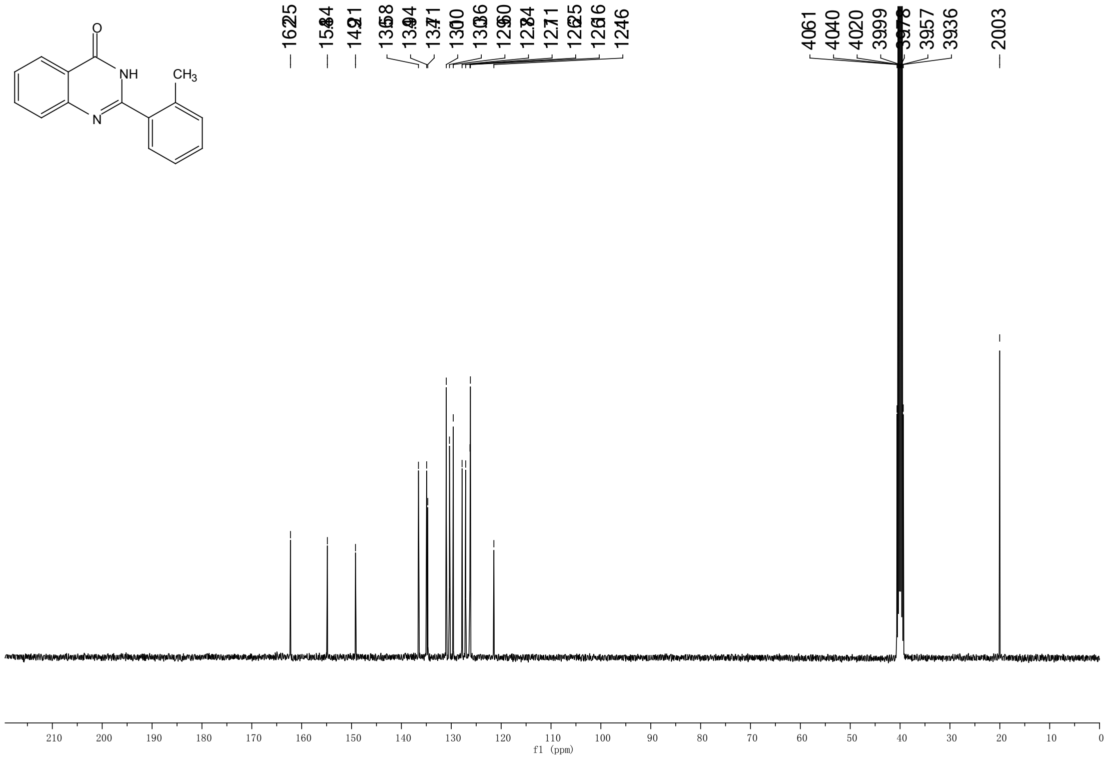
O
NH
CH3
N
40.61
40.40
40.20
39.99
39.57
39.36
f1 (ppm)

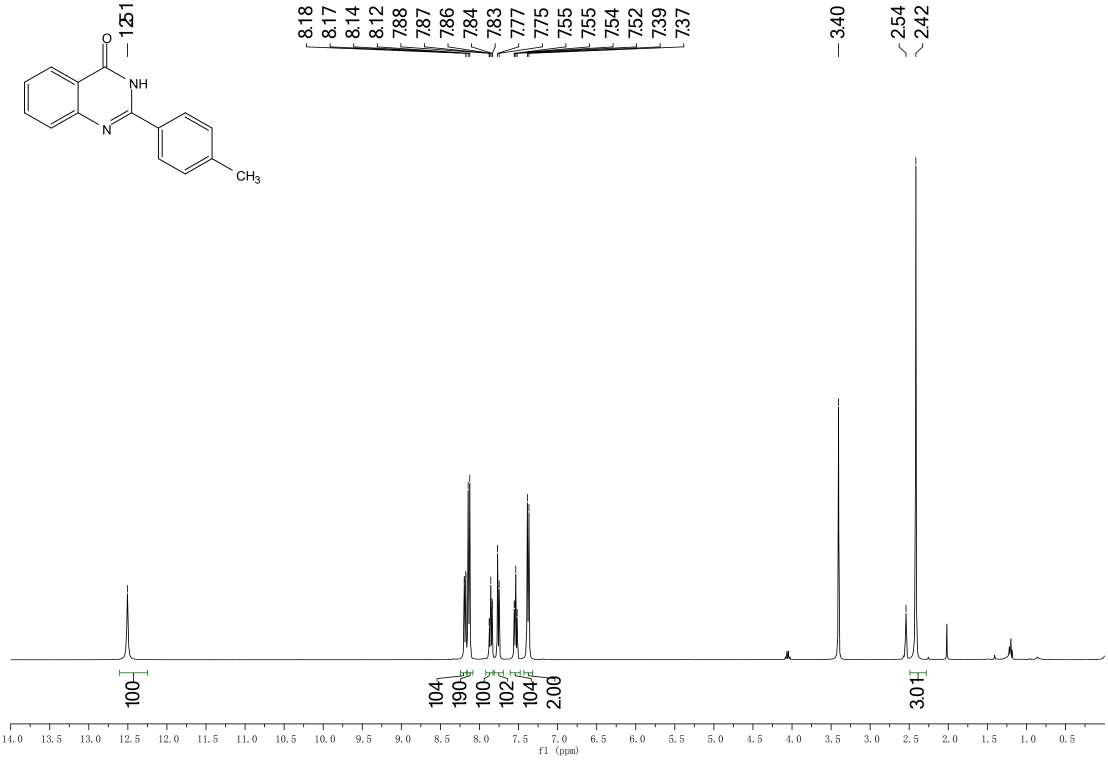
O
NH
N
CH3
8.18
8.17
8.14
8.12
7.88
7.87
7.86
7.84
7.83
7.77
7.75
7.55
7.55
7.54
7.52
7.39
7.37
3.40
2.54
2.42
2.00
3.01
f1 (ppm)

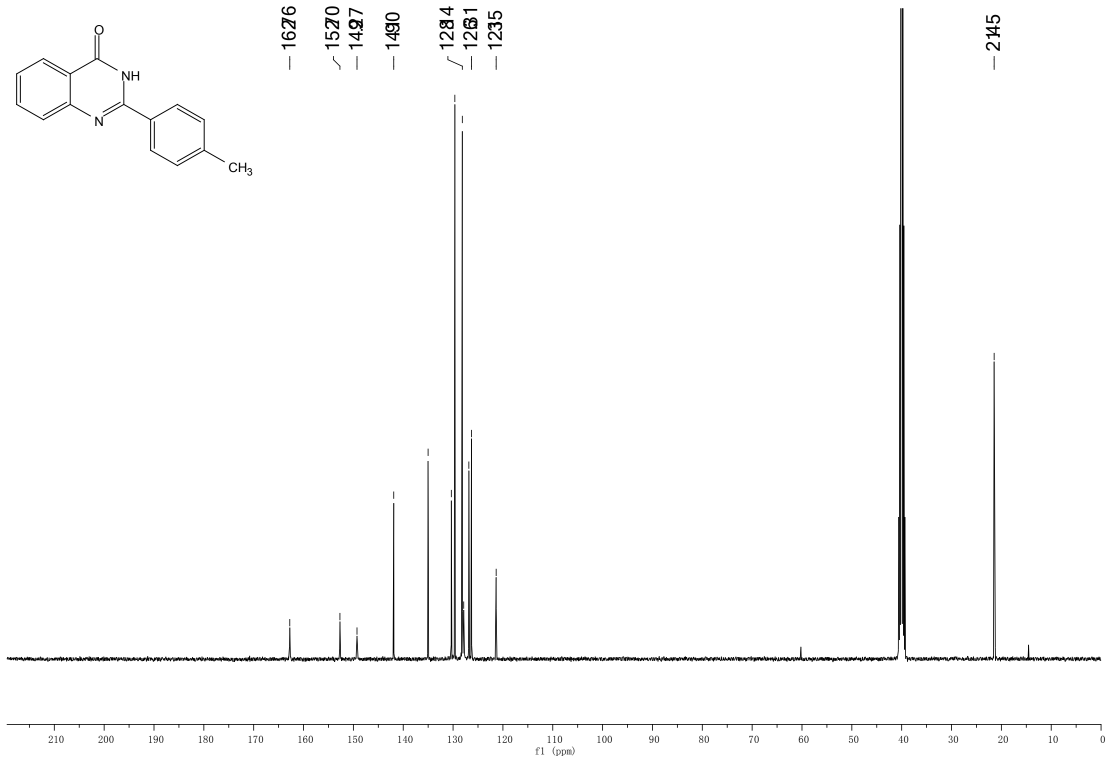
O
NH
N
CH3
f1 (ppm)

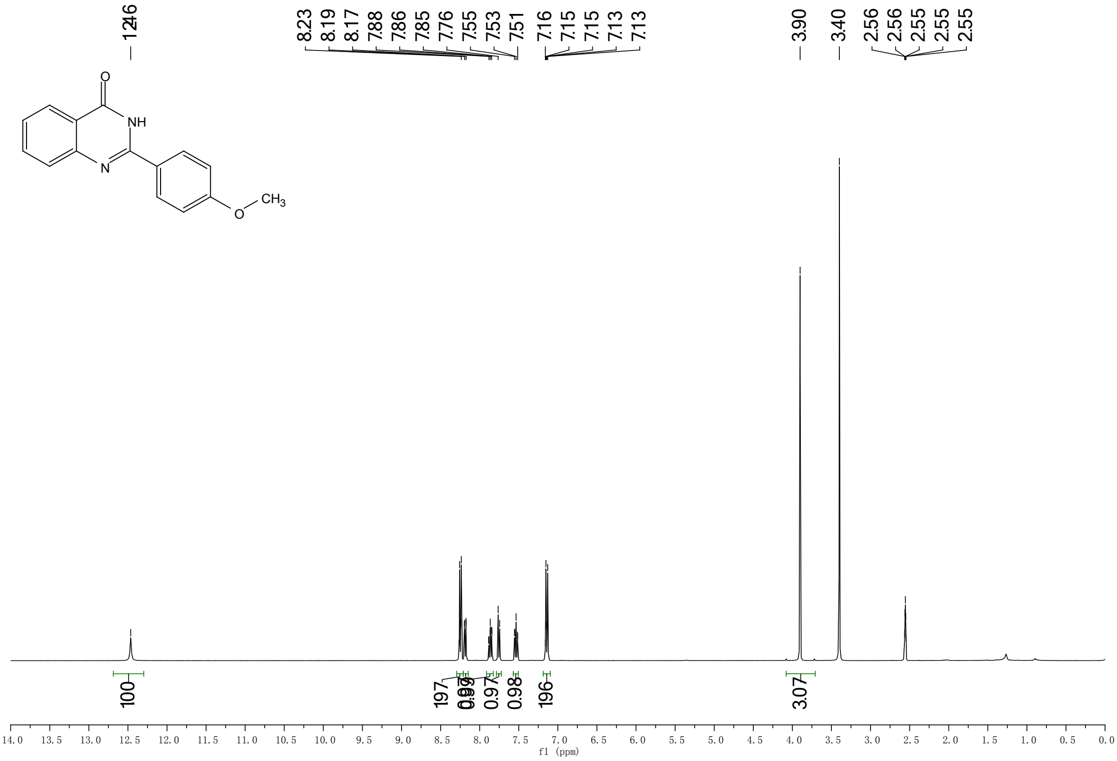

O
NH
N
O
CH3
8.23
8.19
8.17
7.88
7.86
7.85
7.76
7.55
7.53
7.51
7.16
7.15
7.15
7.13
7.13
3.90
3.40
2.56
2.56
2.55
2.55
2.55
1.00
0.97
0.98
3.07
14.0 13.5 13.0 12.5 12.0 11.5 11.0 10.5 10.0 9.5 9.0 8.5 8.0 7.5 7.0 6.5 6.0 5.5 5.0 4.5 4.0 3.5 3.0 2.5 2.0 1.5 1.0 0.5 0.0
f1 (ppm)

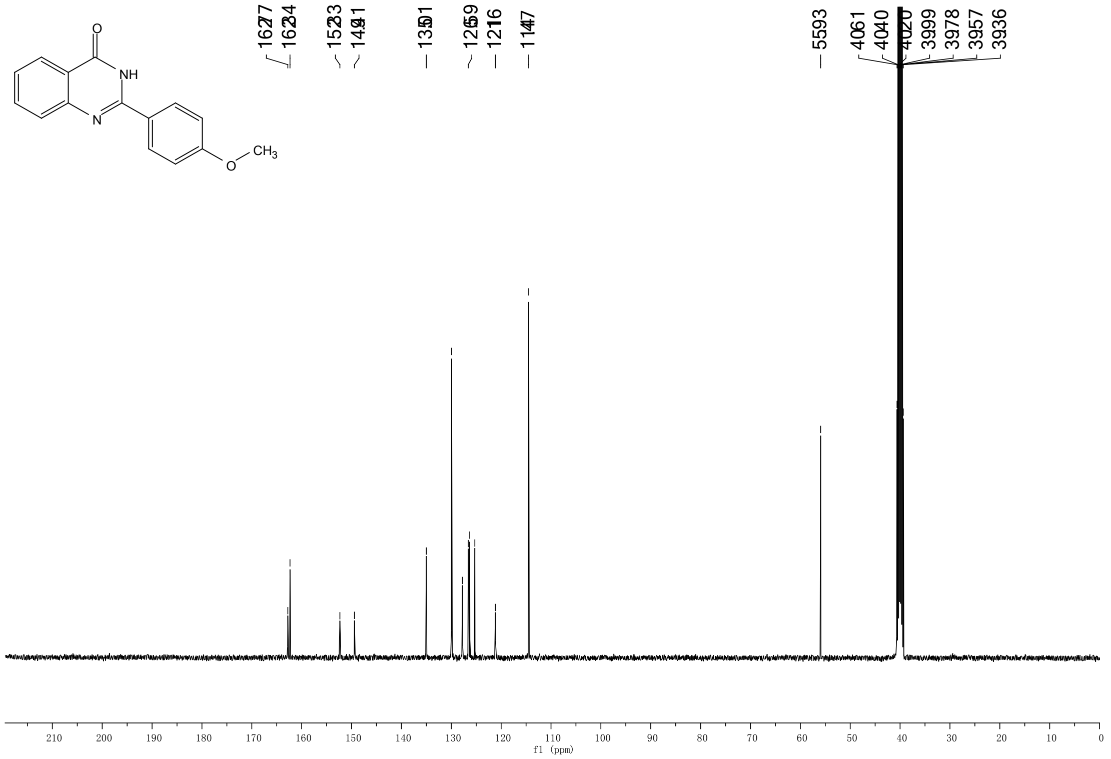

O
NH
N
O
CH3
55.93
40.61
40.40
40.20
39.99
39.78
39.57
39.36
210 200 190 180 170 160 150 140 130 120 110 100 90 80 70 60 50 40 30 20 10 0
f1 (ppm)

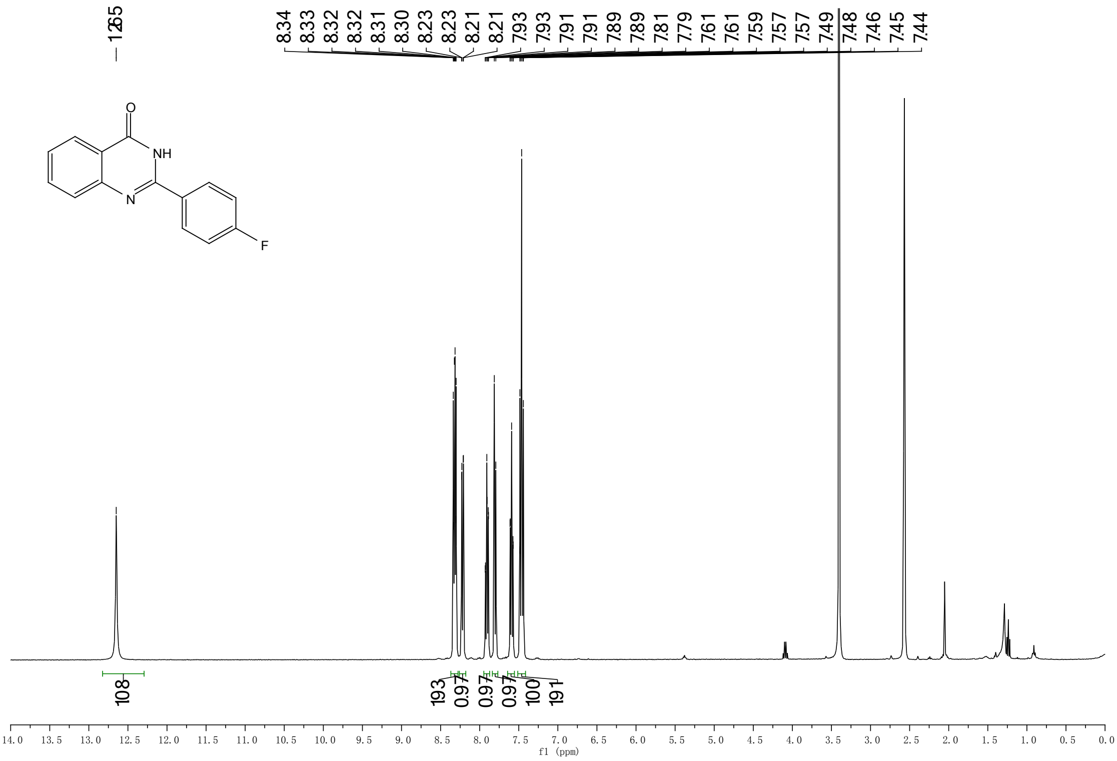

8.34
8.33
8.32
8.32
8.31
8.30
8.23
8.23
8.21
8.21
7.93
7.93
7.91
7.91
7.89
7.89
7.81
7.79
7.61
7.61
7.59
7.57
7.57
7.49
7.48
7.46
7.45
7.44
O
NH
N
F
f1 (ppm)

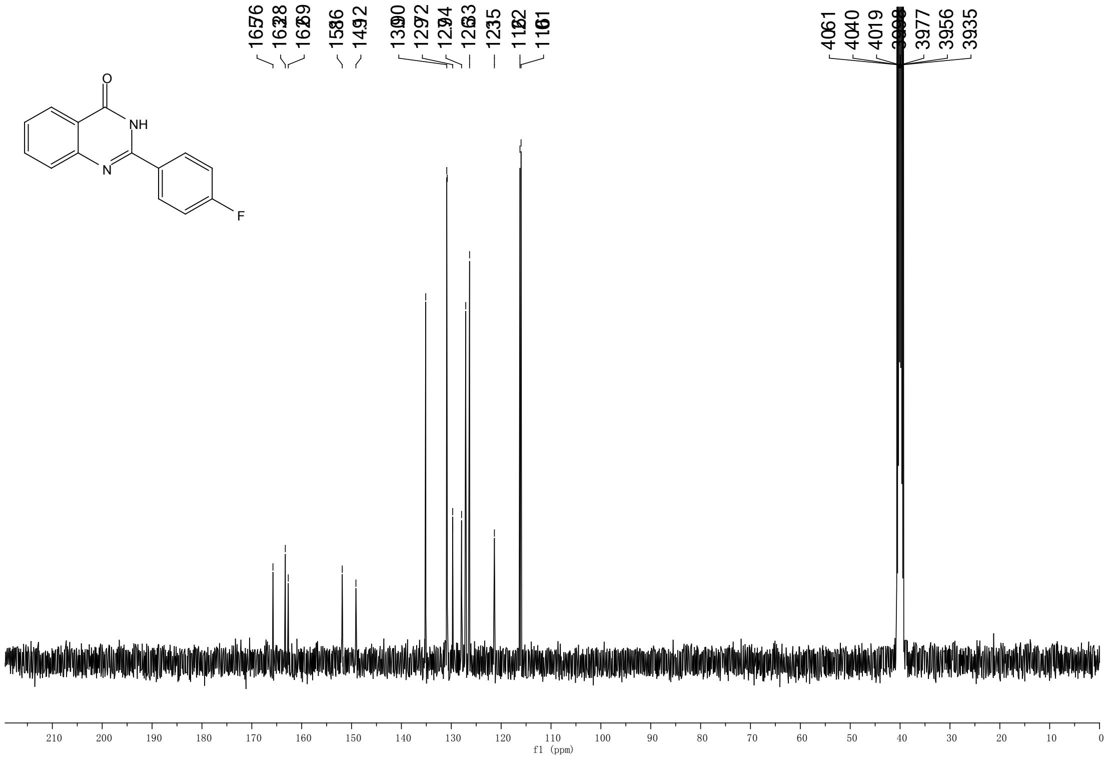

O
NH
N
F
f1 (ppm)

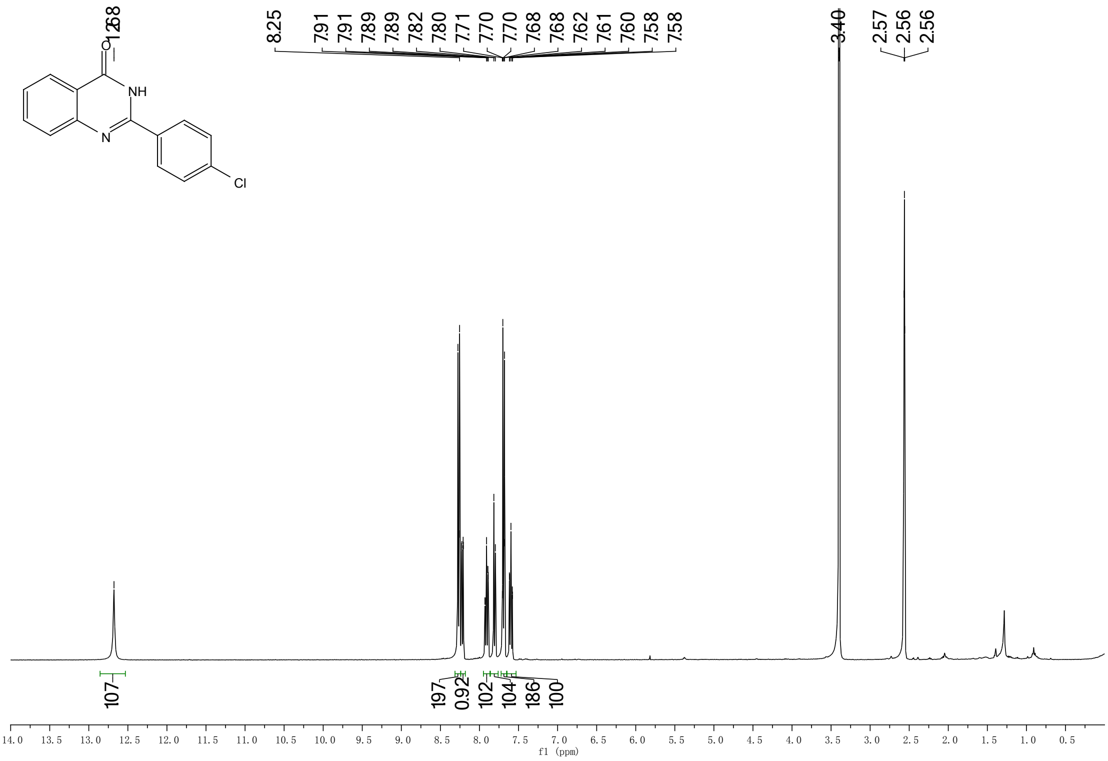

O
NH
N
Cl
8.25
7.91
7.91
7.89
7.89
7.82
7.80
7.71
7.70
7.70
7.68
7.68
7.62
7.61
7.60
7.58
7.58
2.57
2.56
2.56
0.92
f1 (ppm)

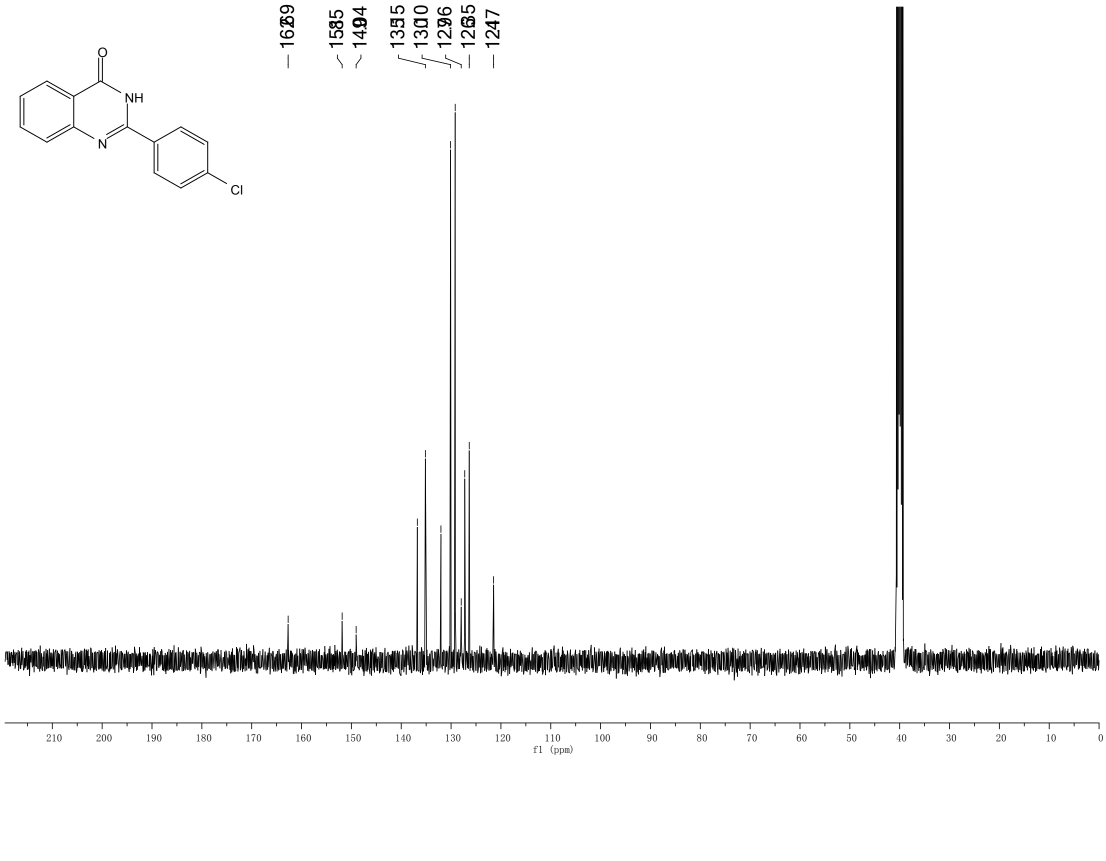

O
NH
N
Cl
f1 (ppm)

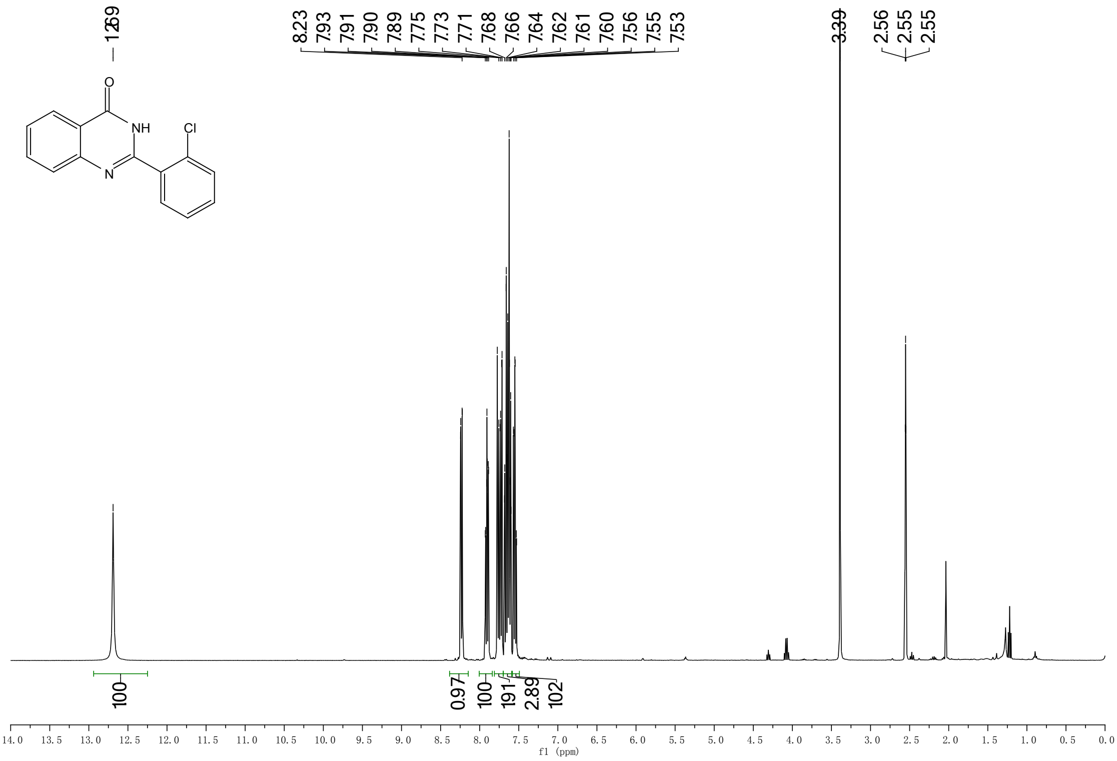
8.23
7.93
7.91
7.90
7.89
7.75
7.73
7.71
7.68
7.66
7.64
7.62
7.61
7.60
7.56
7.55
7.53
2.56
2.55
2.55
O
NH
Cl
N
0.97
2.89
f1 (ppm)

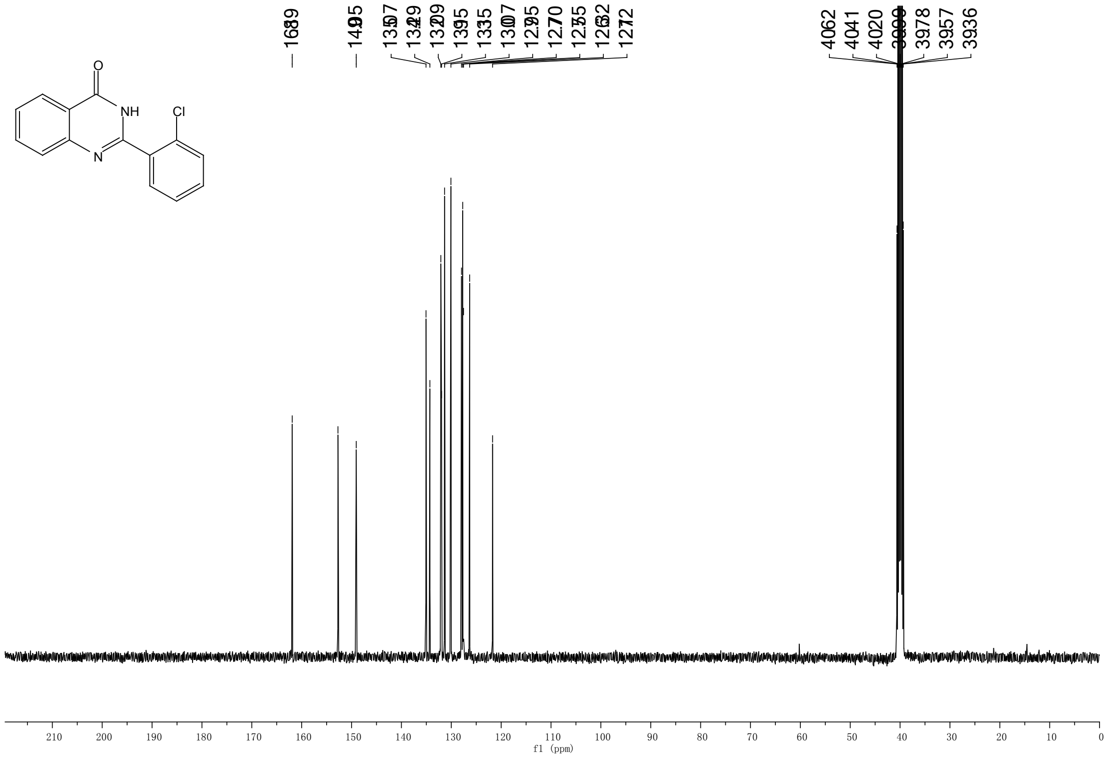
O
NH
Cl
N
f1 (ppm)

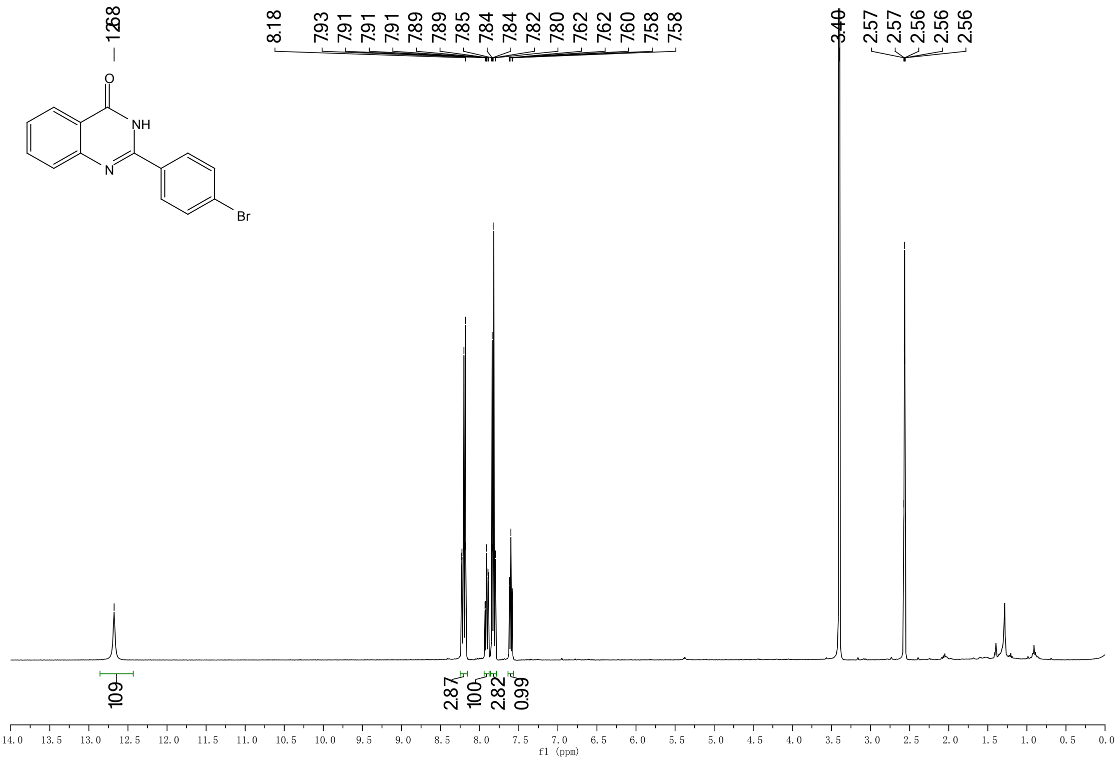
O
NH
N
Br
8.18
7.93
7.91
7.91
7.91
7.89
7.89
7.85
7.84
7.84
7.82
7.80
7.62
7.62
7.60
7.58
7.58
2.57
2.57
2.56
2.56
2.56
2.87
0.99
f1 (ppm)

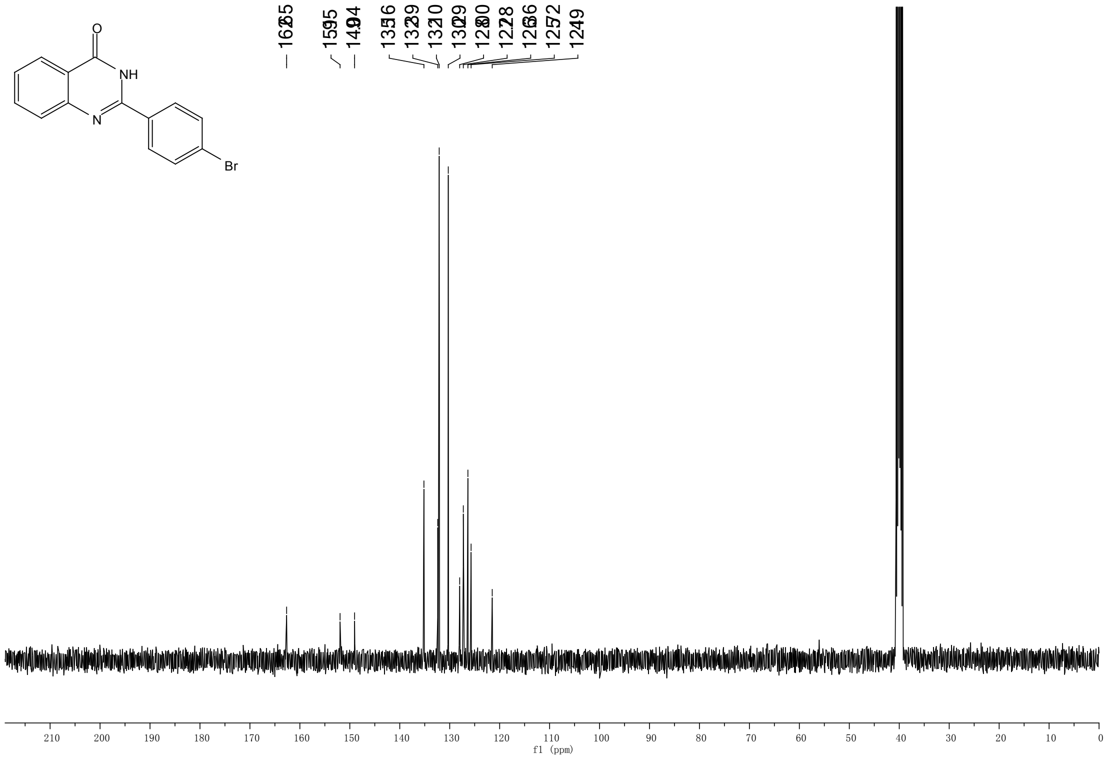
O
NH
N
Br
f1 (ppm)

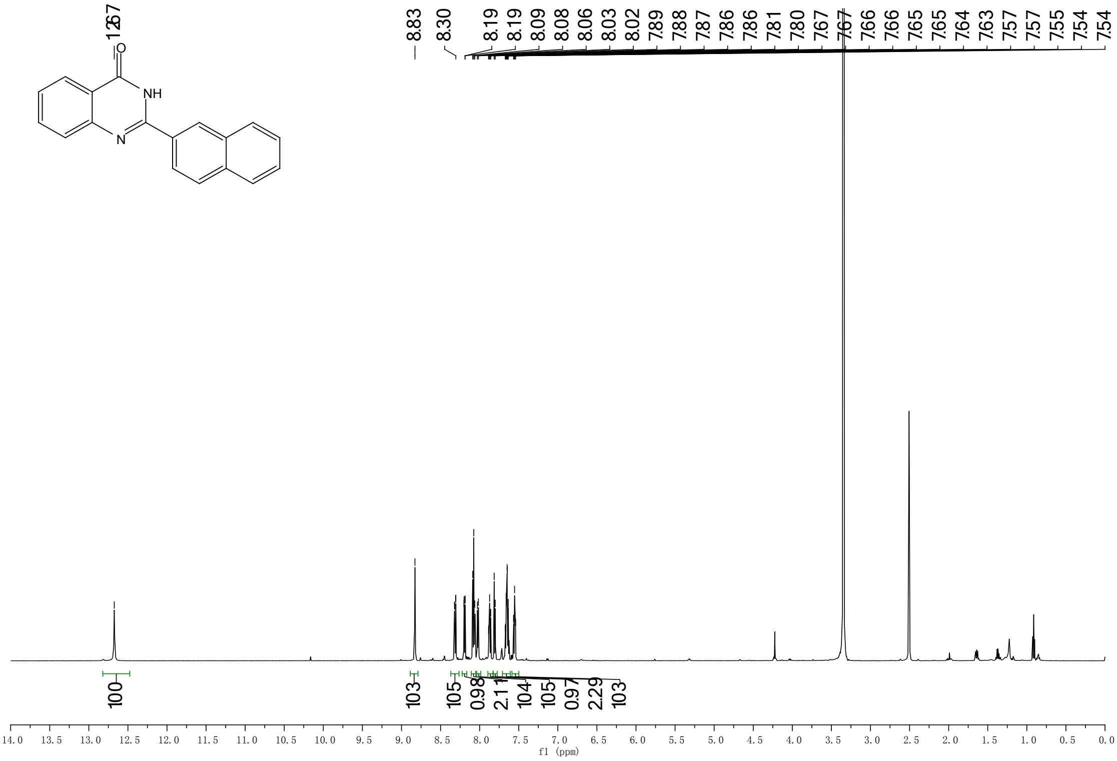

NH
N
O
8.83
8.30
8.19
8.19
8.09
8.08
8.06
8.03
8.02
7.89
7.88
7.87
7.86
7.86
7.81
7.80
7.67
7.67
7.66
7.66
7.65
7.65
7.64
7.63
7.57
7.57
7.55
7.54
7.54
0.98
2.11
0.97
2.29
f1 (ppm)

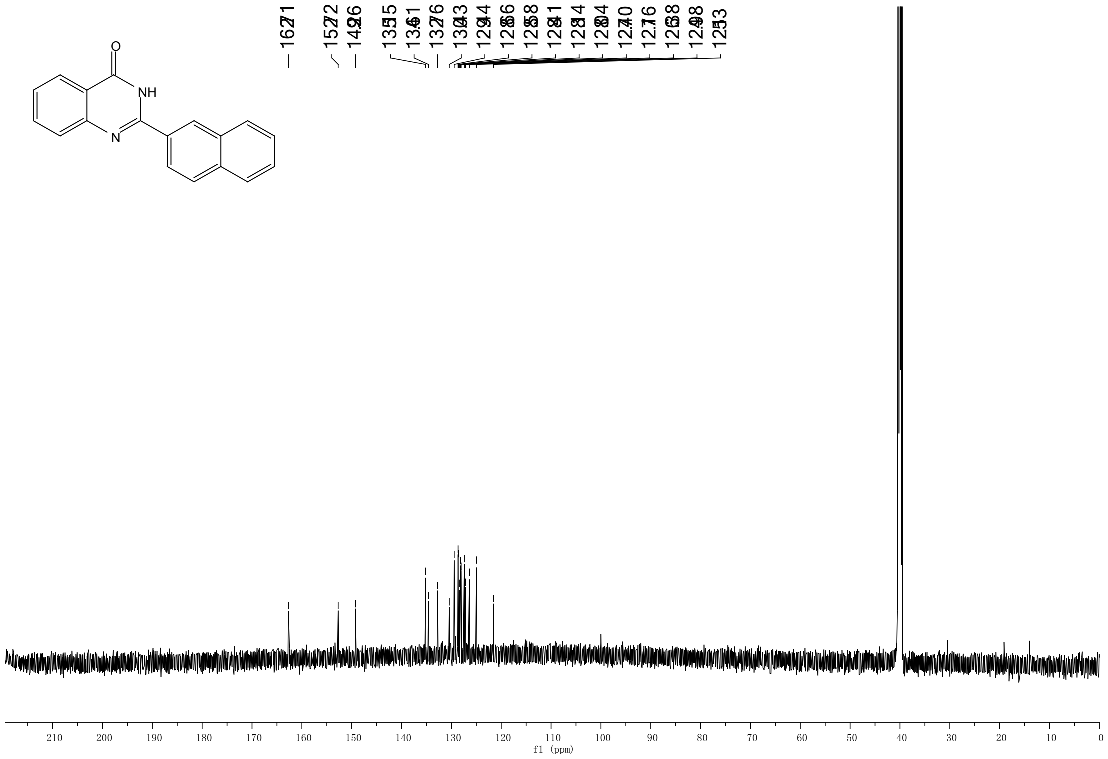

NH
N
O
f1 (ppm)

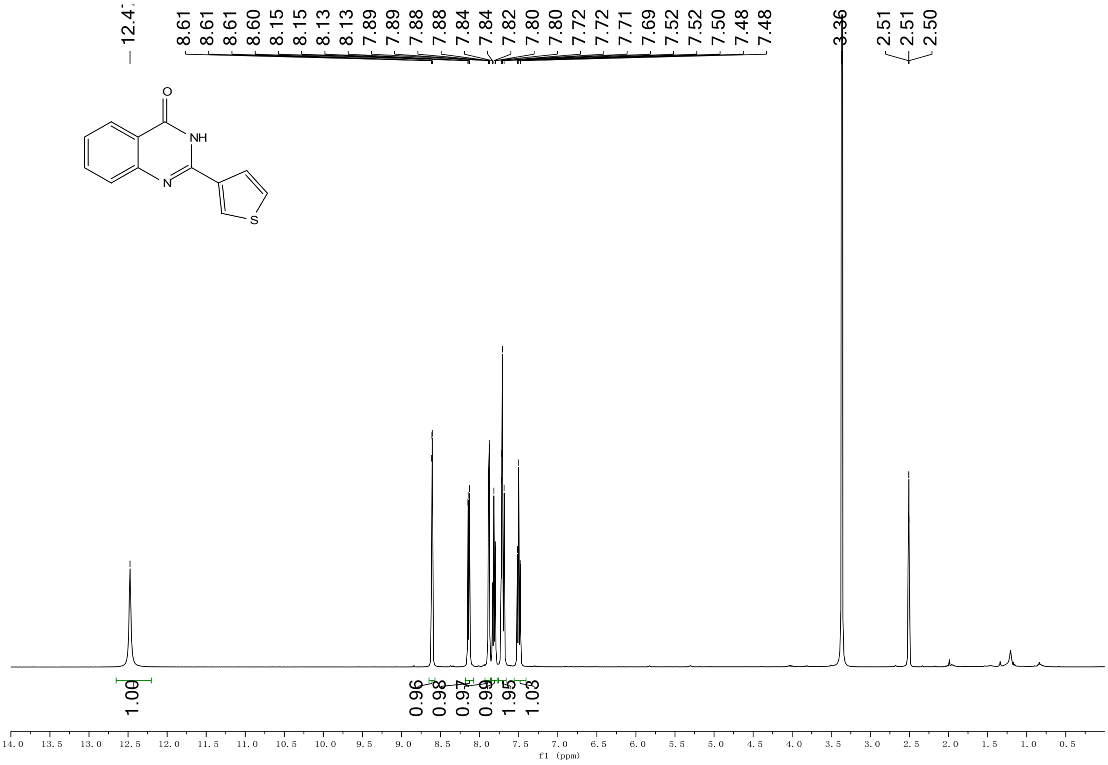
12.47
8.61 8.61 8.61 8.60 8.15 8.15 8.13 8.13 7.89 7.89 7.88 7.88 7.84 7.84 7.82 7.80 7.80 7.72 7.72 7.71 7.69 7.52 7.52 7.50 7.48 7.48
3.36
2.51 2.51 2.50
O
NH
N
S
1.00
0.96 0.98 0.97 0.99 1.95 1.03
14.0 13.5 13.0 12.5 12.0 11.5 11.0 10.5 10.0 9.5 9.0 8.5 8.0 7.5 7.0 6.5 6.0 5.5 5.0 4.5 4.0 3.5 3.0 2.5 2.0 1.5 1.0 0.5
f1 (ppm)

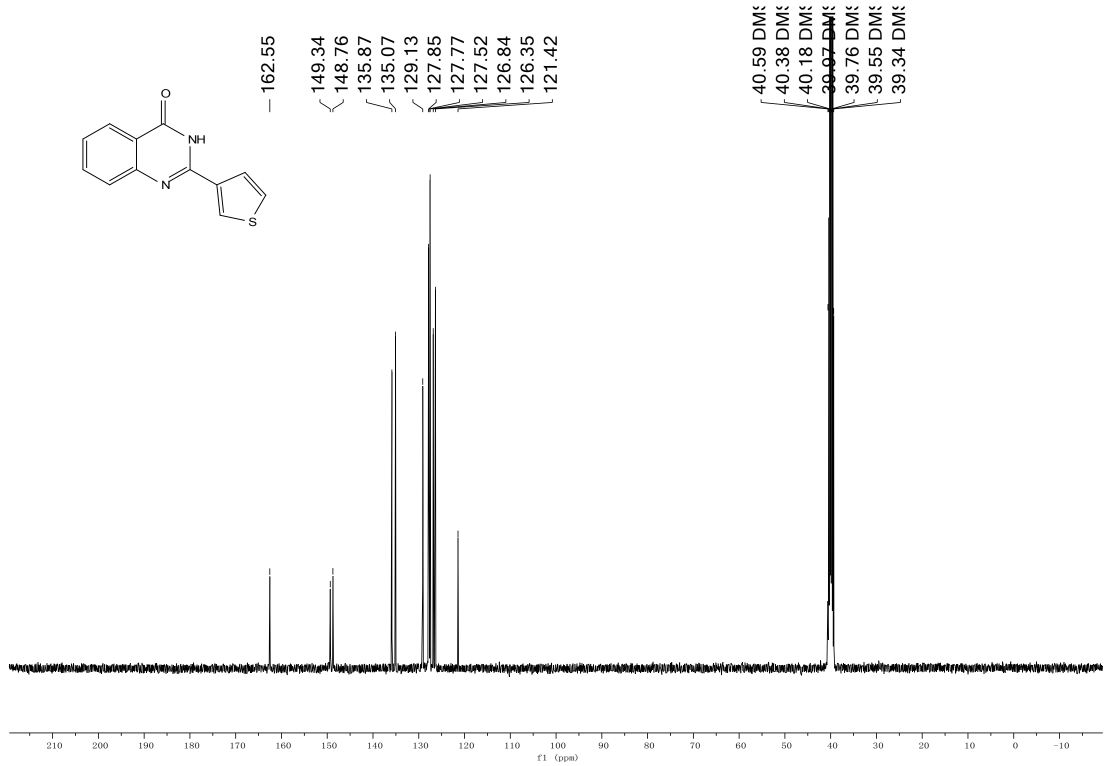
162.55
149.34 148.76 135.87 135.07 129.13 127.85 127.77 127.52 126.84 126.35 121.42
40.59 DMSO 40.38 DMSO 40.18 DMSO 39.97 DMSO 39.76 DMSO 39.55 DMSO 39.34 DMSO
O
NH
N
S
210 200 190 180 170 160 150 140 130 120 110 100 90 80 70 60 50 40 30 20 10 0 -10
f1 (ppm)

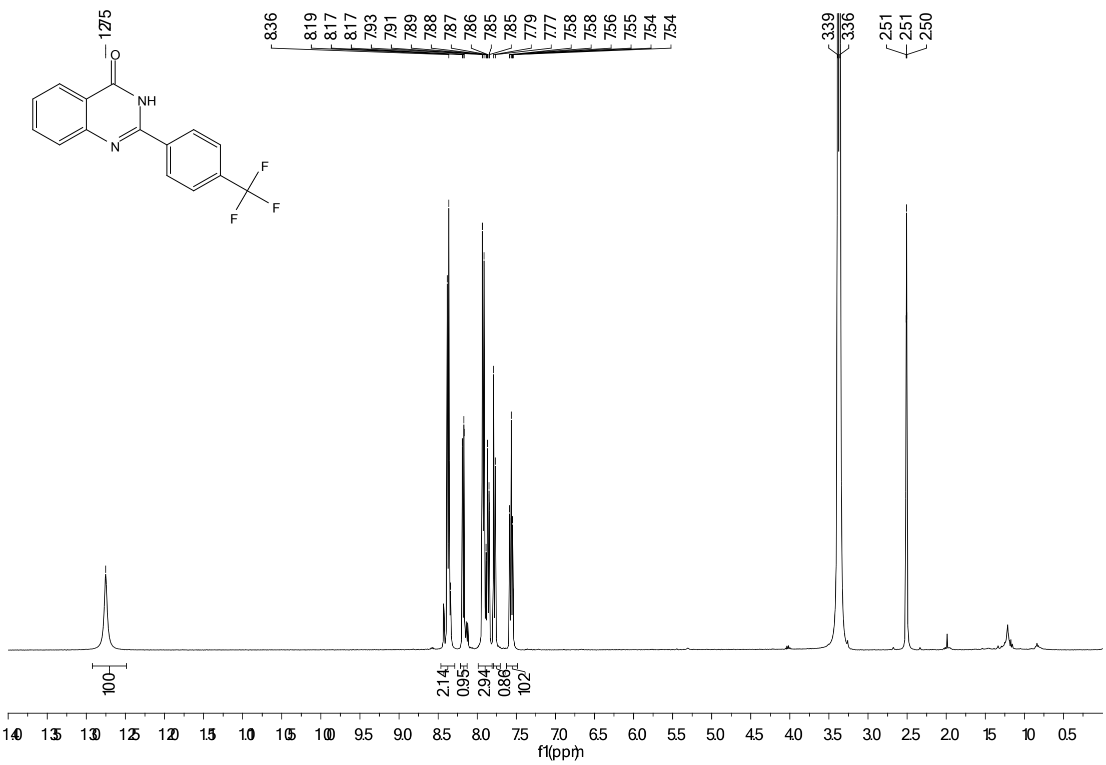

O
NH
N
F
F
F
8.36
8.19
8.17
8.17
7.93
7.91
7.89
7.88
7.87
7.86
7.85
7.85
7.79
7.77
7.58
7.58
7.56
7.55
7.54
7.54
3.39
3.36
2.51
2.51
2.50
2.14
0.95
2.94
0.86
f1 (ppm)

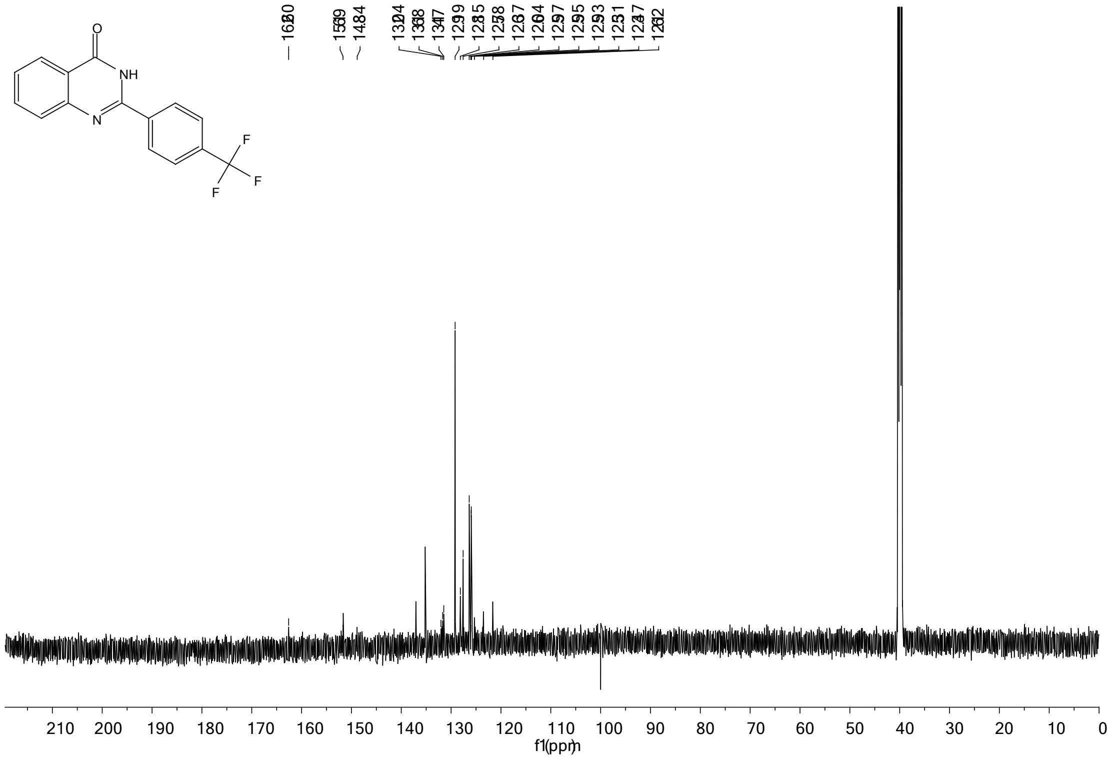

O
NH
N
F
F
F
14.84
f1 (ppm)

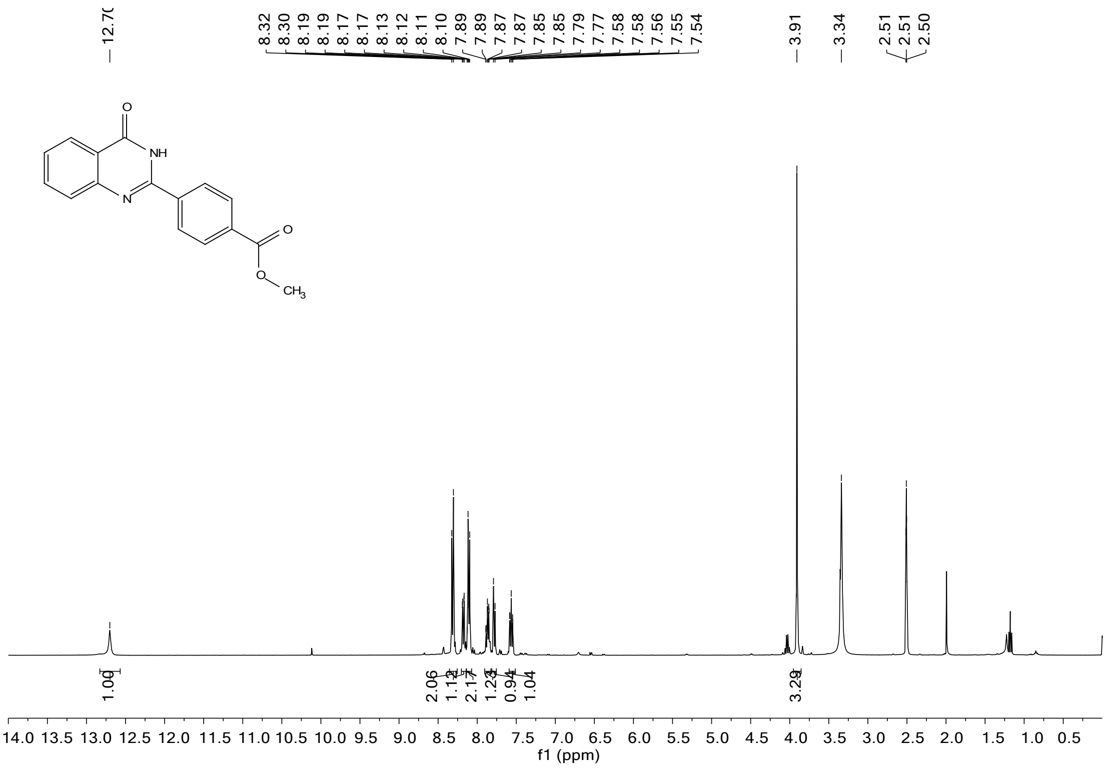
12.7
8.32 8.30 8.19 8.19 8.17 8.17 8.13 8.12 8.11 8.10 7.89 7.89 7.87 7.87 7.85 7.85 7.79 7.77 7.58 7.58 7.56 7.55 7.54
3.91
3.34
2.51 2.51 2.50
O
NH
N
O
O
CH3
1.00
2.06 1.12 2.17 1.24 0.94 1.04
3.29
14.0 13.5 13.0 12.5 12.0 11.5 11.0 10.5 10.0 9.5 9.0 8.5 8.0 7.5 7.0 6.5 6.0 5.5 5.0 4.5 4.0 3.5 3.0 2.5 2.0 1.5 1.0 0.5
f1 (ppm)

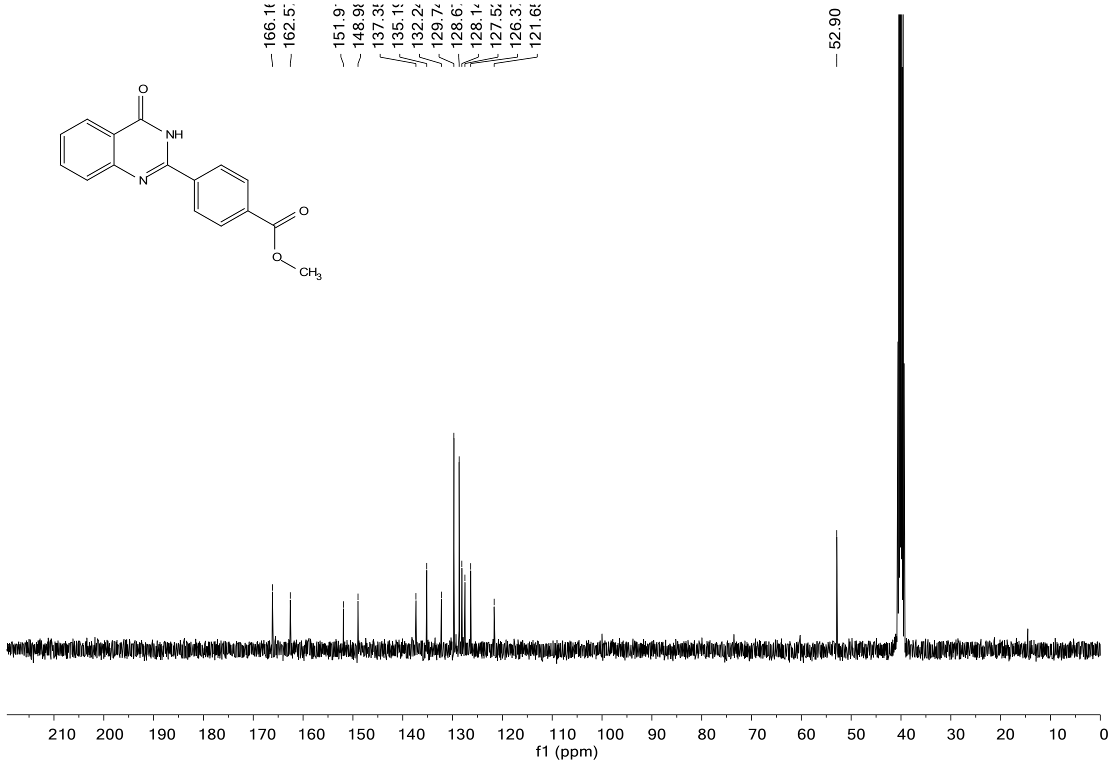
166.1
162.5
151.9
148.9
137.3
135.1
132.2
129.7
128.6
128.1
127.5
126.3
121.6
52.90
O
NH
N
O
O
CH3
210 200 190 180 170 160 150 140 130 120 110 100 90 80 70 60 50 40 30 20 10 0
f1 (ppm)

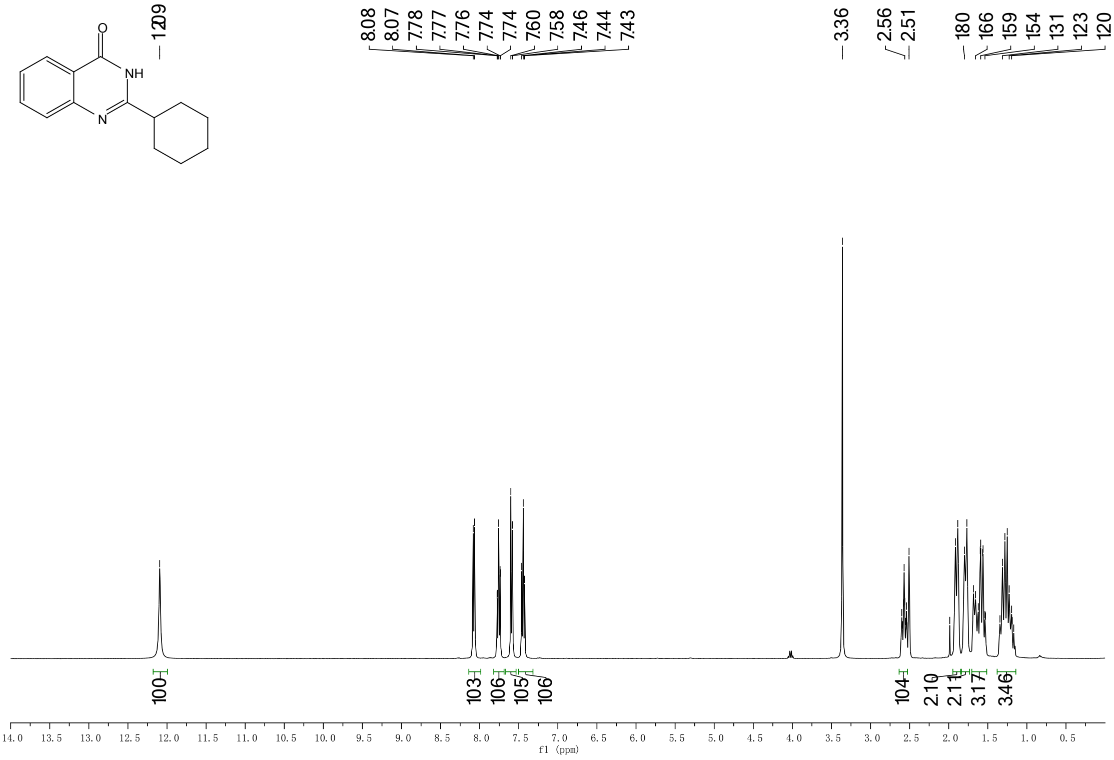
O
NH
N
f1 (ppm)

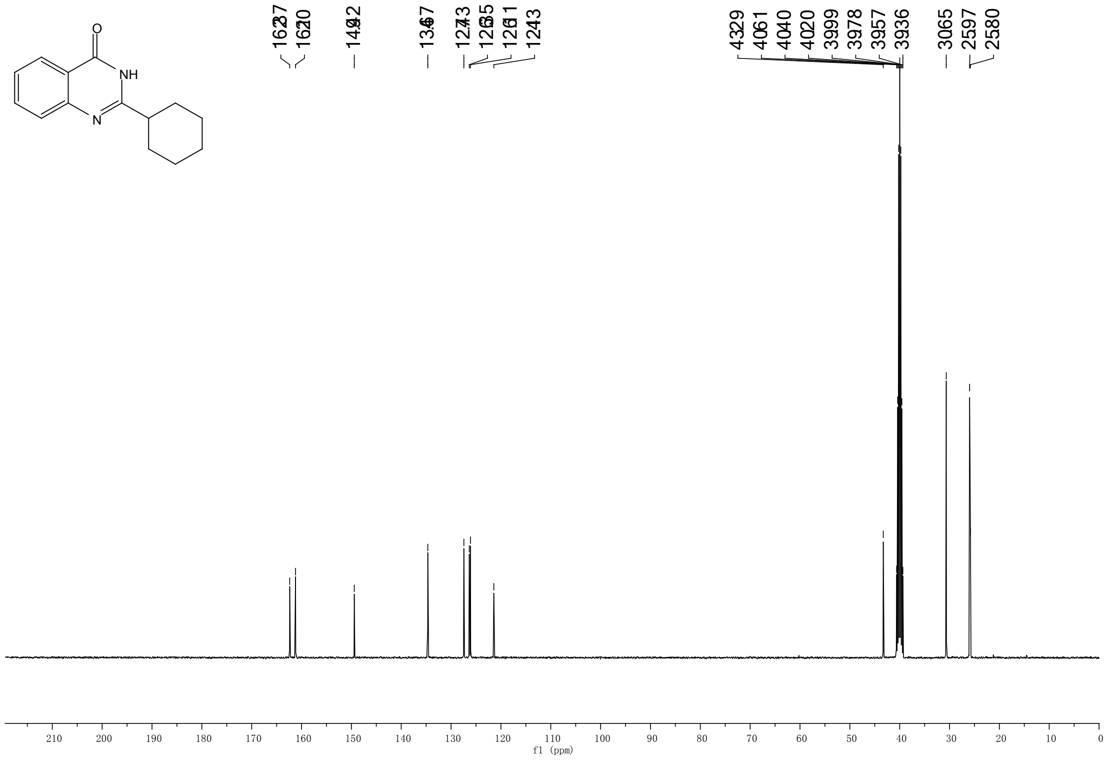
O
NH
N
f1 (ppm)

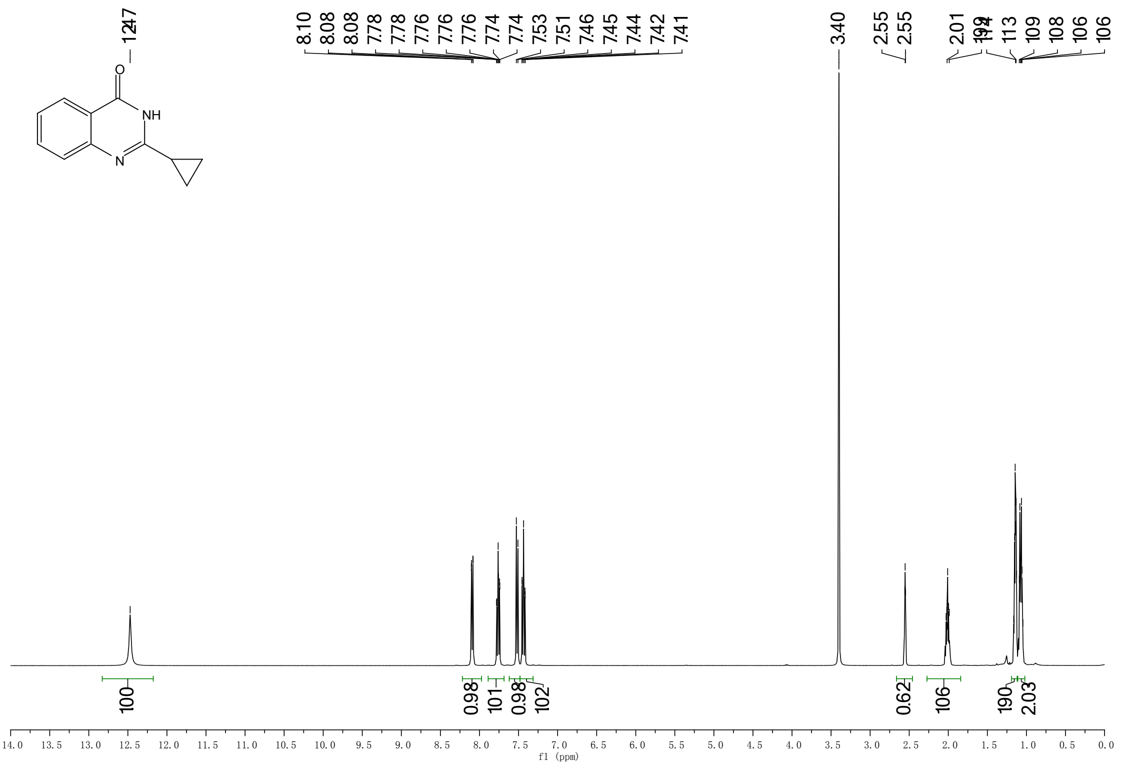
O
NH
N
8.10 8.08 8.08 7.78 7.78 7.76 7.76 7.74 7.74 7.53 7.51 7.46 7.45 7.44 7.42 7.41
3.40
2.55 2.55
2.01
0.98 0.98 2.03
f1 (ppm)

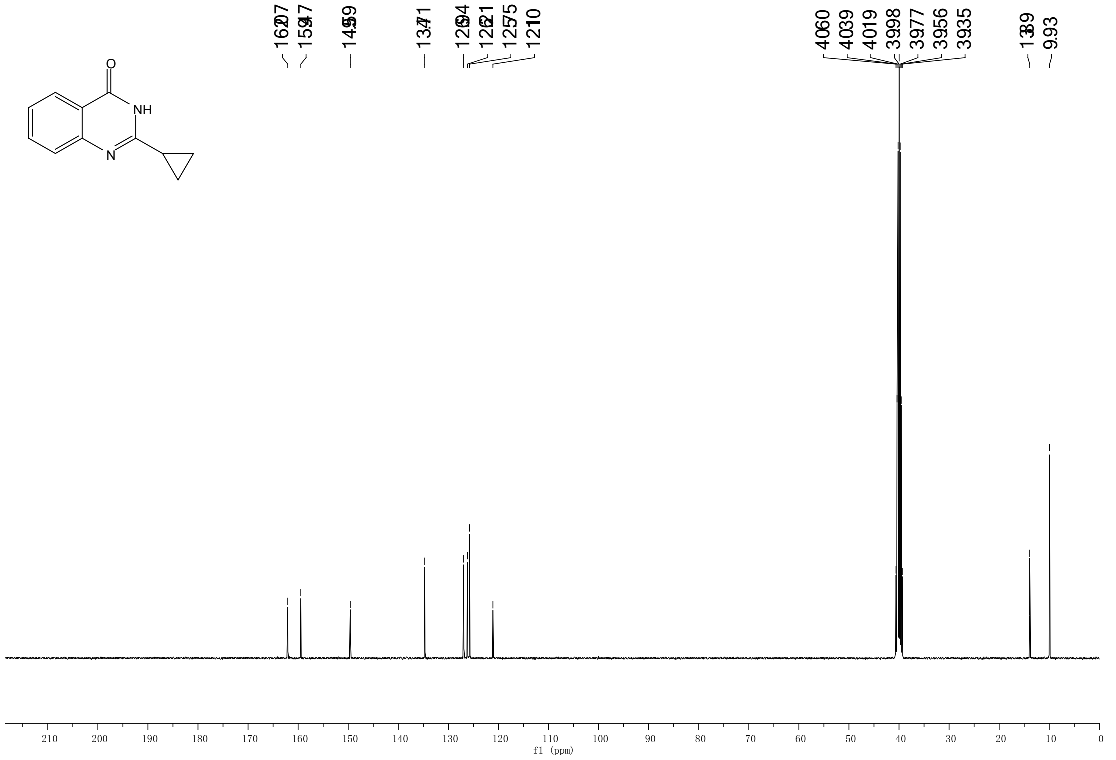
O
NH
N
9.93
f1 (ppm)

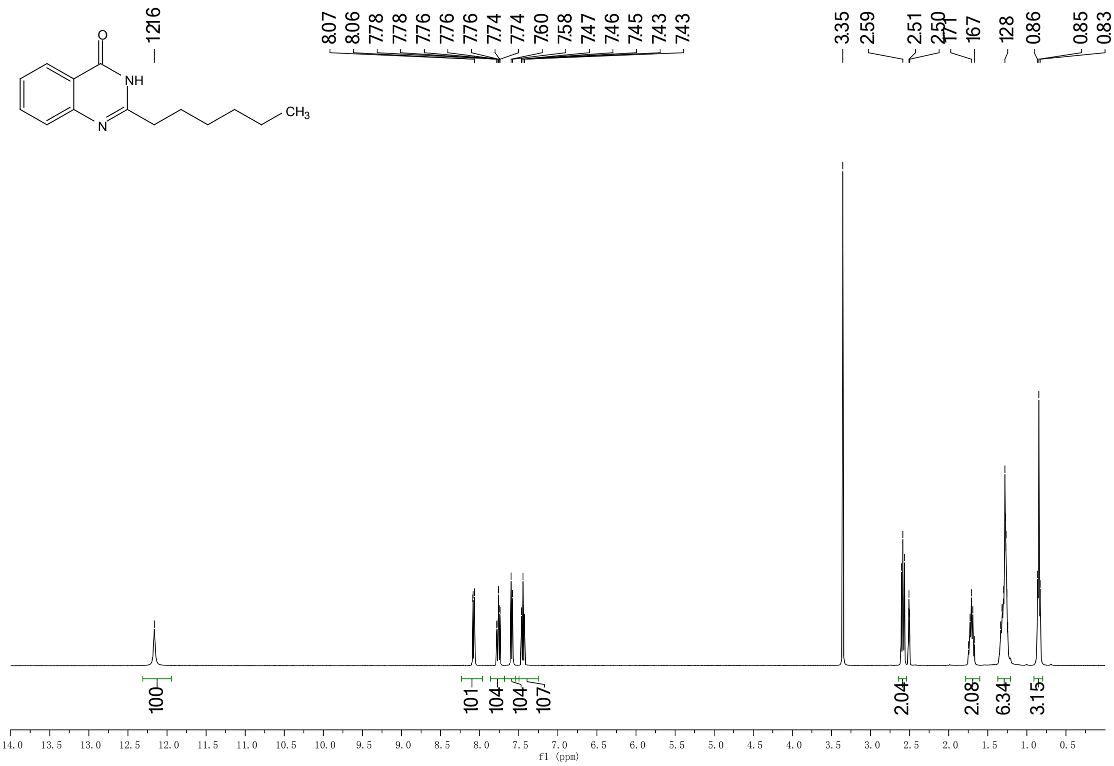
O
NH
N
CH3
f1 (ppm)

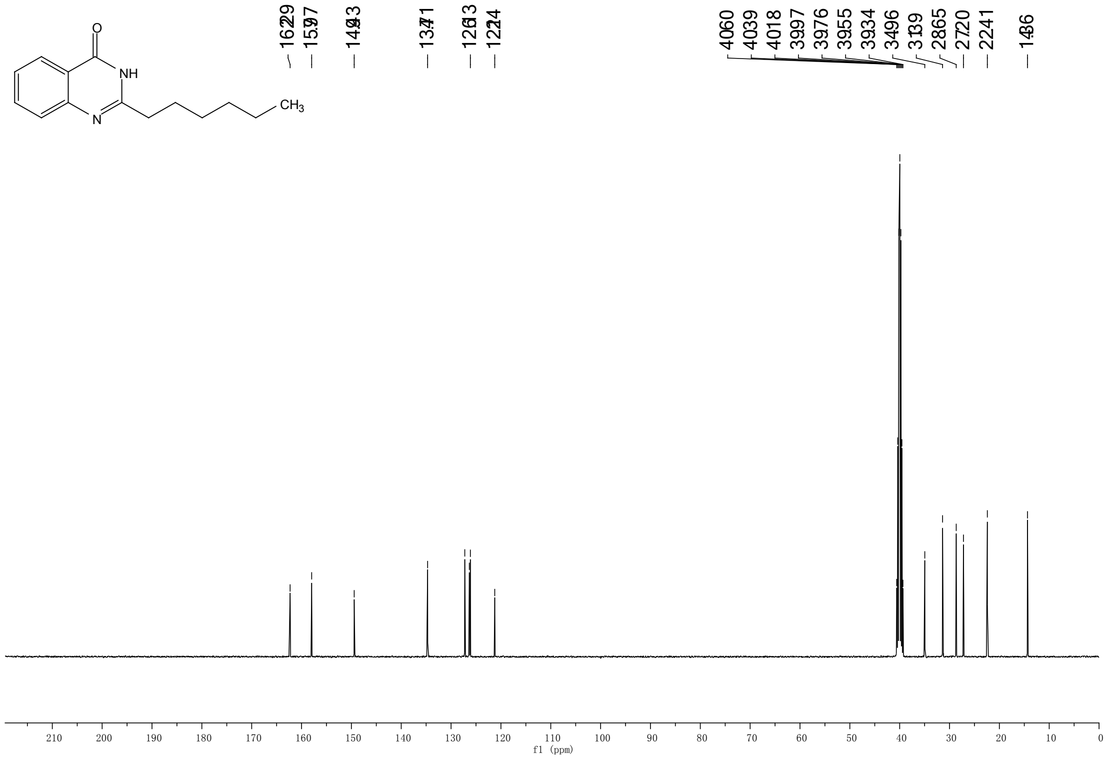
O
NH
N
CH3
f1 (ppm)

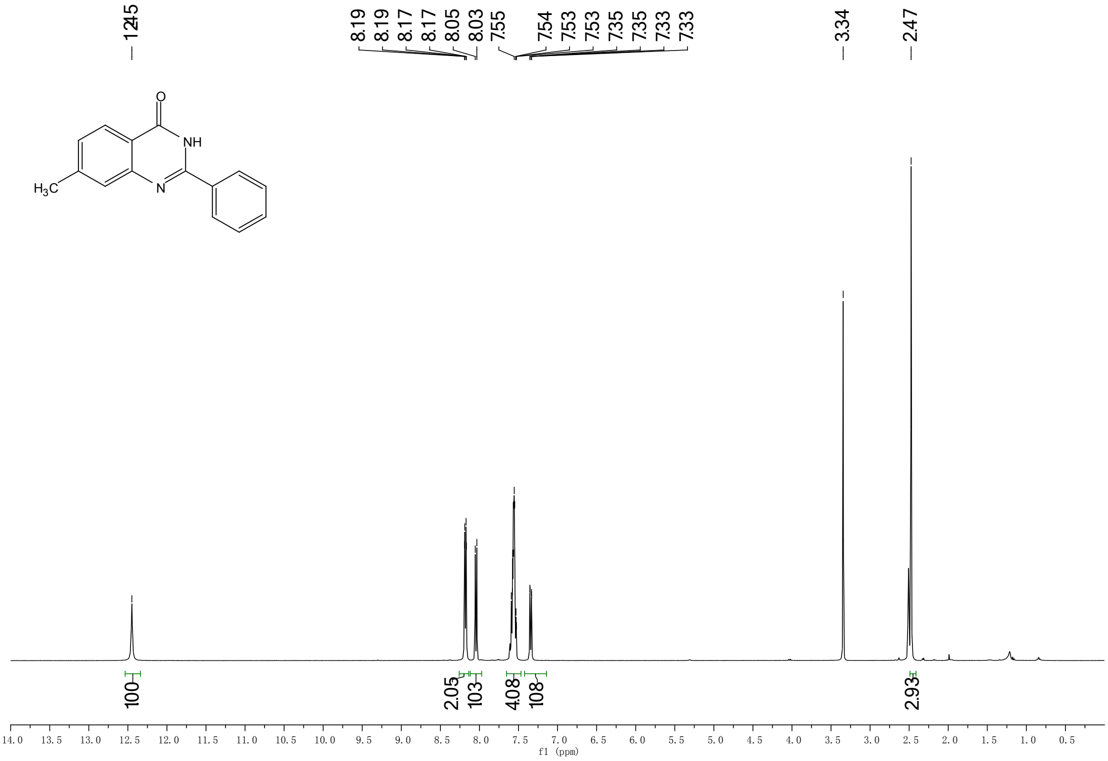
8.19
8.19
8.17
8.17
8.05
8.03
7.55
7.54
7.53
7.53
7.35
7.35
7.33
7.33
3.34
2.47
O
NH
N
H3C
1.00
2.05
1.03
4.08
1.08
2.93
f1 (ppm)

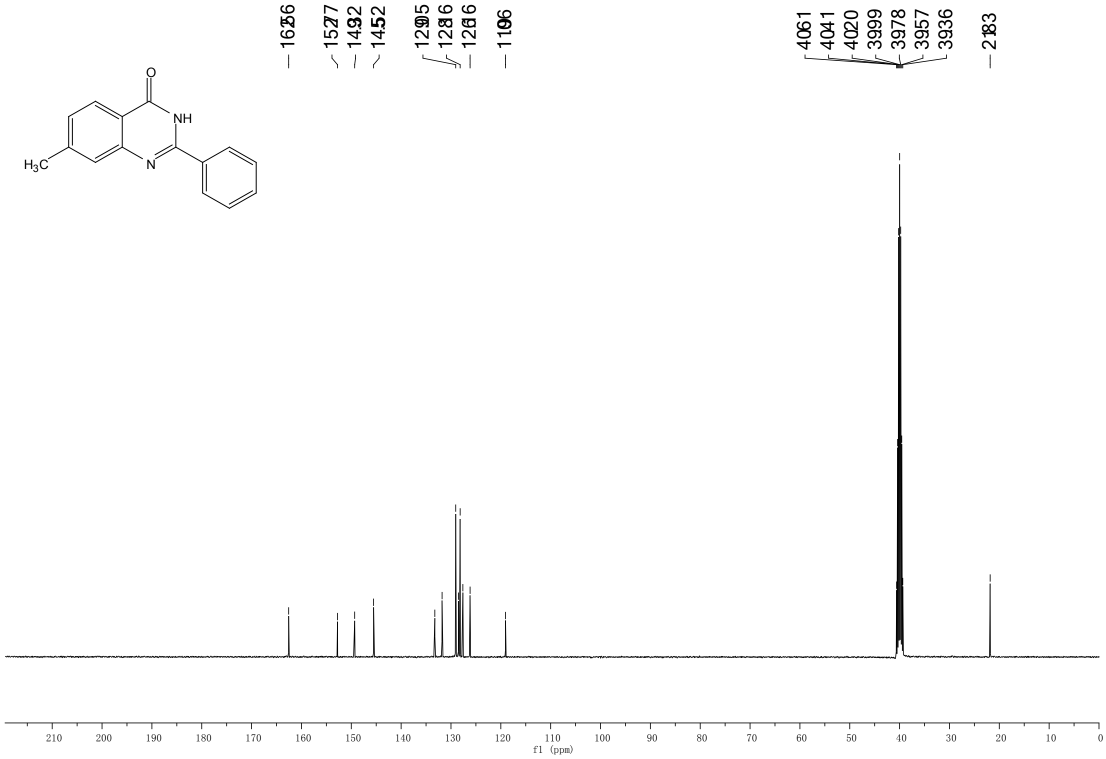
40.61
40.41
40.20
39.99
39.78
39.57
39.36
O
NH
N
H3C
f1 (ppm)

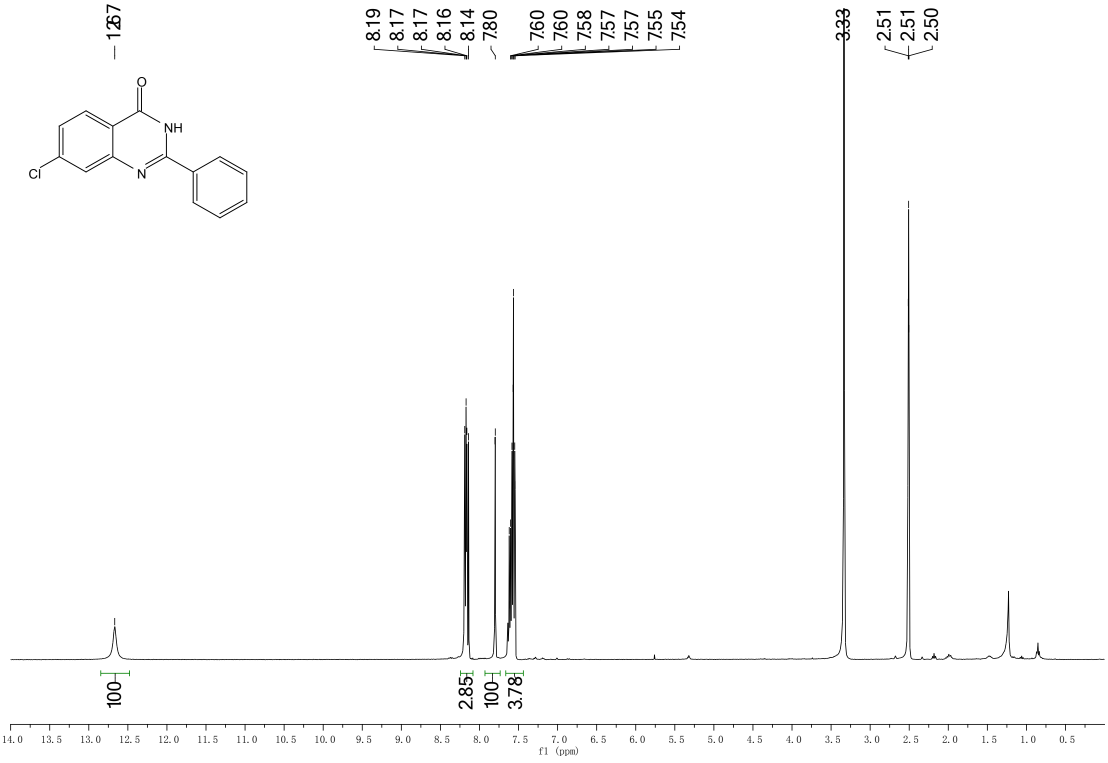

12.67
8.19
8.17
8.17
8.16
8.14
7.80
7.60
7.60
7.58
7.57
7.57
7.55
7.54
3.33
2.51
2.51
2.50
O
NH
Cl
N
1.00
2.85
1.00
3.78
14.0 13.5 13.0 12.5 12.0 11.5 11.0 10.5 10.0 9.5 9.0 8.5 8.0 7.5 7.0 6.5 6.0 5.5 5.0 4.5 4.0 3.5 3.0 2.5 2.0 1.5 1.0 0.5
f1 (ppm)

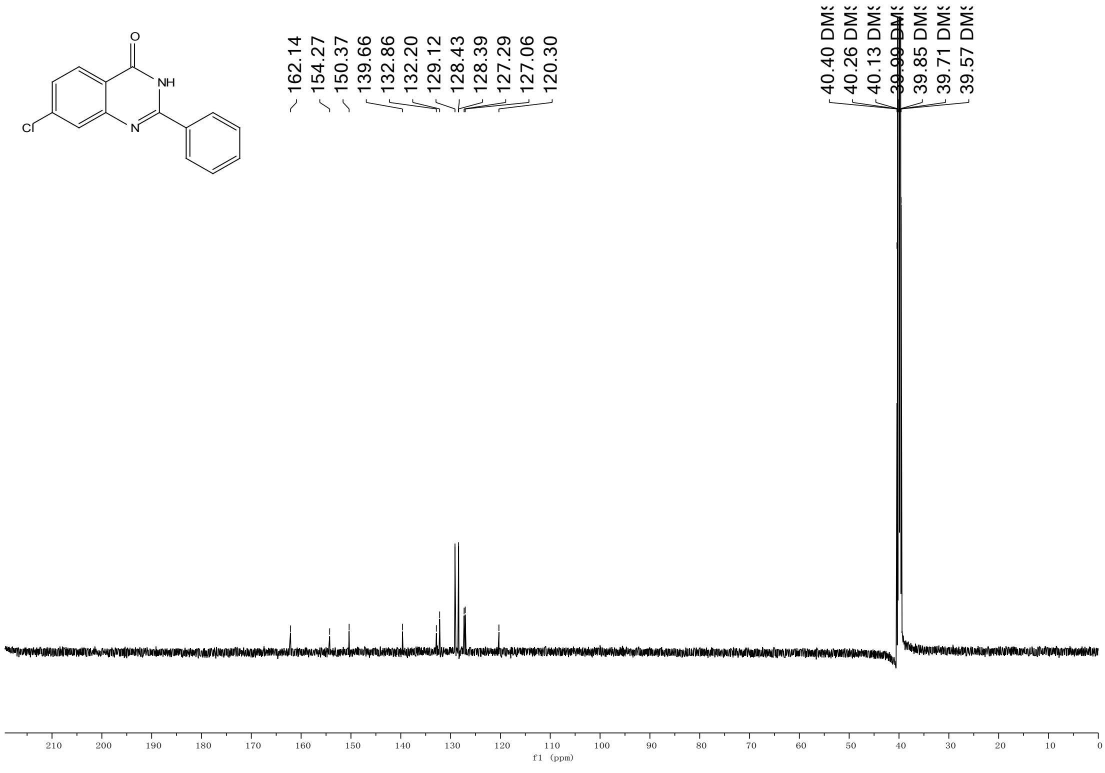

O
NH
Cl
N
162.14
154.27
150.37
139.66
132.86
132.20
129.12
128.43
128.39
127.29
127.06
120.30
40.40 DMS
40.26 DMS
40.13 DMS
39.85 DMS
39.71 DMS
39.57 DMS
210 200 190 180 170 160 150 140 130 120 110 100 90 80 70 60 50 40 30 20 10 0
f1 (ppm)

6. Reference

1. S. Parua, R. Sikari, S. Sinha, G. Chakraborty, R. Mondal, N. D. Paul*, J. Org. Chem.*, **2018**, 83, 11154-11166.
2. M. Saha, P. Mukherjee, A. R. Das, *Tetrahedron Lett.,* **2017,** 58**,** 2044-2049.
3. R. Gujjarappa, S. K. Maity, C. K. Hazra, *Eur. J. Org. Chem.* **2018***,* 33, 4628-4638.
4. Y. Song, K. Zhou, J. Wang, H. Cao, L. Liu, J. Wu, P. Qiu, Q. Xu, *Green Chem.,* **2017***,* 19, 2945-2951.
5. H. Yuan, W. J. Yoo, H. Miyamura, *Adv. Synth. Catal.,* **2012***,* 354, 2899-2904.
6. Z. Wang, T. Song, Y. Yang, *Synlett*, **2019**, 30, 319-324.
7. W. Liu, G. Wu, W. Gao, J. Ding, X. Huang, M. Liu, H. Wu., *Org. Chem. Front.,* **2018***,* 5, 2734-2738.
8. S. Parua, S. Das,, R. Sikari, S. Sinha, N. D. Paul, *J. Org. Chem.,* **2017***,* 82, 7165-7175.
9. Y. Hu, L. Chen, B. Li, *RSC Adv.*, **2016**, 6, 65196-65204.
10. L. Cao, H. Huo, H. Zeng, Y. Yu, D .Lu, *Adv. Synth. Catal.,* **2018**, 360, 4764-4773.
